# Integration of chronological omics data reveals mitochondrial regulatory mechanisms during the development of hepatocellular carcinoma

**DOI:** 10.1371/journal.pone.0256016

**Published:** 2021-08-12

**Authors:** J. Noé García-Chávez, Verónica R. Vásquez-Garzón, Mercedes G. López, Saúl Villa-Treviño, Rafael Montiel

**Affiliations:** 1 Langebio, Unidad de Genómica Avanzada, Centro de Investigación y de Estudios Avanzados del Instituto Politécnico Nacional, Irapuato, Mexico; 2 CONACYT-Facultad de Medicina y Cirugía, Universidad Autónoma Benito Juárez de Oaxaca, Oaxaca, Mexico; 3 Departamento de Biotecnología y Bioquímica, Centro de Investigación y de Estudios Avanzados del Instituto Politécnico Nacional, Irapuato, Mexico; 4 Department of Cell Biology, Center for Research and Advanced Studies (CINVESTAV-IPN), Ciudad de México, Mexico; University of Navarra School of Medicine and Center for Applied Medical Research (CIMA), SPAIN

## Abstract

Mitochondria participate in multiple functions in eukaryotic cells. Although disruption of mitochondrial function has been associated with energetic deregulation in cancer, the chronological changes in mitochondria during cancer development remain unclear. With the aim to assess the role of mitochondria throughout cancer development, we analyzed samples chronologically obtained from induced hepatocellular carcinoma (HCC) in rats. In our analyses, we integrated mitochondrial proteomic data, mitochondrial metabolomic data and nuclear genome transcriptomic data. We used pathway over-representation and weighted gene co-expression network analysis (WGCNA) to integrate expression profiles of genes, miRNAs, proteins and metabolite levels throughout HCC development. Our results show that mitochondria are dynamic organelles presenting specific modifications in different stages of HCC development. We also found that mitochondrial proteomic profiles from tissues adjacent to nodules or tumor are determined more by the stage of HCC development than by tissue type, and we evaluated two models to predict HCC stage of the samples using proteomic profiles. Finally, we propose an omics integration pipeline to massively identify molecular features that could be further evaluated as key regulators, biomarkers or therapeutic targets. As an example, we show a group of miRNAs and transcription factors as candidates, responsible for mitochondrial metabolic modification in HCC.

## Introduction

**Fig 1:**
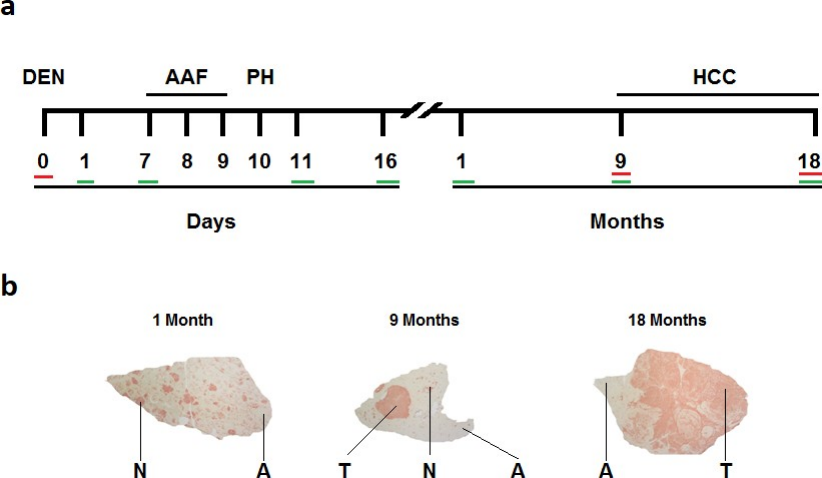
Tissues collected after inducing hepatocellular carcinoma in rats. (**a**) Sampling time points. Green lines under the numbers indicate collection of samples from treated rats. Red lines indicate collection of samples from untreated controls. (**b**) Gamma glutamyl transpeptidase histologic stains used to identify adjacent (A), nodular (N) or tumoral (T) tissues. DEN, diethylnitrosamine; AAF, 2-acetylaminofluorene; PH, partial hepatectomy; HCC, hepatocellular carcinoma.

Mitochondria are cellular organelles involved in multiple cell processes, including the synthesis of ATP by oxidative phosphorylation (OXPHOS) and central processes such as Krebs cycle and apoptosis [1]. Impairment of these functions has been associated with multiple diseases, including cancer [2–4]. Since the past century, Warburg proposed the association between mitochondrial dysfunction and cancer when he described that cancerous cells primarily use glycolysis to obtain ATP, outperforming OXPHOS even in the presence of oxygen [5]. This metabolic change is an important step toward cell malignancy, because aerobic glycolysis provides an advantage for growth and proliferation of cancer cells [6]. Currently, it is known that mitochondria from cancerous cells rewire their metabolism to supply the needs to grow and proliferate by massive anabolism, rather than completely oxidize metabolites to obtain energy [3]. Mitochondria are pivotal in this energetic reorganization that was included in the hallmarks of cancer development proposed by Hanahan and Weinberg [4]. It is known that biochemical pathways must be coordinated to satisfy cellular energetic demands by catabolism or enable anabolic metabolism during proliferation. This coordination includes cytoplasmic pathways and regulatory signaling pathways [7–9] that are not completely understood. In particular, the chronological changes in the interaction between mitochondria and the rest of the cell through signaling pathways and the establishment of metabolic reprogramming remains unclear in cancer development. This is probably due to the lack of a proper model in which the mitochondrial alterations can be followed during cancer progression. The modified resistant hepatocyte model in rats is a model in which the development of hepatocellular carcinoma (HCC) is chemically induced [10]. This model provides an interesting opportunity to study the involvement of mitochondria in very early stages of carcinogenesis and cancer progression. The initial cell damage is controlled, and liver tissues can be sampled throughout the development of HCC. We used this model to chronologically analyze samples collected since the first day to the advanced tumor after eighteen months of starting the experiment, comprising 11 stages and tissues that were compared against controls with no treatment (Table 1 and Figs 1 and S1). We focused on mitochondrial changes at the proteomic level, whole-exome transcriptional expression and key mitochondrial metabolites since very early stages of HCC development, to identify mitochondrial metabolic modifications that support cancerous phenotype. We integrated omics data using pathways over-representation analysis with a weighted gene co-expression analysis (WGCNA). This approach allowed us to identify key pathways and potential regulation during hepatocellular carcinoma development. Furthermore, using supporting vector machine (svm) models we were able to classify samples according to their stage of HCC development.

**Table 1:** Stages of HCC development and tissues sampled. The treatment to which the samples were subjected is shown. DEN, diethylnitrosamine; AAF, 2-acetylaminofluorene; PH, partial hepatectomy. The abbreviations used in the manuscript are shown in the third column. The controls used for each sample are shown in the fourth column.

Sample	Treatment	Abbreviation	Control
Control 0 hours	No Treatment	C0	-
Control 9 months	No Treatment	C9	-
Control 18 months	No Treatment	C18	-
Day 1	DEN	D1	C0
Day 7	DEN & AAF	D7	C0
Day 11	DEN, AAF & PH (Complete)	D11	C0
Day 16	Complete	D16	C0
Month 1 adjacent to GGT + tissue	Complete	A1	C0
Month 1 nodules	Complete	N1	C0
Month 9 adjacent to GGT + tissue	Complete	A9	C9
Month 9 nodules	Complete	N9	C9
Month 9 tumor	Complete	T9	C9
Month 18 adjacent to tumor	Complete	A18	C18
Month 18 tumor	Complete	T18	C18

## Results

### Transcriptional and mitochondrial protein expression during chemically induced HCC development

Rat genome contains 32,833 genes [11]. In rat liver, during HCC development, we recorded the expression of 13,943 (42.46%) genes (S1 Table). We identified 2,687 (19.91%) differentially expressed genes; of these 1,828 (13.11%) were upregulated, 904 (6.48%) downregulated and 45 (0.32%) were either upregulated or downregulated in different stages of the experiment (S2 Fig and S2 Table). The genetic expression in rat liver was most affected in the first three evaluated stages of HCC development, where carcinogenic treatment was administered. The first day, during DEN administration, was the stage with the most differentially expressed genes (DEG) (2,034).

At mitochondrial proteomic level, in all controls and samples, we identified 1,577 proteins (S3 Table) with a match to a single protein from the rat proteome database [12], which comprises 29,998 proteins. In mitochondria from nine-months nodules and from tissues at day seven, we identified the lowest number of proteins with 661 in each case. As AAF arrests hepatocyte proliferation [13], these few expressed mitochondrial proteins in day seven are probably important to cell survival but not involved in proliferation. In control samples, we identified the highest number of proteins, with more than 1,000 identified proteins in each control sample (S4 Table). In our contrasts, 363 proteins (22.02% of total identified) were differentially expressed throughout HCC development (S5 and S6 Tables). Contrary to data observed in gene expression, there were more subexpressed mitochondrial proteins (281, 17.7%) than overexpressed ones (60, 3.78%). In addition, 35 (2.2%) differentially expressed proteins (DEP) were either subexpressed or overexpressed in different stages (S2 Fig). The stage with the most DEP (114) was the first day in response to DEN administration, in agreement with transcriptomic data. The stage with the second most DEP was nine-months tumor, with 80, followed by the adjacent tissue at one month, with 39, an eighteen-months tumor, with 38.

### Supervised classification models of HCC development stage

**Fig 2:**
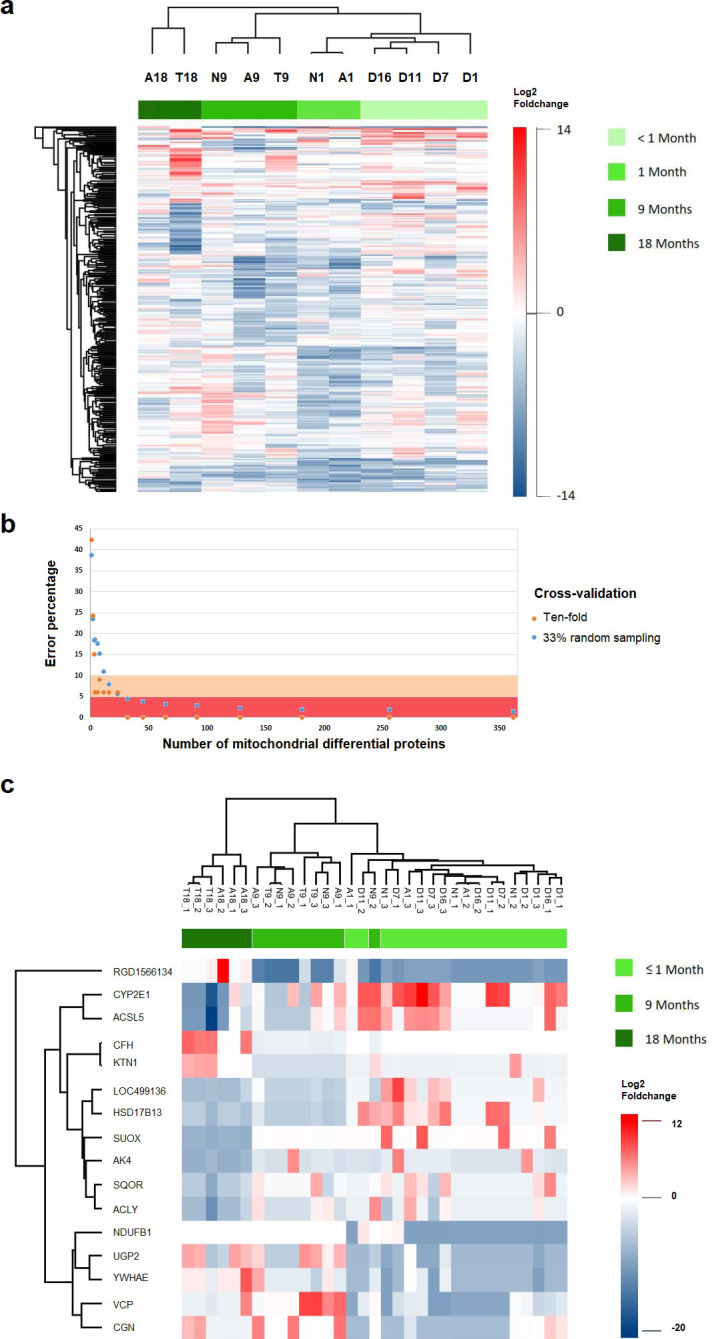
Proteomic expression profiles and its use for the models of samples classification according to stage of HCC development. (**a**) Pearson correlation-based hierarchical clustering using all differentially expressed proteins (N = 363); the stage of HCC development is shown in horizontal green scale on top. (**b**) Percentage of error of our models in classification of HCC development stage (*y*-axis) in relation to the number of proteins used (*x*-axis); pink area represents 10% of error and red area 5% of error; orange points represent ten-fold cross-validation and blue points 33% random sampling cross-validation, in both cases 250 repeats were used. (**c**) Independent classification of the HCC development stage of 33 samples (horizontal green scale on top) using the 16 disclosed proteins in our classification models.

With the Pearson coefficient correlation for proteomic profiles clustering, we obtained clades that contained samples from the same stage of HCC development (Fig 2A). With these results, we decided to evaluate to what extent proteomic profiles predict the stage of HCC, using two support vector machine (svm) models, as described in methods. Using all DEP information (S7 Table) for stage classification in our svm models, almost all samples were classified correctly (with an error of 1.45%). Only the sample T9_1 from category 9 was classified into two categories, 9 and 18 (S8 Table). It is remarkable that with only 16 proteins (SUOX, CFH, NDUFB1, LOC499136, RGD1566134, YWHAE, HSD17B13, VCP, AK4, UGP2, KTN1, CYP2E1, SQOR, ACSL5, ACLY, CGN) it was possible to correctly classify more than 90% of samples, 7.9% and 6.06% of error with two classification models (see Methods) (Fig 2B and S9 Table). Then, considering the fold change of these 16 proteins only, we independently confirmed they were good enough to classify the 33 samples (Fig 2C) with a low error rate. Only the sample N9_2 was misclassified into the first month group (3% of error in this case).

### Annotation of differentially expressed genes and proteins

In total, 145 KEGG [14, 15] pathways were over-represented among DEG and mitochondrial DEP, 110 of them (75.9%) were over-represented only at transcriptomic level, 15 (10.3%) only at mitochondrial proteomic level and 20 (13.8%) were over-represented at both levels (S10 and S11 Tables).

Most over-represented pathways were observed during the treatment stages. In the first evaluated stage, after DEN administration, 115 pathways were over-represented at transcriptional level. In this stage, we identified a clear pattern, in which upregulated pathways were related to signaling pathways, several types of cancer and other diseases, while downregulation was related to metabolism. Upregulation includes several cancer-associated signaling pathways, such as p53, TNF, NF-kappa B, MAPK, HIF-1 and AGE-RAGE, but also includes signaling pathways of T cells, B cells, Toll-like, NOD-like, C-type lectin and cytokine-cytokine receptors. It is of note that AGE-RAGE signaling pathway was upregulated during the first 5 stages, with probably a relevant role in HCC carcinogenesis. Besides, the pathway of microRNAs in cancer was also upregulated during the three first evaluated stages, as well as the platinum drug resistance pathway in days one, seven and eleven, and in nodules and tumors at nine months.

On the other hand, during day one we observed downregulation almost exclusively in pathways related to metabolism at transcriptomic and mitochondrial proteomic levels. Downregulated pathways were related to metabolism of 17 amino acids, including tryptophan, glycine, serine, threonine, beta-alanine, lysine, alanine, aspartate, glutamate, cysteine, methionine, arginine, tyrosine, proline, histidine, valine, leucine and isoleucine and branched-chain amino acids. In addition, PPAR signaling pathway, glyoxilate, dicarboxylate, butanoate, retinol, lipids and fatty acids related metabolism, were also downregulated. Of these, PPAR signaling, butanoate and fatty acid metabolism were maintained downregulated in day eleven after the partial hepatectomy, when proliferation is induced; however, also these same pathways were downregulated in late tumor, when tumor proliferation is higher, suggesting an important role for the modulation of this process.

### Metabolic profiles

Metabolites analyzed here participate as intermediates of the TCA cycle (citric acid, malic acid, succinic acid, alpha-ketoglutaric acid and fumaric acid) or in anaplerosis (glutamic acid) and have a central role in cancer. For example, citrate has been demonstrated to participate in *de novo* synthesis of fatty acids in cancerous cells [16]. Also, in several types of cancer, glutamine replenishes carbon to TCA cycle when pyruvate uptake into mitochondria is perturbed [17]. Furthermore, in recent years, various functions have been assigned to metabolites of the TCA cycle, such as signaling molecules relevant to chromatin remodeling, DNA methylation or response to hypoxia [18, 19]. Thus, we decided to include metabolites of the TCA cycle and glutaminolysis in the present study to identify differential concentrations throughout HCC development and whether these changes are correlated with tumoral phenotype or enriched pathways.

**Fig 3:**
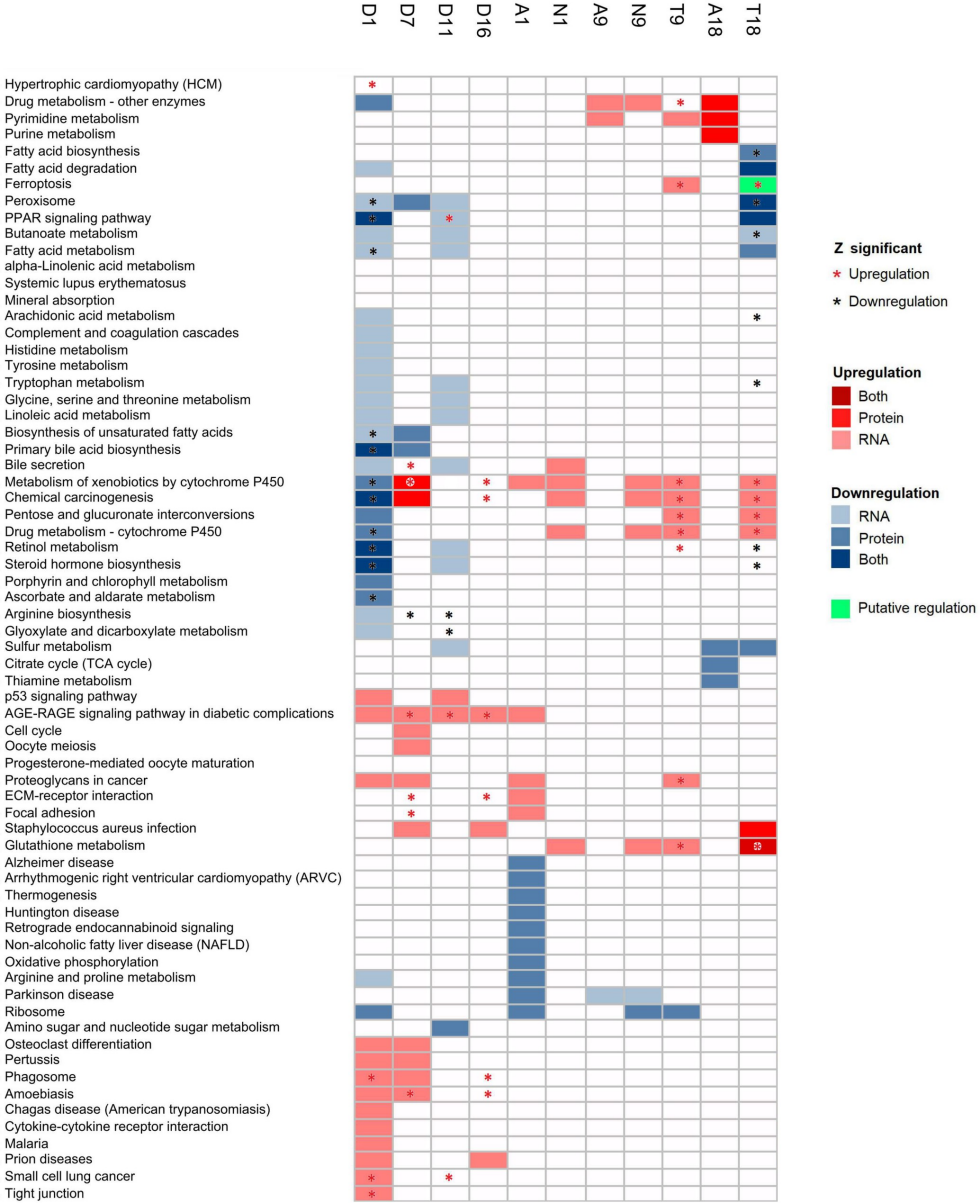
Pathway over-representation-based Integration using Biological level expression (PIB) and Stouffer sum Z. Statistically significant pathways are shown in a grid with cells in a color corresponding to upregulation (red scale) or downregulation (blue scale). Pathways with weighted Z score <0.05 are shown with an asterisk, red if the pathway was upregulated and black if it was downregulated.

We identified that malic acid concentration was increased during days 1 (*p* = 0.06), 7 (*p* = 0.02), 11 (*p* = 0.038), and 16 (*p =* 0.068), ranging from 1.59 to 4.63 in fold change. Furthermore, during day 1, significant decreased levels of glutamic (fold change -3.95, *p =* 0.003), citric (fold change -12, *p* = 0.0003) and alpha-ketoglutaric acids (fold change -17.8, *p* = 0.000004) were observed. It is unclear why these metabolites decrease in concentration; however, in day 1 we observed numerous metabolic pathways being downregulated (Fig 3). During the seventh day, when mitosis is inhibited, the levels of glutamic (fold change 3.37, *p* = 0.036) and malic acids (fold change 4.63, *p =* 0.022) were significantly increased. Although we measured metabolites levels in more advanced stages, their concentration levels were not significantly different when compared with controls, possibly because we were not able to obtain data from all replicates (two replicates of adjacent tissue of one month, one replicate of the adjacent tissue at nine months, and one replicate of the control at eighteen months were not useful) (S12 Table). More studies are needed to obtain more information regarding chronological changes of TCA metabolites during HCC development.

### Pathway over-representation-based Integration using Biological level expression (PIB)

With PIB analysis we can identify pathways with consistent expression, this means upregulated or downregulated in both transcriptomic and proteomic levels, suggesting that their effects in cellular function have an important role in the disease [20]. We identified 67 distinct over-represented pathways (Fig 3 and S13 Table). As several of them were enriched in two or more stages of HCC development, these pathways were over-represented a total of 131 times among samples. Most of the times (122, 93.13%), the expression of these pathways was not directionally consistent at both transcriptomics and proteomics levels. This is not surprising, since it is well known that correlation between the expression of transcripts and proteins is in general low due to several post-transcriptional, translational and degradation mechanisms [21, 22].

In nine occasions (6.87%), pathway over-representation patterns at transcriptomic and proteomic levels were consistent. These pathways occurred in two stages of HCC development, namely during DEN administration and in tumor samples of eighteen months. Interestingly, 8 out of the 9 detected consistent pathways were downregulated and were related to metabolism, including PPAR signaling pathway, steroid hormone biosynthesis, retinol metabolism, primary bile acids biosynthesis, peroxisome and fatty acids degradation. The unique upregulated consistent pathway was glutathione metabolism in tumor at eighteen months. On one occasion during the last evaluated stage, transcriptomic and mitochondrial proteomic information was discordant, in the ferroptosis pathway. Data obtained from DEG and DEP suggest putative post-transcriptional regulation at eighteen months tumor in this pathway (green color in Fig 3), probably by translational repression or degradation of pathway proteins, since we observed upregulation at transcript level but downregulation in proteins [23–26]. Another explanation is that 15–30% of mitochondrial proteins are localized also in other organelles than mitochondria [27, 28], which could explain the (apparent) downregulation at mitochondrial proteomic level.

### Pathway over-representation-based integration by weighted Z test (Z)

With the information obtained in PIB we calculated two *p* values for each pathway, the first using transcriptomics and the second using mitochondrial proteomics. These *p* values were combined using a weighted sum of Z as described in methods. With this approach, we identified presumably relevant pathways that are directionally consistent at two biological levels in HCC development. We identified 34 over-represented pathways and 57 over-representation events, of which, 19 had not been detected without the combination of *p* values (S14 Table). When using Z, there were not statistically significant over-represented pathways in nodules or its adjacent tissues in samples collected during months one or nine (Fig 3).

### Omics integration based on Weighted Gene Co-expression Network Analysis (WGCNA)

**Fig 4:**
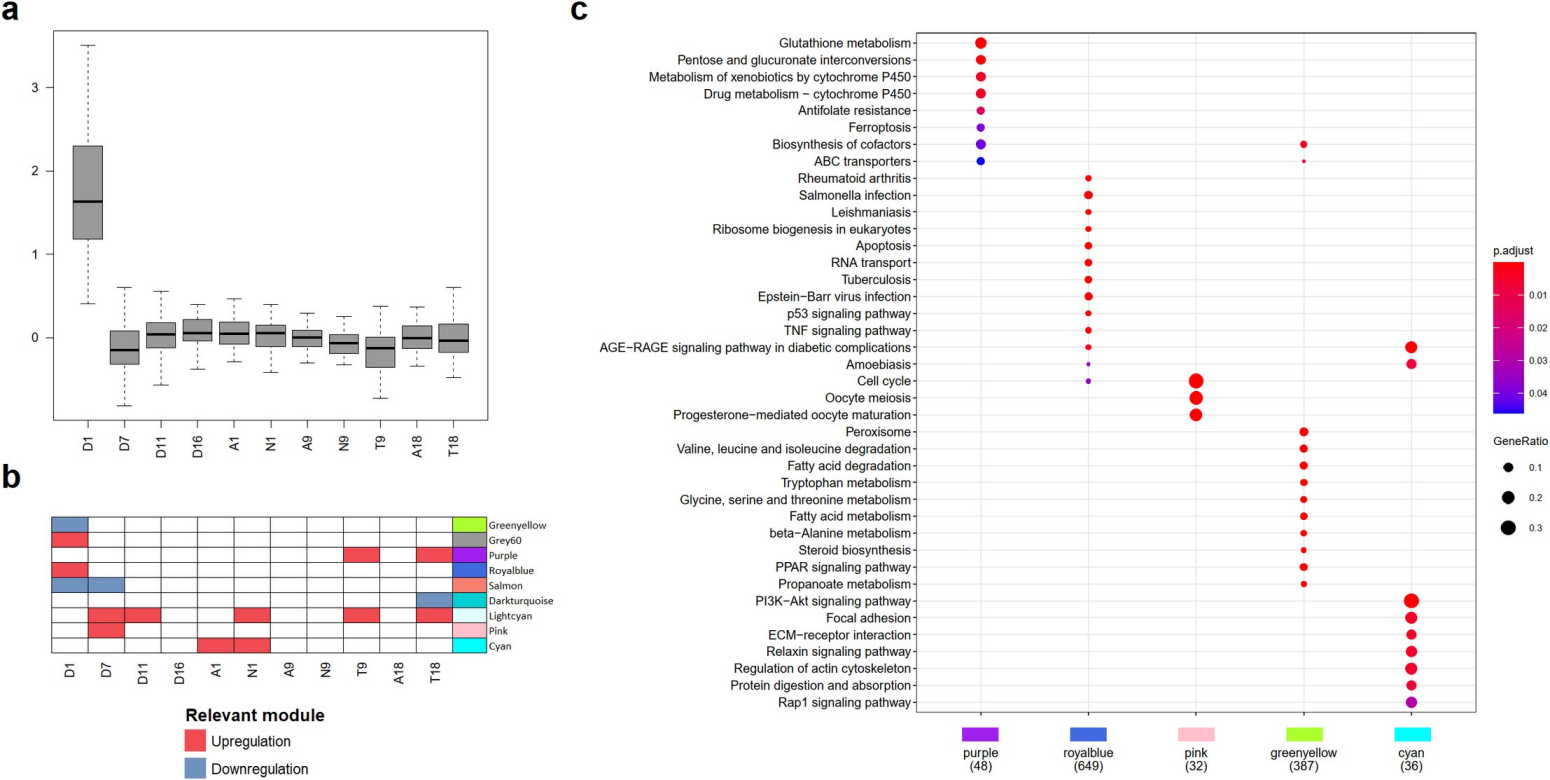
Transcriptomic modules correlate with specific stages of HCC development indicating a potentially relevant role for these modules. (**a**) Grey60 transcriptomic module was upregulated during DEN administration in day one. (**b**) Relevant modules in specific stages of HCC development, upregulated modules are shown in red color, whereas blue shows downregulated modules. (**c**) KEGG pathways over-represented in transcriptomic modules, numbers under module color are the number of genes used in over-representation pathway analysis. Module identification was performed in R using *WGCNA* package and over-representation pathway analysis, using *clusterProfiler* package, considering a *p* value < 0.05 and *q* value <0.1.

When we analyzed the expression profiles of DEG, we identified 9 transcriptional modules throughout HCC development, these modules were labeled using color names as described in S15 Table. We analyzed the distribution of DEG fold change values in each module to identify their relevance in specific tissues or HCC stages. We considered a module to be relevant for the evaluated stage if the median of log fold change values in the module was >|0.9| and the distribution of the values were significantly different from the other stages (Wilcoxon test, p-value < 0.05) (Fig 4A and 4B). Relevant downregulated and upregulated modules identified at each stage can be seen in Fig 4B. We identified over-represented pathways in 5 modules (purple, royalblue, pink, greenyellow and cyan) (Fig 4C and S16 Table); hence, we were able to identify transcriptionally regulated pathways and functions relevant to specific stages of HCC. For instance, in day–one tissues, royalblue module was relevant with upregulation of apoptosis, RNA transport, ribosome biogenesis, cell cycle, AGE-RAGE, TNF and p53 signaling pathways. On the other hand, greenyellow module showed downregulation in PPAR signaling pathway, primary bile acid biosynthesis, fatty acid metabolism and degradation, valine, leucine and isoleucine degradation, glycine, serine, threonine metabolism, tryptophan metabolism, biosynthesis of cofactors, among others (Fig 4B and 4C).

Cyan module was relevant in one-month tissues, with upregulation in focal adhesion, ECM-receptor interaction and AGE-RAGE, Rap1, Relaxin, and PI3K-Akt signaling pathways. Pink module upregulation was relevant in day seven, with the following over-represented pathways: cell cycle, progesterone-mediated oocyte maturation and oocyte meiosis, and osteoclast differentiation. Purple module was upregulated in tumors of nine and eighteen months and includes the pathways of ferroptosis and metabolism of pentoses, glucuronate, glutathione, biosynthesis of cofactors, ABC transporters and metabolism of xenobiotics and drugs by cytochrome 450 (Fig 4B and 4C).

Furthermore, salmon module was downregulated during days one and seven, whereas grey60 module was upregulated in day one and lightcyan was upregulated in several stages, including days seven and eleven, nodules of one month and tumoral tissues from nine and eighteen month. Finally, darkturquoise module was downregulated in tumor of eighteen months. However, none of these modules had over-represented pathways (Fig 4B). With an enrichment analysis on sequences upstream to genes included in each module (FDR<0.05), we identified binding motifs of transcription factors (TF) that probably participate in transcriptional regulation of the genes in each module. We identified enrichment in tens of TF binding motifs in 8 transcriptomic modules. We considered as the most important those binding motifs in which their TF was differentially expressed or was present in the same module, therefore, only those TF genes are mentioned here. All enriched binding motifs and their corresponding TF from all modules are described in S17 Table. In the royalblue module we found 3 TF binding motifs corresponding to differentially expressed TFs present in the same module, Egr1, Etv3 and Hes1. Egr2, Ehf and Klf12 binding motifs were also enriched but their corresponding TFs were not part of the module. Nevertheless, we identified these TF in the modules cyan (Ehf), greenyellow (Klf12), and grey60 (Egr2). Notably, TF binding motifs enriched in the grey60, royalblue, greenyellow, pink, cyan, lightcyan, and purple modules were enriched for one or several TF binding sites recognized by Egr1, Egr2, Ehf, Etv3, Hes1 or Klf12.

Furthermore, in each module without enrichment in binding motifs we searched for TFs present in the same modules and identified Tead1 and Sox9 in lightcyan module; Vdr in purple module; Ddit3, Hif1a, Irf1, Max, Myc, Nfe2l2, Nr4a2, Rel, Rela, Runx1, Spi1, Stat3, Tbp, and Tp53 in royalblue module. Using genetic network analysis (see methods) we identified that Myc and Runx1 were connected respectively to other 10 and 7 of the transcription factors of the purple module. Mafb was present in salmon module, and Cebpa, Pbx1 and Sox5 were present in greenyellow module.

**Fig 5:**
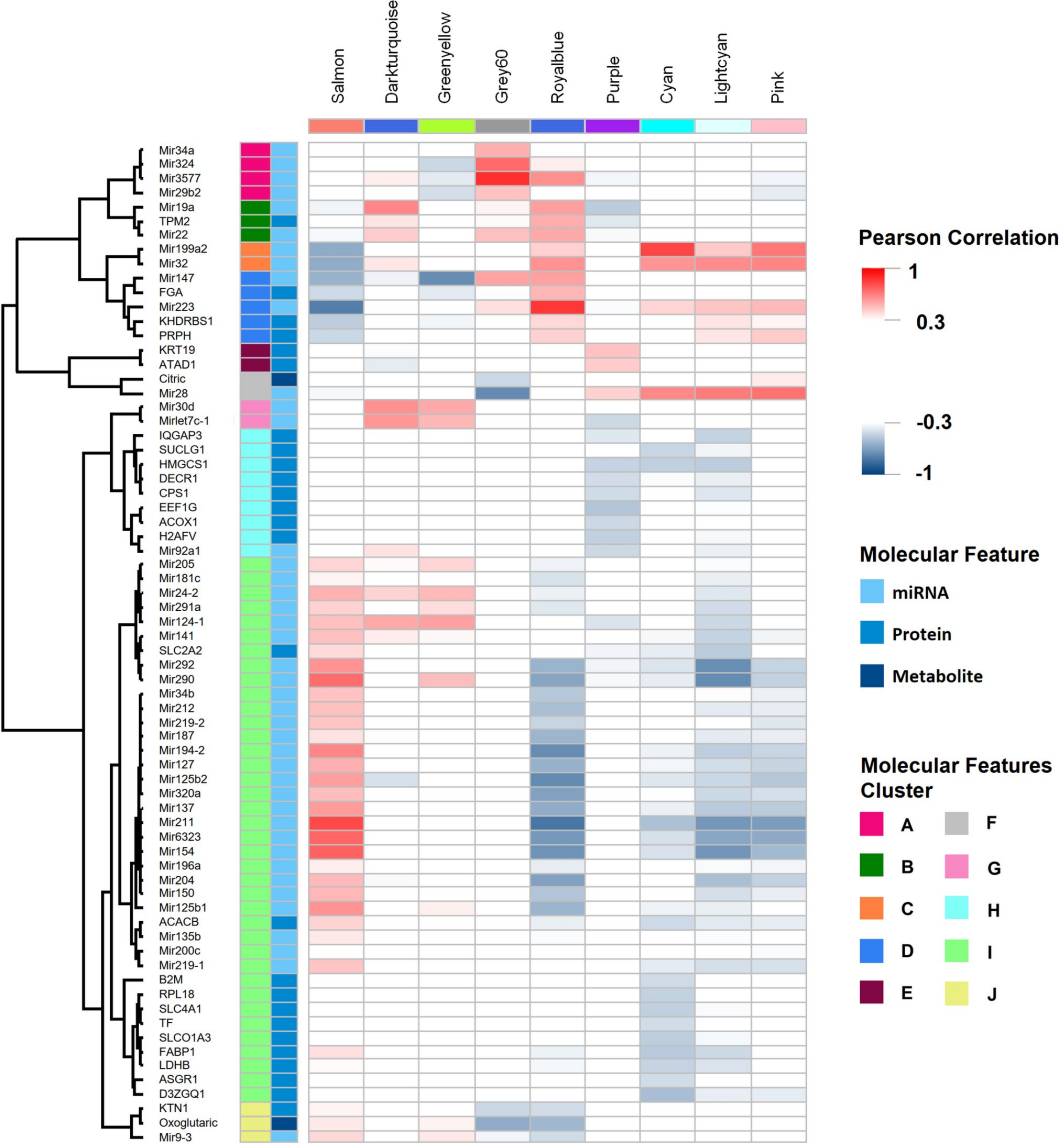
WGCNA-based omics integration is useful to identify hypothetical regulation mechanisms between biological levels. Nine modules of co-expressed genes were identified using WGCNA and correlated with profiles of miRNAS genes, proteins and metabolites (first column in blue scale) throughout HCC development; molecular features are shown in rows, molecular features were clustered (second column, colored clusters A-J) according to their expression profile using Pearson’s correlation. Only molecular features with a significant Pearson correlation > |0.40| and *p* <0.001 are shown. Transcriptomic modules are shown in the top of each column. Negative correlated molecular features in the grid are shown in blue, while those positively correlated are shown in red.

To massively identify candidate processes associated with levels of metabolites and with putative regulators at different biological levels, we correlated profiles of transcriptomic modules with the expression of the following molecules: mitochondrial proteins, metabolites, and miRNAs genes (S18 Table), hereafter referred to as molecular features (see methods). In total, we analyzed 4,014 pairs of molecular features and transcriptomic modules, identifying 159 (3.96%) significant correlations (*p*. <0.001) that represent 159 putative regulation processes. These 159 correlations were related to 70 molecular features, including 2 metabolites, 42 miRNAs and 26 mitochondrial proteins, some of them correlated with various modules. Molecular features were clustered according to the Pearson correlation distance with a pre-established parameter k = 10. Each cluster of molecular features was labeled using letters A to J (Fig 5). Pearson’s correlation values and statistical significance for each module with all molecular features are shown in S4 Fig (S19 Table).

Metabolites of the tricarboxylic acids (TCA) cycle can participate in chromatin modifications, DNA methylation and post-translational modifications [18]. Using WGCNA we identified two possible scenarios in which these metabolites may participate in transcriptomic regulation: i) putative regulation of gene expression mediated by metabolites and ii) a group of co-expressed genes which possibly modify metabolites level. Two cases can exemplify these scenarios, the negative correlation of oxoglutaric acid with royalblue module (-0.55, *p* = 9e-06) and grey60 module with citric acid (-0.44, *p* = 8e-04). However, most metabolites and modules had no significant correlation.

Also, we were able to identify candidate molecules that may participate in putative regulation processes. For instance, greenyellow module was negatively correlated with miR147 (-0.71, *p* = 1e-09). We identified over-representation in several pathways of greenyellow module genes, which includes steroid biosynthesis, fatty acid and BCAA degradation, tryptophan, and propanoate metabolism, as well as PPAR signaling pathway; thus, these pathways could have been downregulated by miR147. Moreover, it is possible to identify groups of molecular features correlated with transcriptional modules (e.g., proteins from cluster I had a significant negative correlation with cyan transcriptional module, also several miRNAs (miR211, miR125b2, miR194-2, miR6323, miR154, among others) from the same cluster I had negative correlation with royalbue module. These results are just a few examples of all the candidate molecules identified with individual significant correlation with each transcriptomic module (Fig 5).

## Discussion

In this work, for the first time and using an integrative approach we chronologically analyzed mitochondria throughout DEN-induced carcinogenic process in a rat model. DEN-induced HCC has been previously shown to have similar hepatic alterations that resemble the progression of human chronic liver disease to HCC. Mice subjected to DEN treatment develop histological modifications including neutrophil infiltration, bile conduct proliferation, centrilobular hemorrhagic necrosis, and bridging necrosis [29]. Nevertheless, fibrosis and cirrhosis, a common feature of human HCC, are not observed in our model. However, as the initiation is controlled, tissue sampling can be designed to study very early stages of carcinogenesis, thus having the opportunity to study mitochondrial function during the process in more detail. In total, we analyzed 11 stages, from the first day of carcinogenic treatment to late tumor at eighteen months after initiation. We focused on determining whether mitochondrial metabolic rewiring occurs progressively with cumulative alteration since early stages, or suddenly, when tumor phenotype is already observed. We integrated comparative mitochondrial proteomics and metabolic profiles with whole-exome transcriptomics, combining pathway over-representation tests and WGCNA to identify correlated molecules that may have relevant roles in mitochondrial regulation during disease progression.

During the first 5 stages analyzed, we observed upregulation of the receptor of advanced glycation end products (AGE-RAGE) signaling pathway. This upregulation was detected using both transcriptomic and mitochondrial proteomic information, as well as with their integration (Fig 3). This pathway has been suggested as a potential target for therapeutic intervention in HCC patients, because it was associated with proliferation induction in HCC, and with sorafenib resistance via AMPK/mTOR signaling pathway [30]. Our analysis indicates an active role of AGE-RAGE signaling pathway during HCC carcinogenesis.

Our integrative approach allowed us to identify several molecules, TF binding motifs and altered pathways that have been previously related to HCC or other cancers. However, as we collected samples since the first day of HCC development, our results are relevant in the study of carcinogenesis and the role of mitochondria in the process. We identified several alterations and candidate molecules relevant for carcinogenesis since very early stages of HCC development. For example, in the royalblue module we identified three TFs, Egr1, Etv3 and Hes1, that recognize enriched TF binding motifs of genes of the same module, as well as Egr2 in grey60 module. EGR1 is transcribed downstream in AGE-RAGE signaling and activates expression of p53 and TGFB1 [31]. EGR2 induces apoptosis in several cancer cell lines [32], ETV3 contributes to growth arrest [33], HES1 is involved in DNA interstrand crosslink damage repair [34] and could participate in response to DNA adducts induced by DEN. Taken together, this information suggests that these TFs could orchestrate the hepatocyte response to chemical damage produced by DEN. However, as previously shown [35], HES1 allows cancer cells to evade differentiation and irreversible cell cycle arrest, indicating an active role of HES1 in the carcinogenic process. Additionally, during day one, we observed a massive metabolic downregulation, at transcriptomic and mitochondrial proteomic levels (Fig 3), putatively mediated by genes of greenyellow and salmon modules. Greenyellow TFs Cebpa, Pbx1 and Sox5, have relevant roles; Sox5 as potential biomarker of HCC [36] and Pbx related to cell stemness [37]. CEBPA coordinates gluconeogenesis, lipogenesis, proliferation arrest, and differentiation of hepatocytes [38–43]. Low CEPBA/CEPBB ratio, as observed in different stages of our experiment, is related to undifferentiation, proliferative state of hepatocytes, and development of tumors [41–45]. On the other hand, CEBPA upregulation suppresses Hes1 activation, and overexpression of Hes1 partially abolished the anti-proliferation effect of CEBPA [44]. CEPBA and HES1 have antagonistic roles in proliferation in HCC and both TFs must be under a delicate regulation in order to maintain metabolic and homeostatic functions of cell cycle progression [42]. We detected this antagonistic correlation between Cebpa and Hes1 expression in our experiment. More studies are warranted to evaluate CEPBA and HES1 as key TFs in early stages of carcinogenesis. Finally, the expression of greenyellow and royalblue modules, where Cepba and Hes1 are present, were negatively correlated throughout HCC development, and they also had an inverse correlation with the same clusters of miRNAs and proteins (Fig 5). These modules probably have an antagonistic regulation, and as WGCNA integration suggests, their regulation could be mediated upstream by miR147 (Fig 5). More studies are necessary to understand the antagonistic expression of the signaling and metabolic pathways over-represented in these modules.

Other relevant TFs with enriched binding motifs were identified: Ehf, in cyan module, influence recruitment of neutrophils during progression of hepatocellular carcinoma [46]. Mafb (salmon module) was expressed at low levels while Maff at high levels in nodules after one month and in tumors, similar results have been described before [47]. Also, in tumoral tissues, we observed the upregulation of purple, and lightcyan modules, the latter also relevant in days seven and eleven and in nodules of one month. TFs Tead1 and Sox9 were present in the purple module, whereas Vdr was in lightcyan module. TEAD1 is associated with aggressiveness [48], as well as high levels of SOX9 with poor survival in HCC patients [49]. Vdr appears to be an indicator in the development of HCC among HCV-infected patients [50]. Also, we identify previously not described molecules that could have an active role in HCC development. For example, TFs Klf12 and Sp40 were present in the pink module and genes in the module were enriched in binding sites that these TFs recognize, thus these TFs could be regulating genes of this module. More studies are needed to understand the participation of these TFs in carcinogenesis or in supporting cancer development.

Our proteomic analysis revealed that mitochondria are dynamic organelles with stage-related responses during HCC development. Mitochondrial DEP profiles were specific to the stage of HCC development. Also, and surprisingly, we found that mitochondria of adjacent tissues had an identifiable proteomic expression profile correlated with their adjacent affected tissue counterparts in the same stage of HCC development. This occurs from the first month after initiation. These results suggest that there is a communication mechanism between mitochondria from both tissues within the liver that makes more similar their regulation of mitochondrial protein expression. This communication can be either one-way, from affected to adjacent tissues, or two-way implying feedback. Communication for this shared regulation could be mediated by several mechanisms: metabolites or proteins produced by affected tissues and secreted to the cell microenvironment (e.g. tumor-excreted lactic acid); transporting of miRNAs, mitochondrial enzymes, metabolites and even complete mitochondrial genomes in tumor exosomes [51]; and mitochondrial migration from affected cells to adjacent cells [52]. Circulating cell-free competent mitochondria have been found in blood, suggesting a role in signaling and cell to cell communication for these organelles [53]. It would be difficult to detect this mitochondrial communication using cell lines or tumor biopsies. Also, in HCC with focal origin, the communication signal will be diluted in a positive correlation with the distance to the nodular or tumoral tissue, making it more difficult to detect. On the other hand, the multifocal origin of HCC in our rat model distributes these signals across the liver facilitating its characterization; therefore, our model provides an opportunity to study these mechanisms in more detail.

Because the proteomic profiles of adjacent tissues are more similar to their adjacent tissue counterparts than to any other tissue from other HCC stages, our method could help in the HCC diagnosis and provide information on the stage of development using information of hundreds of mitochondrial proteins. Furthermore, our svm models shows that it is possible to correctly classify 92% of the samples according to their HCC stage using information from only 16 proteins and from adjacent tissue samples, making unnecessary the collection of tumor or nodular samples. However, we used a relatively low number of samples (33) and our model might present some problems of overfitting. Therefore, we propose to further investigate these candidates in humans to evaluate whether these are useful in stage classification or as a diagnostic method in patients with high risk and genetic predisposition to develop HCC. Mitochondrial proteomic expression profiles represent a new potential screening method for early diagnosis, to be evaluated in human HCC with multifocal origin, such as HCC caused by nonalcoholic steatohepatitis.

The results of our chronological analysis of metabolic pathways suggest that from very early stages, mitochondrial metabolism is modified. We observed a massive metabolic downregulation since day one, very likely in response to chemical treatment; however, once carcinogenic treatment was ended, several mitochondrial pathways were modified at transcriptomic or proteomic level. This change not only occurred in nodules or tumor samples but was also detected in adjacent tissues from the first month after DEN administration and throughout the development of HCC (Fig 3). Remarkably, we observed tumoral phenotypes after nine months but not at five months (S1 Fig), which suggest that the mitochondria actively participate in the carcinogenic process, and that the modifications observed are possibly aimed at satisfying the proliferative needs of pre-neoplastic nodules and its progression into tumors. In this study, and in agreement with previous studies in mice model [54], we observed downregulation of lipids-related degradation pathways, probably to support cell membrane synthesis by blocking fatty acids degradation in mitochondria [55, 56]. Similar downregulation in lipids metabolism has been reported in human HCC samples of The Cancer Genome Atlas and it was correlated with reduced survival [57]. Several over-represented pathways were stage-specific, suggesting that mitochondria are highly dynamic organelles, with differential roles during HCC development according to the stage of disease development. Difficulty in obtaining an early diagnosis also makes it difficult to obtain tissue samples in the early stages, which may explain why other studies have missed the pathways, proteins, and transcripts that we detected in these stages.

WGCNA was useful to identify putative key regulators in several of the evaluated stages, e.g. antagonistic expressions of Cepba and Hes1 are likely to participate in carcinogenesis, perhaps promoting a metabolic state of the cell that enhances proliferation and undifferentiated behavior by signaling and metabolic regulation. Likewise, antagonistic expressions of miRNAs and transcriptomic modules were also interesting. For example, miR28 were negatively correlated to blue module that include apoptosis, cell cycle p53 and AGE-RAGE signaling pathways, indicating a probable regulatory role. Furthermore, miR147 was identified as candidate for downregulation of modules that showed over-representation in degradation of fatty acids, BCAA and tryptophan, and PPAR signaling pathways. Additionally, miR223 was negatively correlated with salmon cluster, and previous analysis suggested miR223 as an HCC biomarker [58]; hence other candidate miRNAs identified with our approach could also be relevant. Therefore, WGCNA-based integration can be useful to guide experimental tests to know the role of novel genes, proteins and metabolites in cancer development.

## Conclusion

We developed a pipeline to analyze and interpret complex data from different omics obtained from multiple time points and tissues of DEN-induced HCC. We applied this pipeline to describe the role of mitochondria throughout cancer development, as well as to shed light on the very early stages of the carcinogenic process and its progression towards advanced stages of HCC. Our analysis suggests that mitochondria are dynamic organelles with metabolic modifications that precedes tumor phenotype and are related to each stage of HCC development with specific regulation. Using mitochondrial omics integration is possible to massively identify novel candidates that could be further evaluated as regulatory mediators, therapeutic targets and biomarkers. Furthermore, using mitochondrial information and a svm model we were able to classify each stage of HCC development. This classification method may have potential applications in diagnosis and precision medicine warranting further efforts. Also, WGCNA as an omics integration method could be useful to investigate whether mitochondrial characteristics observed herein are correlated with prognosis or clinical features to provide a better clinical management. These strategies can be used in similar multiomics projects.

## Methods

### Chemical induction of hepatocellular carcinoma in rats

All animal experiments were performed in accordance with the institutional guidelines and approved by the Internal Committee for the Care and Use of Laboratory Animals of the Center for Research and Advanced Studies of the National Polytechnic Institute under number 0001–02 and following the guidelines of the Official Mexican Standard NOM-062-ZOO-1999. Male Fischer-344 rats weighting 180 to 200 g, from the Production Unit of Experimental Laboratory Animals (UPEAL-CINVESTAV, Mexico), were feed *ad libitum* and housed in a controlled environment (12 h light/12 h dark cycle; at 22 ± 2°C, and humidity at 55 ± 10%). For carcinogenesis, a modified resistant hepatocyte model was used [10]. Rats were initiated with diethylnitrosamine (DEN) (200 mg/kg of body weight) at day 0. Then, 2-acetylaminofluorene (AAF) was administered (20 mg/kg per dose) at days 7, 8 and 9, followed by 3/5 partial hepatectomy (PH) at day 10 (Fig 1). Three groups of non-treated animals were sacrificed by exsanguination on the first day and at 9 and 18 months after the beginning of the experiment. Treated animals were sacrificed at 1, 7, 11 and 16 days and at 1, 9 and 18 months. Their livers were excised, washed in physiological saline solution, frozen with liquid nitrogen in 2-methyl butane (Sigma-Aldrich) and stored at -80°C.

### Histochemical analysis and tissue selection

Samples from total liver tissue were taken from control and treated rats at days 1, 7, 11 and 16. Based on histochemical analysis, pre-neoplastic nodules, tumoral tissues and their adjacent tissues were identified and excised at 1, 9 and 18 months (Figs 1 and S1). Tissues were selected using a histochemical reaction of gamma-glutamyl transpeptidase (GGT) activity in tissues slides of 20 μm following the protocol described by Rutenburg [59] (Fig 1B). Images of GGT-positive lesions were captured with a digital camera (Color view 12, Soft Imaging System GmbH, Muenster, Germany) and quantified with image analysis software (AnalySIS Soft Imaging System GmbH, version 3.00).

In total we had 11 pairwise contrasts for the differential expression analysis, described as follows. We grouped all evaluated conditions in three categories: i) early stages, including samples obtained in days 1, 7, 11, 16 and from nodules and its adjacent tissues obtained in the first month after treatment initiation. These were compared against rats without treatment sacrificed the first day; ii) nodular, tumor and its adjacent tissue samples obtained from nine months rats were compared against rats without treatment of the same age; and iii) tumor and adjacent tissues samples obtained from eighteen months rats were compared against rats without treatment of the same age (Table 1). For transcriptomic analysis a total of 40 rats were sacrificed, four replicates per stage of HCC development, tissue and controls. For proteomic and metabolic analysis 30 rats were sacrificed, three replicates in each evaluated stage, tissue, and controls (Table 1). Independent rats were used to obtain transcriptomic, proteomic, and metabolic data.

### RNA extraction and microarray hybridization

Once selected and separately collected nodular, tumoral and its adjacent tissues, total RNA was isolated using TriPureIsolation Reagent (Roche) according to the manufacturer’s protocol. The microarray analysis was performed as previously reported [60] using GeneChip Rat Exon 1.0 ST Arrays (Affymetrix, Inc., Santa Clara, CA, USA), which are genome-wide arrays containing over 1 million probe sets, spread across the exons of all the known genes, with >4 perfect match probes each, plus a number of additional regions.

### Differential gene expression analysis in rat HCC

Four replicas for each condition and controls were analyzed. This created the 11 pairwise contrasts for the differential expression analysis as described in *histochemical analysis and tissue selection* section and in Table 1.

Data analysis was performed using the packages *oligo* [61] (version 1.38.0) and *pd*.*raex*.*1*.*0*.*st*.*v1* [62] (version 3.14.1) in R (version 3.3.2). Normalization and probe summarization were performed using the Robust Multichip Average algorithm [63, 64], and differential gene expression analysis was performed using *limma* [65] (version 3.34.6). DEG were selected based on a fold-change >1. The *p* value was adjusted for multiple tests [66] and the cut-off value was set to adjusted *p* value < 0.05.

### Mitochondria isolation and proteomic profiles

Mitochondria were extracted from rat liver samples according to Frezza *et al*. [67], with modifications. Samples of liver tissue in 1.5 ml tubes were macerated in a dry ice bath using a sterile plastic pestle. IBc buffer (0.1 M Tris-MOPS, 0.001 M EGTA-Tris, 0.2 M sucrose, pH 7.4) was added, the tubes were centrifuged at 600 g for 10 minutes at 4°C, and the supernatant was recovered in a new tube. This tube was centrifuged at 7000 g for 10 minutes at 4°C and the supernatant was discarded. This last step was repeated until the supernatant was clear. The remaining pellet contained isolated mitochondria that were further purified by ultracentrifugation in discontinuous sucrose gradients [68].

Mitochondrial proteins were extracted using one volume of extraction buffer (6 M urea, 2 M thiourea, 3% CHAPS) per volume of purified mitochondria. Total proteins were precipitated using trichloroacetic acid and resuspended in water. Protein concentration was determined using Bradford assay (S20 Table). Three replicas of mitochondrial protein extracts from each of the samples and control tissues were sent to the Proteomics Core Facility of the University of California, Davis to be analyzed. Total mitochondrial proteins were digested with trypsin (20:1) and run on an Orbitrap Q-Exactive mass spectrometer (Thermo Scientific) coupled to a Proxeon Easy-nLC II HPLC (Thermo Scientific) using a C18 column of 120 cm for 90 minutes.

A database of 29,998 amino acid sequences, downloaded from Uniprot [12] (http://www.uniprot.org) and constituting all rat proteins was used as reference for protein identification. Protein identification and quantification was done with MaxQuant [69] version 1.5.2.8. For identification, the peptides were of minimum 6 amino acids and had at least 1 unique peptide identified per protein. A false discovery rate (FDR) of 1% at both peptide and protein level was used. Average absolute mass deviation was set to 0.2 parts per million. For quantitation we used intensity based absolute quantification (iBAQ) [70].

### Differential protein expression analysis

Differential expression analysis was based on iBAQ values, normalized by the amount of protein of each sample injected in the LC-MS system (S20 Table). Three replicas for each condition and controls were analyzed against the same age controls as described in *histochemical analysis and tissue selection* section and shown in Table 1. For data analysis we used *DEP* package [71] version 1.0.1 in R. Proteins quantified in the three replicates of at least one of the evaluated conditions were included in the analysis (531). Normalization of data was performed using the *vsn* [72] package in R. Imputation of missing values was done with *MSnbase* package version 2.4.2 [73] using the smallest non-missing value with *MinDet* function. To evaluate the significance of differentially expressed proteins we used the *DEP* package that depends on *limma* [65] (version 3.34.6). DEP were considered as such when the adjusted *p* value ≤ 0.05 and presented a one-fold change in expression level in comparison with the control.

### Metabolic profiling

To extract metabolites, 100 μl of water was added to 100 μg of purified mitochondria along with 0.005 μg/ml of malonic acid as standard. The solution was frozen at -20°C, thawed, mixed in vortex for 2 minutes, and centrifuged at 10,000 g. The supernatant was collected, frozen at -80°C and lyophilized. Samples were derivatized using BSTFA-TMCS (99:1) and pyridine in a 4:1 volume relation at 80°C for 30 minutes. Then, the solution was centrifuged at 10,000 g and 1 μl of the supernatant was injected in a GC-MS system (Agilent GC 7890B, Agilent MS 5977A, available at our institution) according to chromatographic conditions described previously [74]. Quantitation was done with Agilent MassHunter Quantitative Analysis (for GCMS) software version B.07.00/Build 7.0.457.0 (Agilent Technologies, 2008). Area under the intensity curve was used to quantify metabolites using a known concentration curve of standard metabolites, namely citric acid, malic acid, succinic acid, glutamate, glutamine, lactic acid, alpha-ketoglutaric acid and fumaric acid from Sigma-Aldrich (St. Louis, MO, USA). Three replicas for each condition and controls were analyzed against the same age controls as described in *histochemical analysis and tissue selection* section and shown in Table 1. Differential concentration analysis of metabolites was done using Student´s T test in *Perseus* [75, 76] (S12 Table).

### Hierarchical clustering and supervised classification of HCC development stage

Hierarchical clustering for genes and mitochondrial proteins were done using *ComplexHeatmap* [77] in R using only DEG or DEP, respectively. Non-differential genes and proteins were excluded for all posterior analyses. Clustering and heat map visualization were done using a table containing the log2 fold change mean of all replicas for each sample. For columns (HCC development stages) Pearson’s correlation was used, while Euclidean distance was used for rows (DEG or DEP).

We evaluated two models for sample classification according to stage of HCC development using the proteomic profile of each replicate. We separated samples into 3 groups according to stage of HCC development regardless of sampled tissue type. Group 1 included samples collected within the first month, the groups 9 and 18, were samples collected after nine and eighteen months of carcinogenic treatment, respectively. Group 1 had 18 samples; group 9, 9 samples; and group 18, 6 samples. Classification was done using a supporting vector machine algorithm, with a linear kernel, C = 10, considering 363 features ranked using ANOVA and s0 = 0. For cross-validation we performed 250 repeats by random sampling with a test set of 33% and another 250 repeats using ten-fold cross-validation in *Perseus* [75, 76]. Then, the expression profile of the top ranking 16 proteins that lead to classification error rate less than 10% were used to perform a hierarchical clustering using Pearson’s correlation.

### Pathway over-representation-based Integration using Biological level expression (PIB)

First, we classified DEG by stage of HCC development as described above. Then, DEG were separated into two categories, upregulated and downregulated, and were mapped into the pathways from the Kyoto Encyclopedia of Genes and Genomes (KEGG) database [14, 15]. We used upregulated or downregulated mapped genes separately to perform an analysis of pathways over-representation with *clusterProfiler* [78] package (version 3.6.0) using *enrichKEGG* function in R. Pathways were considered statistically significant if the adjusted *p* value was <0.05, and significant pathways were included in the table. For DEP analysis we used an identical pipeline to obtain another table that was combined with the previously obtained DEG table to obtain an “enrichment status” for each pathway. Enrichment status classification was performed according to biological level over-representation significance (RNA, protein, both or none) and expression values of DEG or DEP (downregulation, upregulation or no regulation). This gave us a table containing 11 categories of enrichment status. The table with all classifications is shown in S21 Table.

### Pathway enrichment integration analysis by weighed sum of Z (Stouffer) test

This method combines the *p* values from each independent test into one [79], where each independent test is a pathway over-representation test for each omics data. *P* values were weighted with transcriptomic and mitochondrial proteomics universe size, respectively, to reduce false positives [80, 81]. To perform this analysis the *metap* [82] package was used in R.

### Weighted Gene Co-expression Network Analysis (WGCNA)

WGCNA [83] assesses similarities in expression patterns through correlation, with the assumption that genes with similar expression profiles undergo similar regulation processes and are likely to share common biochemical pathways or cellular functions, and therefore can be grouped into co-expression modules. Then, modules are correlated with other features, i.e. body weight or blood metabolite levels. Here we used *WGCNA* package to find transcriptional modules and correlate them with the profiles of quantified mitochondrial proteins and metabolites as well as miRNAs genes (all these here referred to as molecular features).

We used 137 samples, 56 of transcriptomic, 42 of proteomic and 38 of metabolic data, representing 11 stages and three control groups, as described in *Histochemical analysis and tissue selection* section. First, we imputed missing values in the proteomic dataset of 531 proteins described in *Differential protein expression analysis* section. Missing values were imputed considering its nature of origin, missing at random (MAR) or missing not at random (MNAR), according to Lazar *et al*. [84] (S22 Table) using the package *ImputeLCMD* [85]. MNAR values were imputed using random draws from a Gaussian distribution centered in the minimal value detected using the function *impute*.*MinProb* (q = 0.1, tune.sigma = 1), while MAR data was imputed using k nearest neighbor algorithm, using k = 11 [84, 85].

For WGCNA, gene expression profiles were correlated with each other using biweigthed midcorrelation. Correlation coefficients were transformed into adjacency and Topological Overlap Matrix (TOM) similarity matrices, based on the soft-threshold power (β) of 24 that was chosen using *pickSoftThreshold* function [83], because it was the smallest threshold that resulted in a scale-free R^2^ fit >0.85. Genes were clustered into modules with similar expression profiles by using hierarchical clustering based on the TOM dissimilarity (1-TOM) using *flashClust* function. Signed module identification was done using *cutreeDynamic* function with a minimum module size of 20. The resulting 9 modules of co-expressed genes were used to calculate module eigengenes (MEs; or the first principal component of the module). Finally, MEs, were correlated with the molecular features, according to their expression patterns. Since hundreds of molecular features were quantified in our experiment and correlated with transcriptomic modules, we filtered out all features with no significant correlation (*p* ≤ 0.001) with at least one transcriptional module.

### Transcription factor binding site enrichment analysis

Genes within each co-expressed transcriptomic module were compared to the rest of the rat genome in order to identify over-represented TF binding sites using the online tool ShinyGO [86] v0.61 (http://bioinformatics.sdstate.edu/go/).

### Transcription factors interaction network of royalblue module

TF interaction network was performed in GeneMANIA [87, 88] (https://genemania.org/) with TF present in each module and TF with over-represented binding sites in the modules were used as input.

## Supporting information

S1 Fig GGT staining in samples from different stages of HCC development. (TIF)

S2 Fig Differentially expressed genes and proteins throughout HCC development. (TIF)

S3 Fig Royalblue module transcription factors GeneMANIA network results. (TIF)

S4 Fig Transcriptomic modules-molecular features correlation and p values. (PDF)

S1 Table Gene expression differential analysis in HCC. (XLSX)

S2 Table Number of differentially expressed genes in HCC development. (XLSX)

S3 Table iBAQ values of each sample in HCC development. (XLSX)

S4 Table Number of identified proteins in HCC development. (XLSX)

S5 Table Number of differentially expressed proteins in HCC development. (XLSX)

S6 Table HCC mitochondrial protein expression fold changes and p values. (XLSX)

S7 Table DEP used in classification model. (XLSX)

S8 Table Predicted stage of HCC development groups according to our svm model using proteomic profiles. (XLSX)

S9 Table Percentage of error in classification of svm model. (XLSX)

S10 Table KEGG based over-representation pathway analysis of differentially expressed genes. (XLSX)

S11 Table KEGG based over-representation pathway analysis of differentially expressed proteins. (XLSX)

S12 Table Metabolic profiles and metabolic differential analysis in HCC development. (XLSX)

S13 Table Pathway over-representation-based Integration using Biological level expression. (XLSX)

S14 Table Integration based in pathway Stouffer_s Z. (XLSX)

S15 Table Transcriptomic gene expression and modules identification in WGCNA. (XLSX)

S16 Table KEGG over-representation pathways analysis of transcriptional modules. (XLSX)

S17 Table TF binding motif enrichment analysis of transcriptomic modules. (XLSX)

S18 Table DEP, miRNAs and metabolites log2 expression for WGCNA based integration. (XLSX)

S19 Table Module-molecular feature correlations and p values. (XLSX)

S20 Table Mitochondrial total protein obtained from each evaluated condition. (XLSX)

S21 Table Over-representation status and number code used in PIB. (XLSX)

S22 Table Nature of origin of missing values in mitochondrial proteomic data. (XLSX)
