# Supplementary figures and images for: Integration of chronological omics data reveals mitochondrial regulatory mechanisms during the development of hepatocellular carcinoma
Source: PLoS One. 2021 Aug 12;16(8):e0256016. doi: 10.1371/journal.pone.0256016 (PMC8360386; doi:10.1371/journal.pone.0256016)

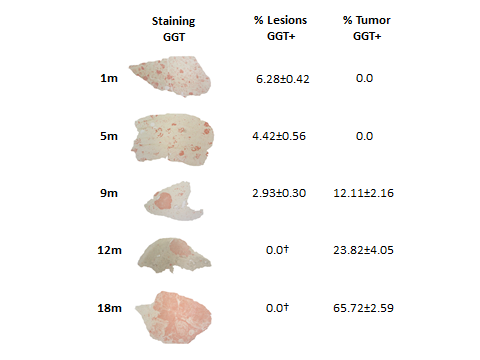

Supplement: S1 Fig — (TIF) [file pone.0256016.s001.tif]

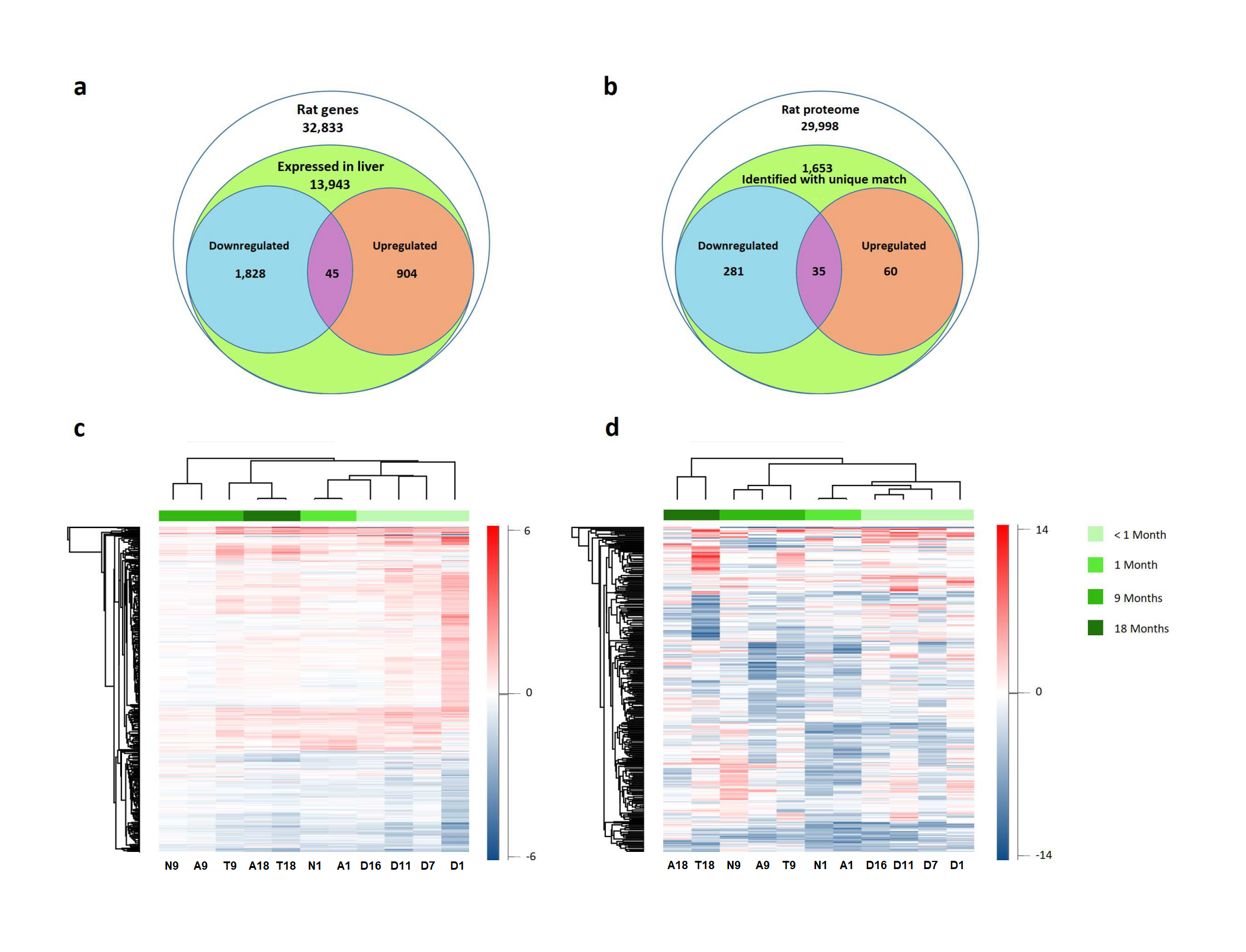

Supplement: S2 Fig — (TIF) [file pone.0256016.s002.tif]

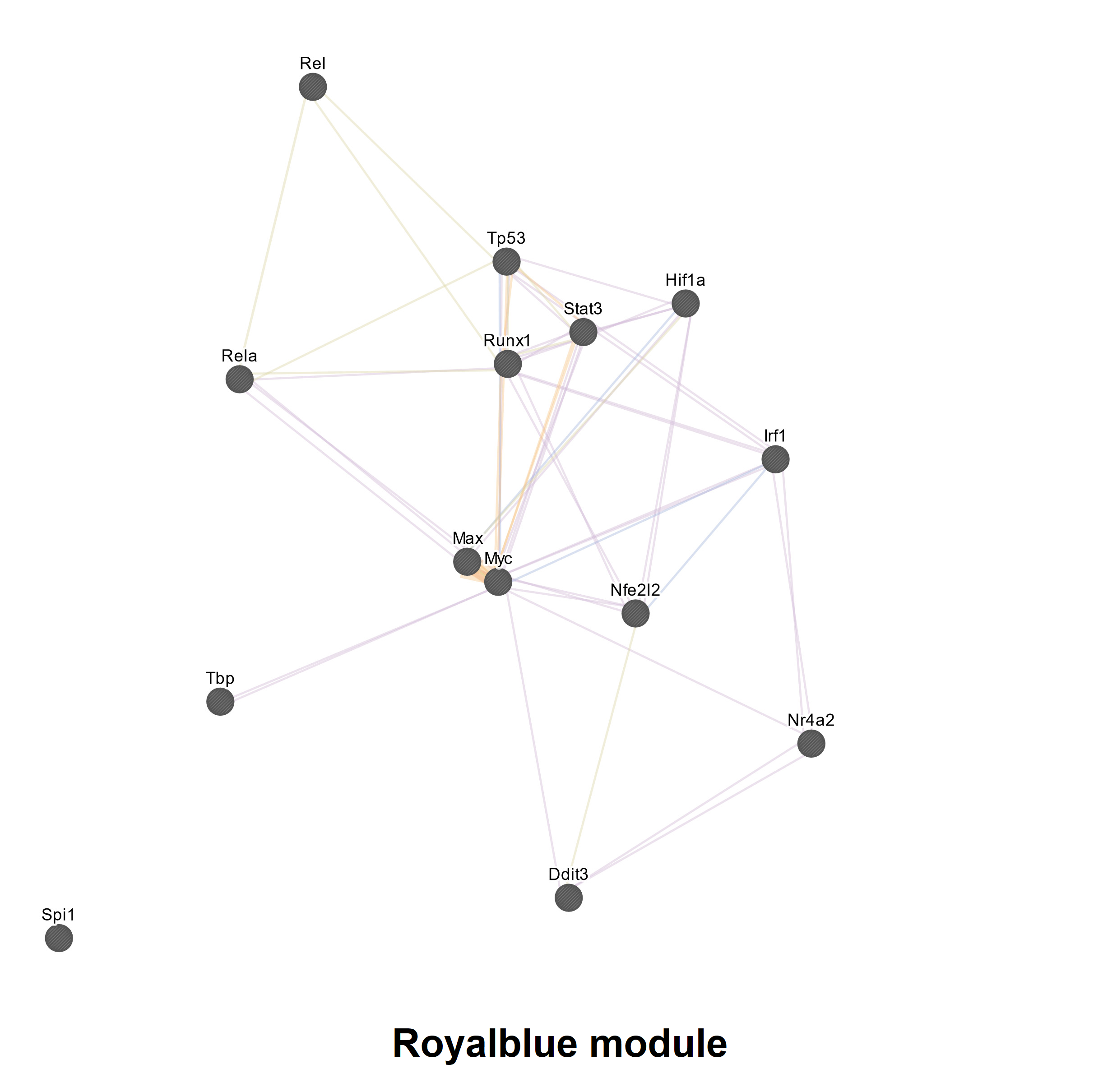

Supplement: S3 Fig — (TIF) [file pone.0256016.s003.tif]
