# Supplementary material for: Integration of chronological omics data reveals mitochondrial regulatory mechanisms during the development of hepatocellular carcinoma
Source: PLoS One. 2021 Aug 12;16(8):e0256016. doi: 10.1371/journal.pone.0256016 (PMC8360386; doi:10.1371/journal.pone.0256016)
Supplement: S4 Fig — (PDF) [file pone.0256016.s004.pdf]

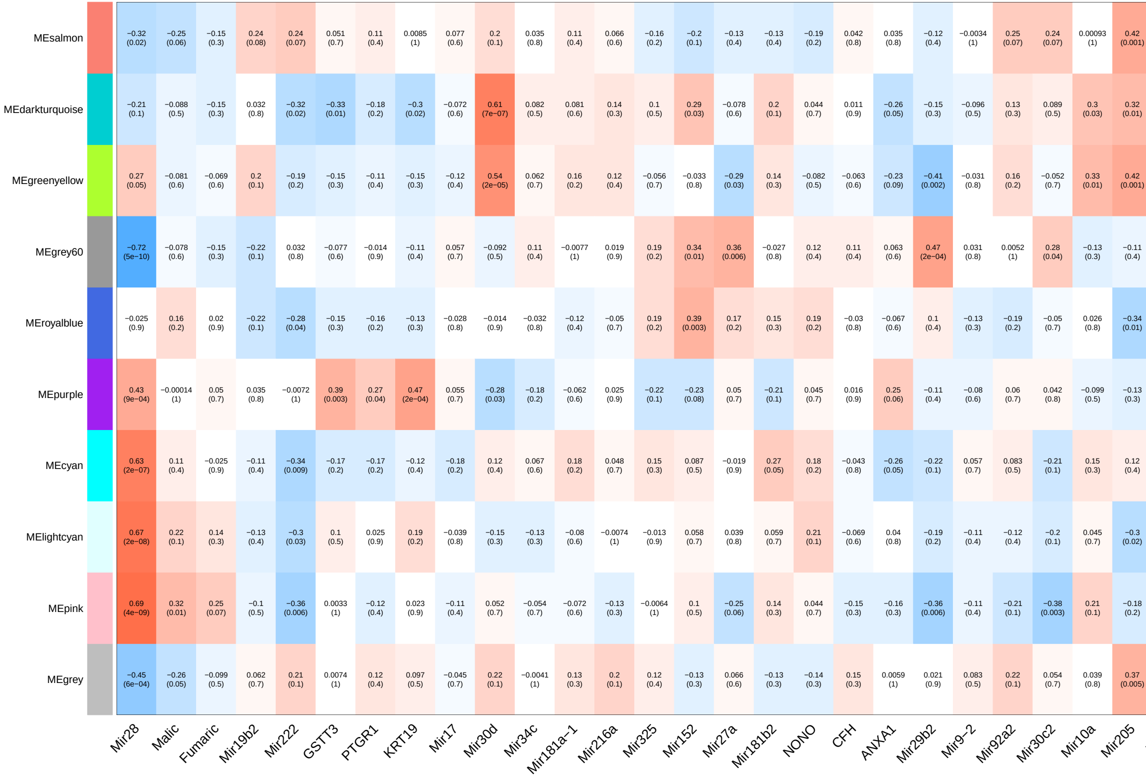
MEsalmon
MEdarkturquoise
MEgreenyellow
MEgrey60
MEroyalblue
MEpurple
MEcyan
MElightcyan
MEpink
MEgrey
Mir28
Malic
Fumaric
Mir19b2
Mir222
GSTT3
PTGR1
KRT19
Mir17
Mir30d
Mir34c
Mir181a−1
Mir216a
Mir325
Mir152
Mir27a
Mir181b2
NONO
CFH
ANXA1
Mir29b2
Mir9−2
Mir92a2
Mir30c2
Mir10a
Mir205
−0.32 (0.02) −0.25 (0.06) −0.15 (0.3) 0.24 (0.08) 0.24 (0.07) 0.051 (0.7) 0.11 (0.4) 0.0085 (1) 0.077 (0.6) 0.2 (0.1) 0.035 (0.8) 0.11 (0.4) 0.066 (0.6) −0.16 (0.2) −0.2 (0.1) −0.13 (0.4) −0.13 (0.4) −0.19 (0.2) 0.042 (0.8) 0.035 (0.8) −0.12 (0.4) −0.0034 (1) 0.25 (0.07) 0.24 (0.07) 0.00093 (1) 0.42 (0.001)
−0.21 (0.1) −0.088 (0.5) −0.15 (0.3) 0.032 (0.8) −0.32 (0.02) −0.33 (0.01) −0.18 (0.2) −0.3 (0.02) −0.072 (0.6) 0.61 (7e−07) 0.082 (0.5) 0.081 (0.6) 0.14 (0.3) 0.1 (0.5) 0.29 (0.03) −0.078 (0.6) 0.2 (0.1) 0.044 (0.7) 0.011 (0.9) −0.26 (0.05) −0.15 (0.3) −0.096 (0.5) 0.13 (0.3) 0.089 (0.5) 0.3 (0.03) 0.32 (0.01)
0.27 (0.05) −0.081 (0.6) −0.069 (0.6) 0.2 (0.1) −0.19 (0.2) −0.15 (0.3) −0.11 (0.4) −0.15 (0.3) −0.12 (0.4) 0.54 (2e−05) 0.062 (0.7) 0.16 (0.2) 0.12 (0.4) −0.056 (0.7) −0.033 (0.8) −0.29 (0.03) 0.14 (0.3) −0.082 (0.5) −0.063 (0.6) −0.23 (0.09) −0.41 (0.002) −0.031 (0.8) 0.16 (0.2) −0.052 (0.7) 0.33 (0.01) 0.42 (0.001)
−0.72 (5e−10) −0.078 (0.6) −0.15 (0.3) −0.22 (0.1) 0.032 (0.8) −0.077 (0.6) −0.014 (0.9) −0.11 (0.4) 0.057 (0.7) −0.092 (0.5) 0.11 (0.4) −0.0077 (1) 0.019 (0.9) 0.19 (0.2) 0.34 (0.01) 0.36 (0.006) −0.027 (0.8) 0.12 (0.4) 0.11 (0.4) 0.063 (0.6) 0.47 (2e−04) 0.031 (0.8) 0.0052 (1) 0.28 (0.04) −0.13 (0.3) −0.11 (0.4)
−0.025 (0.9) 0.16 (0.2) 0.02 (0.9) −0.22 (0.1) −0.28 (0.04) −0.15 (0.3) −0.16 (0.2) −0.13 (0.3) −0.028 (0.8) −0.014 (0.9) −0.032 (0.8) −0.12 (0.4) −0.05 (0.7) 0.19 (0.2) 0.39 (0.003) 0.17 (0.2) 0.15 (0.3) 0.19 (0.2) −0.03 (0.8) −0.067 (0.6) 0.1 (0.4) −0.13 (0.3) −0.19 (0.2) −0.05 (0.7) 0.026 (0.8) −0.34 (0.01)
0.43 (9e−04) −0.00014 (1) 0.05 (0.7) 0.035 (0.8) −0.0072 (1) 0.39 (0.003) 0.27 (0.04) 0.47 (2e−04) 0.055 (0.7) −0.28 (0.03) −0.18 (0.2) −0.062 (0.6) 0.025 (0.9) −0.22 (0.1) −0.23 (0.08) 0.05 (0.7) −0.21 (0.1) 0.045 (0.7) 0.016 (0.9) 0.25 (0.06) −0.11 (0.4) −0.08 (0.6) 0.06 (0.7) 0.042 (0.8) −0.099 (0.5) −0.13 (0.3)
0.63 (2e−07) 0.11 (0.4) −0.025 (0.9) −0.11 (0.4) −0.34 (0.009) −0.17 (0.2) −0.17 (0.2) −0.12 (0.4) −0.18 (0.2) 0.12 (0.4) 0.067 (0.6) 0.18 (0.2) 0.048 (0.7) 0.15 (0.3) 0.087 (0.5) −0.019 (0.9) 0.27 (0.05) 0.18 (0.2) −0.043 (0.8) −0.26 (0.05) −0.22 (0.1) 0.057 (0.7) 0.083 (0.5) −0.21 (0.1) 0.15 (0.3) 0.12 (0.4)
0.67 (2e−08) 0.22 (0.1) 0.14 (0.3) −0.13 (0.4) −0.3 (0.03) 0.1 (0.5) 0.025 (0.9) 0.19 (0.2) −0.039 (0.8) −0.15 (0.3) −0.13 (0.3) −0.08 (0.6) −0.0074 (1) −0.013 (0.9) 0.058 (0.7) 0.039 (0.8) 0.059 (0.7) 0.21 (0.1) −0.069 (0.6) 0.04 (0.8) −0.19 (0.2) −0.11 (0.4) −0.12 (0.4) −0.2 (0.1) 0.045 (0.7) −0.3 (0.02)
0.69 (4e−09) 0.32 (0.01) 0.25 (0.07) −0.1 (0.5) −0.36 (0.006) 0.0033 (1) −0.12 (0.4) 0.023 (0.9) −0.11 (0.4) 0.052 (0.7) −0.054 (0.7) −0.072 (0.6) −0.13 (0.3) −0.0064 (1) 0.1 (0.5) −0.25 (0.06) 0.14 (0.3) 0.044 (0.7) −0.15 (0.3) −0.16 (0.3) −0.36 (0.006) −0.11 (0.4) −0.21 (0.1) −0.38 (0.003) 0.21 (0.1) −0.18 (0.2)
−0.45 (6e−04) −0.26 (0.05) −0.099 (0.5) 0.062 (0.7) 0.21 (0.1) 0.0074 (1) 0.12 (0.4) 0.097 (0.5) −0.045 (0.7) 0.22 (0.1) −0.0041 (1) 0.13 (0.3) 0.2 (0.1) 0.12 (0.4) −0.13 (0.3) 0.066 (0.6) −0.13 (0.3) −0.14 (0.3) 0.15 (0.3) 0.0059 (1) 0.021 (0.9) 0.083 (0.5) 0.22 (0.1) 0.054 (0.7) 0.039 (0.8) 0.37 (0.005)

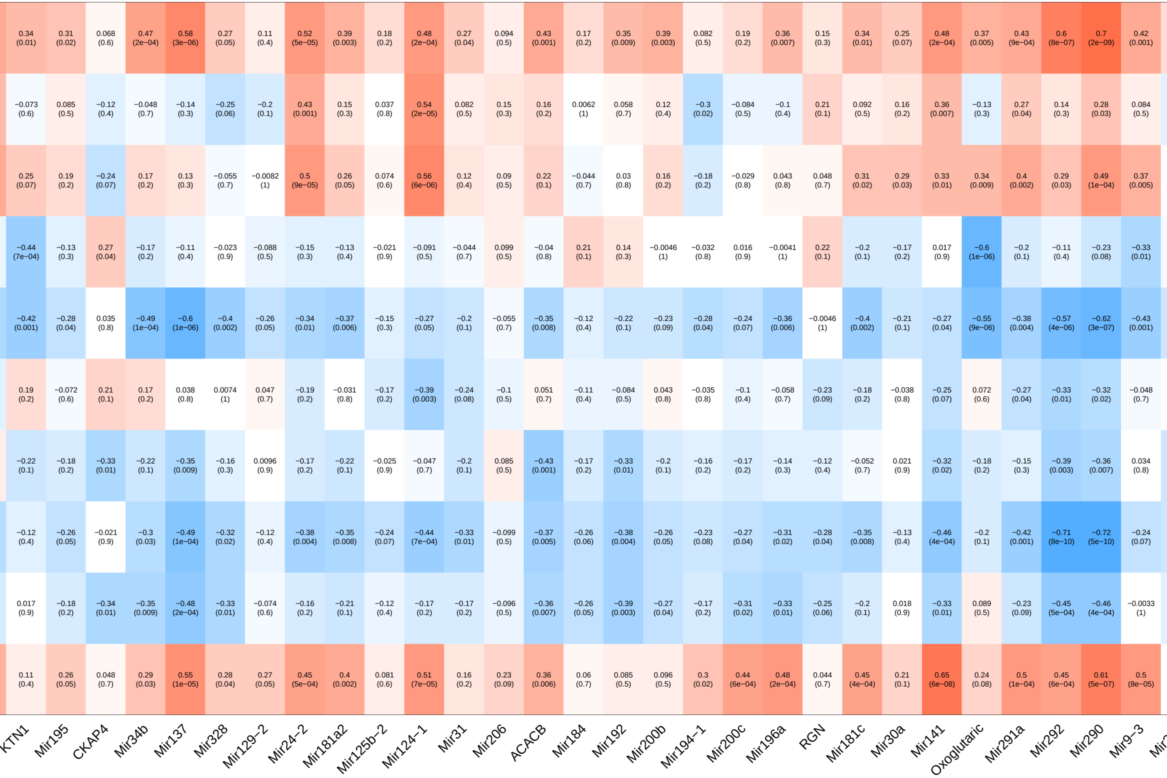

0.34 (0.01) 0.31 (0.02) 0.068 (0.6) 0.47 (2e−04) 0.58 (3e−06) 0.27 (0.05) 0.11 (0.4) 0.52 (5e−05) 0.39 (0.003) 0.18 (0.2) 0.48 (2e−04) 0.27 (0.04) 0.094 (0.5) 0.43 (0.001) 0.17 (0.2) 0.35 (0.009) 0.39 (0.003) 0.082 (0.5) 0.19 (0.2) 0.36 (0.007) 0.15 (0.3) 0.34 (0.01) 0.25 (0.07) 0.48 (2e−04) 0.37 (0.005) 0.43 (9e−04) 0.6 (8e−07) 0.7 (2e−09) 0.42 (0.001)
−0.073 (0.6) 0.085 (0.5) −0.12 (0.4) −0.048 (0.7) −0.14 (0.3) −0.25 (0.06) −0.2 (0.1) 0.43 (0.001) 0.15 (0.3) 0.037 (0.8) 0.54 (2e−05) 0.082 (0.5) 0.15 (0.3) 0.16 (0.2) 0.0062 (1) 0.058 (0.7) 0.12 (0.4) −0.3 (0.02) −0.084 (0.5) −0.1 (0.4) 0.21 (0.1) 0.092 (0.5) 0.16 (0.2) 0.36 (0.007) −0.13 (0.3) 0.27 (0.04) 0.14 (0.3) 0.28 (0.03) 0.084 (0.5)
0.25 (0.07) 0.19 (0.2) −0.24 (0.07) 0.17 (0.2) 0.13 (0.3) −0.055 (0.7) −0.0082 (1) 0.5 (9e−05) 0.26 (0.05) 0.074 (0.6) 0.56 (6e−06) 0.12 (0.4) 0.09 (0.5) 0.22 (0.1) −0.044 (0.7) 0.03 (0.8) 0.16 (0.2) −0.18 (0.2) −0.029 (0.8) 0.043 (0.8) 0.048 (0.7) 0.31 (0.02) 0.29 (0.03) 0.33 (0.01) 0.34 (0.009) 0.4 (0.002) 0.29 (0.03) 0.49 (1e−04) 0.37 (0.005)
−0.44 (7e−04) −0.13 (0.3) 0.27 (0.04) −0.17 (0.2) −0.11 (0.4) −0.023 (0.9) −0.088 (0.5) −0.15 (0.3) −0.13 (0.4) −0.021 (0.9) −0.091 (0.5) −0.044 (0.7) 0.099 (0.5) −0.04 (0.8) 0.21 (0.1) 0.14 (0.3) −0.0046 (1) −0.032 (0.8) 0.016 (0.9) −0.0041 (1) 0.22 (0.1) −0.2 (0.1) −0.17 (0.2) 0.017 (0.9) −0.6 (1e−06) −0.2 (0.1) −0.11 (0.4) −0.23 (0.08) −0.33 (0.01)
−0.42 (0.001) −0.28 (0.04) 0.035 (0.8) −0.49 (1e−04) −0.6 (1e−06) −0.4 (0.002) −0.26 (0.05) −0.34 (0.01) −0.37 (0.006) −0.15 (0.3) −0.27 (0.05) −0.2 (0.1) −0.055 (0.7) −0.35 (0.008) −0.12 (0.4) −0.22 (0.1) −0.23 (0.09) −0.28 (0.04) −0.24 (0.07) −0.36 (0.006) −0.0046 (1) −0.4 (0.002) −0.21 (0.1) −0.27 (0.04) −0.55 (9e−06) −0.38 (0.004) −0.57 (4e−06) −0.62 (3e−07) −0.43 (0.001)
0.19 (0.2) −0.072 (0.6) 0.21 (0.1) 0.17 (0.2) 0.038 (0.8) 0.0074 (1) 0.047 (0.7) −0.19 (0.2) −0.031 (0.8) −0.17 (0.2) −0.39 (0.003) −0.24 (0.08) −0.1 (0.5) 0.051 (0.7) −0.11 (0.4) −0.084 (0.5) 0.043 (0.8) −0.035 (0.8) −0.1 (0.4) −0.058 (0.7) −0.23 (0.09) −0.18 (0.2) −0.038 (0.8) −0.25 (0.07) 0.072 (0.6) −0.27 (0.04) −0.33 (0.01) −0.32 (0.02) −0.048 (0.7)
−0.22 (0.1) −0.18 (0.2) −0.33 (0.01) −0.22 (0.1) −0.35 (0.009) −0.16 (0.3) 0.0096 (0.9) −0.17 (0.2) −0.22 (0.1) −0.025 (0.9) −0.047 (0.7) −0.2 (0.1) 0.085 (0.5) −0.43 (0.001) −0.17 (0.2) −0.33 (0.01) −0.2 (0.1) −0.16 (0.2) −0.17 (0.2) −0.14 (0.3) −0.12 (0.4) −0.052 (0.7) 0.021 (0.9) −0.32 (0.02) −0.18 (0.2) −0.15 (0.3) −0.39 (0.003) −0.36 (0.007) 0.034 (0.8)
−0.12 (0.4) −0.26 (0.05) −0.021 (0.9) −0.3 (0.03) −0.49 (1e−04) −0.32 (0.02) −0.12 (0.4) −0.38 (0.004) −0.35 (0.008) −0.24 (0.07) −0.44 (7e−04) −0.33 (0.01) −0.099 (0.5) −0.37 (0.005) −0.26 (0.06) −0.38 (0.004) −0.26 (0.05) −0.23 (0.08) −0.27 (0.04) −0.31 (0.02) −0.28 (0.04) −0.35 (0.008) −0.13 (0.4) −0.46 (4e−04) −0.2 (0.1) −0.42 (0.001) −0.71 (8e−10) −0.72 (5e−10) −0.24 (0.07)
0.017 (0.9) −0.18 (0.2) −0.34 (0.01) −0.35 (0.009) −0.48 (2e−04) −0.33 (0.01) −0.074 (0.6) −0.16 (0.2) −0.21 (0.1) −0.12 (0.4) −0.17 (0.2) −0.17 (0.2) −0.096 (0.5) −0.36 (0.007) −0.26 (0.05) −0.39 (0.003) −0.27 (0.04) −0.17 (0.2) −0.31 (0.02) −0.33 (0.01) −0.25 (0.06) −0.2 (0.1) 0.018 (0.9) −0.33 (0.01) 0.089 (0.5) −0.23 (0.09) −0.45 (5e−04) −0.46 (4e−04) −0.0033 (1)
0.11 (0.4) 0.26 (0.05) 0.048 (0.7) 0.29 (0.03) 0.55 (1e−05) 0.28 (0.04) 0.27 (0.05) 0.45 (5e−04) 0.4 (0.002) 0.081 (0.6) 0.51 (7e−05) 0.16 (0.2) 0.23 (0.09) 0.36 (0.006) 0.06 (0.7) 0.085 (0.5) 0.096 (0.5) 0.3 (0.02) 0.44 (6e−04) 0.48 (2e−04) 0.044 (0.7) 0.45 (4e−04) 0.21 (0.1) 0.65 (6e−08) 0.24 (0.08) 0.5 (1e−04) 0.45 (6e−04) 0.61 (5e−07) 0.5 (8e−05)
KTN1
Mir195
CKAP4
Mir34b
Mir137
Mir328
Mir129−2
Mir24−2
Mir181a2
Mir125b−2
Mir124−1
Mir31
Mir206
ACACB
Mir184
Mir192
Mir200b
Mir194−1
Mir200c
Mir196a
RGN
Mir181c
Mir30a
Mir141
Oxoglutaric
Mir291a
Mir292
Mir290
Mir9−3

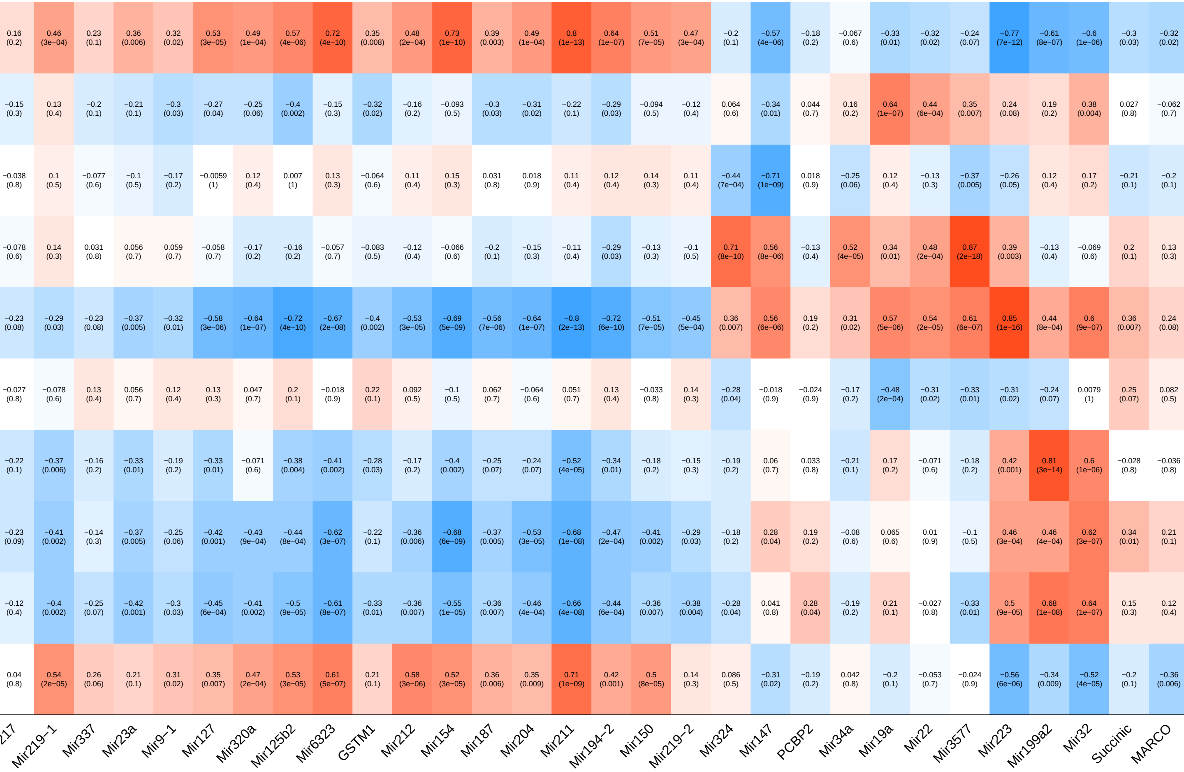

0.16 (0.2) | 0.46 (3e−04) | 0.23 (0.1) | 0.36 (0.006) | 0.32 (0.02) | 0.53 (3e−05) | 0.49 (1e−04) | 0.57 (4e−06) | 0.72 (4e−10) | 0.35 (0.008) | 0.48 (2e−04) | 0.73 (1e−10) | 0.39 (0.003) | 0.49 (1e−04) | 0.8 (1e−13) | 0.64 (1e−07) | 0.51 (7e−05) | 0.47 (3e−04) | −0.2 (0.1) | −0.57 (4e−06) | −0.18 (0.2) | −0.067 (0.6) | −0.33 (0.01) | −0.32 (0.02) | −0.24 (0.07) | −0.77 (7e−12) | −0.61 (8e−07) | −0.6 (1e−06) | −0.3 (0.03) | −0.32 (0.02)
−0.15 (0.3) | 0.13 (0.4) | −0.2 (0.1) | −0.21 (0.1) | −0.3 (0.03) | −0.27 (0.04) | −0.25 (0.06) | −0.4 (0.002) | −0.15 (0.3) | −0.32 (0.02) | −0.16 (0.2) | −0.093 (0.5) | −0.3 (0.03) | −0.31 (0.02) | −0.22 (0.1) | −0.29 (0.03) | −0.094 (0.5) | −0.12 (0.4) | 0.064 (0.6) | −0.34 (0.01) | 0.044 (0.7) | 0.16 (0.2) | 0.64 (1e−07) | 0.44 (6e−04) | 0.35 (0.007) | 0.24 (0.08) | 0.19 (0.2) | 0.38 (0.004) | 0.027 (0.8) | −0.062 (0.7)
−0.038 (0.8) | 0.1 (0.5) | −0.077 (0.6) | −0.1 (0.5) | −0.17 (0.2) | −0.0059 (1) | 0.12 (0.4) | 0.007 (1) | 0.13 (0.3) | −0.064 (0.6) | 0.11 (0.4) | 0.15 (0.3) | 0.031 (0.8) | 0.018 (0.9) | 0.11 (0.4) | 0.12 (0.4) | 0.14 (0.3) | 0.11 (0.4) | −0.44 (7e−04) | −0.71 (1e−09) | 0.018 (0.9) | −0.25 (0.06) | 0.12 (0.4) | −0.13 (0.3) | −0.37 (0.005) | −0.26 (0.05) | 0.12 (0.4) | 0.17 (0.2) | −0.21 (0.1) | −0.2 (0.1)
−0.078 (0.6) | 0.14 (0.3) | 0.031 (0.8) | 0.056 (0.7) | 0.059 (0.7) | −0.058 (0.7) | −0.17 (0.2) | −0.16 (0.2) | −0.057 (0.7) | −0.083 (0.5) | −0.12 (0.4) | −0.066 (0.6) | −0.2 (0.1) | −0.15 (0.3) | −0.11 (0.4) | −0.29 (0.03) | −0.13 (0.3) | −0.1 (0.5) | 0.71 (8e−10) | 0.56 (8e−06) | −0.13 (0.4) | 0.52 (4e−05) | 0.34 (0.01) | 0.48 (2e−04) | 0.87 (2e−18) | 0.39 (0.003) | −0.13 (0.4) | −0.069 (0.6) | 0.2 (0.1) | 0.13 (0.3)
−0.23 (0.08) | −0.29 (0.03) | −0.23 (0.08) | −0.37 (0.005) | −0.32 (0.01) | −0.58 (3e−06) | −0.64 (1e−07) | −0.72 (4e−10) | −0.67 (2e−08) | −0.4 (0.002) | −0.53 (3e−05) | −0.69 (5e−09) | −0.56 (7e−06) | −0.64 (1e−07) | −0.8 (2e−13) | −0.72 (6e−10) | −0.51 (7e−05) | −0.45 (5e−04) | 0.36 (0.007) | 0.56 (6e−06) | 0.19 (0.2) | 0.31 (0.02) | 0.57 (5e−06) | 0.54 (2e−05) | 0.61 (6e−07) | 0.85 (1e−16) | 0.44 (8e−04) | 0.6 (9e−07) | 0.36 (0.007) | 0.24 (0.08)
−0.027 (0.8) | −0.078 (0.6) | 0.13 (0.4) | 0.056 (0.7) | 0.12 (0.4) | 0.13 (0.3) | 0.047 (0.7) | 0.2 (0.1) | −0.018 (0.9) | 0.22 (0.1) | 0.092 (0.5) | −0.1 (0.5) | 0.062 (0.7) | −0.064 (0.6) | 0.051 (0.7) | 0.13 (0.4) | −0.033 (0.8) | 0.14 (0.3) | −0.28 (0.04) | −0.018 (0.9) | −0.024 (0.9) | −0.17 (0.2) | −0.48 (2e−04) | −0.31 (0.02) | −0.33 (0.01) | −0.31 (0.02) | −0.24 (0.07) | 0.0079 (1) | 0.25 (0.07) | 0.082 (0.5)
−0.22 (0.1) | −0.37 (0.006) | −0.16 (0.2) | −0.33 (0.01) | −0.19 (0.2) | −0.33 (0.01) | −0.071 (0.6) | −0.38 (0.004) | −0.41 (0.002) | −0.28 (0.03) | −0.17 (0.2) | −0.4 (0.002) | −0.25 (0.07) | −0.24 (0.07) | −0.52 (4e−05) | −0.34 (0.01) | −0.18 (0.2) | −0.15 (0.3) | −0.19 (0.2) | 0.06 (0.7) | 0.033 (0.8) | −0.21 (0.1) | 0.17 (0.2) | −0.071 (0.6) | −0.18 (0.2) | 0.42 (0.001) | 0.81 (3e−14) | 0.6 (1e−06) | −0.028 (0.8) | −0.036 (0.8)
−0.23 (0.09) | −0.41 (0.002) | −0.14 (0.3) | −0.37 (0.005) | −0.25 (0.06) | −0.42 (0.001) | −0.43 (9e−04) | −0.44 (8e−04) | −0.62 (3e−07) | −0.22 (0.1) | −0.36 (0.006) | −0.68 (6e−09) | −0.37 (0.005) | −0.53 (3e−05) | −0.68 (1e−08) | −0.47 (2e−04) | −0.41 (0.002) | −0.29 (0.03) | −0.18 (0.2) | 0.28 (0.04) | 0.19 (0.2) | −0.08 (0.6) | 0.065 (0.6) | 0.01 (0.9) | −0.1 (0.5) | 0.46 (3e−04) | 0.46 (4e−04) | 0.62 (3e−07) | 0.34 (0.01) | 0.21 (0.1)
−0.12 (0.4) | −0.4 (0.002) | −0.25 (0.07) | −0.42 (0.001) | −0.3 (0.03) | −0.45 (6e−04) | −0.41 (0.002) | −0.5 (9e−05) | −0.61 (8e−07) | −0.33 (0.01) | −0.36 (0.007) | −0.55 (1e−05) | −0.36 (0.007) | −0.46 (4e−04) | −0.66 (4e−08) | −0.44 (6e−04) | −0.36 (0.007) | −0.38 (0.004) | −0.28 (0.04) | 0.041 (0.8) | 0.28 (0.04) | −0.19 (0.2) | 0.21 (0.1) | −0.027 (0.8) | −0.33 (0.01) | 0.5 (9e−05) | 0.68 (1e−08) | 0.64 (1e−07) | 0.15 (0.3) | 0.12 (0.4)
0.04 (0.8) | 0.54 (2e−05) | 0.26 (0.06) | 0.21 (0.1) | 0.31 (0.02) | 0.35 (0.007) | 0.47 (2e−04) | 0.53 (3e−05) | 0.61 (5e−07) | 0.21 (0.1) | 0.58 (3e−06) | 0.52 (3e−05) | 0.36 (0.006) | 0.35 (0.009) | 0.71 (1e−09) | 0.42 (0.001) | 0.5 (8e−05) | 0.14 (0.3) | 0.086 (0.5) | −0.31 (0.02) | −0.19 (0.2) | 0.042 (0.8) | −0.2 (0.1) | −0.053 (0.7) | −0.024 (0.9) | −0.56 (6e−06) | −0.34 (0.009) | −0.52 (4e−05) | −0.2 (0.1) | −0.36 (0.006)
Mir219−1
Mir337
Mir23a
Mir9−1
Mir127
Mir320a
Mir125b2
Mir6323
GSTM1
Mir212
Mir154
Mir187
Mir204
Mir211
Mir194−2
Mir150
Mir219−2
Mir324
Mir147
PCBP2
Mir34a
Mir19a
Mir22
Mir3577
Mir223
Mir199a2
Mir32
Succinic
MARCO

ATL2	UGT1A6	SERPINH1	DDX5	GLG1	RTN4	LDHB	OSTC	Mir19b1	Mir18a	Mir135b	SEC31A	DNAJC3	EIF4A2	MATR3	NDUFB1	SNX3	Mirlet7c−1	CGN	Mir92a1	F1LU71	Mir20a	NDUFS4	SLC16A1	LMNB2	COL6A2	BGN	MTDH	TKFC
0.0086 (0.9)	−0.03 (0.8)	−0.16 (0.2)	−0.29 (0.03)	0.21 (0.1)	0.048 (0.7)	0.32 (0.02)	0.27 (0.05)	0.039 (0.8)	0.29 (0.03)	0.37 (0.005)	0.15 (0.3)	−0.31 (0.02)	−0.14 (0.3)	−0.00042 (1)	0.038 (0.8)	−0.28 (0.04)	0.12 (0.4)	0.011 (0.9)	0.12 (0.4)	0.057 (0.7)	0.0055 (1)	−0.093 (0.5)	−0.12 (0.4)	−0.11 (0.4)	−0.2 (0.1)	−0.23 (0.09)	−0.047 (0.7)	0.28 (0.04)
0.34 (0.01)	−0.23 (0.08)	−0.4 (0.002)	−0.17 (0.2)	−0.082 (0.5)	−0.39 (0.003)	−0.16 (0.2)	−0.21 (0.1)	−0.025 (0.9)	0.13 (0.3)	0.053 (0.7)	0.28 (0.03)	−0.078 (0.6)	0.076 (0.6)	0.085 (0.5)	0.32 (0.01)	0.12 (0.4)	0.59 (1e−06)	0.31 (0.02)	0.39 (0.003)	−0.25 (0.06)	0.35 (0.008)	0.06 (0.7)	0.29 (0.03)	−0.22 (0.1)	−0.084 (0.5)	0.13 (0.3)	0.29 (0.03)	0.23 (0.09)
0.16 (0.2)	−0.17 (0.2)	−0.25 (0.06)	−0.27 (0.05)	−0.0089 (0.9)	−0.13 (0.3)	−0.074 (0.6)	−0.08 (0.6)	−0.19 (0.2)	0.16 (0.2)	0.13 (0.3)	0.25 (0.06)	−0.23 (0.08)	−0.071 (0.6)	−0.054 (0.7)	0.16 (0.2)	−0.092 (0.5)	0.5 (8e−05)	0.006 (1)	0.12 (0.4)	−0.18 (0.2)	0.00082 (1)	−0.081 (0.6)	−0.037 (0.8)	−0.28 (0.03)	−0.32 (0.01)	−0.082 (0.6)	−0.019 (0.9)	0.14 (0.3)
0.054 (0.7)	−0.0024 (1)	−0.074 (0.6)	0.032 (0.8)	0.0041 (1)	−0.24 (0.08)	−0.055 (0.7)	−0.041 (0.8)	0.28 (0.04)	−0.016 (0.9)	−0.02 (0.9)	−0.12 (0.4)	0.22 (0.1)	0.2 (0.1)	0.01 (0.9)	−0.0088 (0.9)	0.063 (0.6)	−0.12 (0.4)	0.35 (0.008)	0.23 (0.08)	0.037 (0.8)	0.37 (0.005)	0.079 (0.6)	0.29 (0.03)	0.098 (0.5)	0.26 (0.05)	0.11 (0.4)	0.2 (0.1)	0.1 (0.5)
0.17 (0.2)	0.029 (0.8)	0.0012 (1)	0.19 (0.2)	−0.23 (0.08)	−0.23 (0.08)	−0.32 (0.02)	−0.33 (0.01)	0.019 (0.9)	−0.23 (0.08)	−0.3 (0.02)	−0.049 (0.7)	0.3 (0.03)	0.16 (0.2)	0.088 (0.5)	0.12 (0.4)	0.3 (0.03)	−0.0095 (0.9)	0.14 (0.3)	0.048 (0.7)	−0.14 (0.3)	0.23 (0.09)	0.18 (0.2)	0.31 (0.02)	0.094 (0.5)	0.23 (0.09)	0.3 (0.03)	0.22 (0.1)	−0.1 (0.5)
−0.16 (0.2)	0.34 (0.009)	0.27 (0.04)	−0.042 (0.8)	0.15 (0.3)	0.32 (0.02)	−0.14 (0.3)	0.074 (0.6)	−0.044 (0.7)	−0.11 (0.4)	−0.048 (0.7)	−0.11 (0.4)	−0.16 (0.3)	−0.13 (0.3)	−0.29 (0.03)	−0.21 (0.1)	0.047 (0.7)	−0.43 (8e−04)	−0.32 (0.02)	−0.43 (8e−04)	0.014 (0.9)	−0.3 (0.02)	−0.093 (0.5)	0.0012 (1)	−0.15 (0.3)	−0.0084 (1)	−0.095 (0.5)	−0.26 (0.05)	−0.28 (0.04)
−0.15 (0.3)	−0.055 (0.7)	−0.041 (0.8)	−0.11 (0.4)	−0.35 (0.007)	−0.17 (0.2)	−0.46 (4e−04)	−0.3 (0.03)	−0.17 (0.2)	−0.23 (0.08)	−0.23 (0.09)	−0.14 (0.3)	0.094 (0.5)	0.0084 (1)	−0.12 (0.4)	−0.14 (0.3)	0.016 (0.9)	−0.016 (0.9)	−0.18 (0.2)	−0.14 (0.3)	−0.0094 (0.9)	−0.15 (0.3)	0.028 (0.8)	−0.058 (0.7)	−0.12 (0.4)	−0.14 (0.3)	−0.023 (0.9)	−0.18 (0.2)	−0.058 (0.7)
−0.037 (0.8)	0.2 (0.1)	0.24 (0.08)	0.16 (0.3)	−0.15 (0.3)	0.094 (0.5)	−0.4 (0.003)	−0.25 (0.07)	−0.12 (0.4)	−0.33 (0.01)	−0.3 (0.02)	−0.13 (0.3)	0.12 (0.4)	−0.008 (1)	−0.15 (0.3)	−0.076 (0.6)	0.26 (0.06)	−0.26 (0.06)	−0.26 (0.05)	−0.35 (0.009)	−0.11 (0.4)	−0.19 (0.2)	0.064 (0.6)	0.11 (0.4)	−0.019 (0.9)	0.084 (0.5)	0.12 (0.4)	−0.085 (0.5)	−0.33 (0.01)
0.036 (0.8)	−0.018 (0.9)	0.12 (0.4)	0.27 (0.04)	−0.21 (0.1)	0.046 (0.7)	−0.17 (0.2)	−0.19 (0.2)	−0.23 (0.09)	−0.19 (0.2)	−0.25 (0.07)	−0.0022 (1)	0.18 (0.2)	−0.05 (0.7)	0.054 (0.7)	0.15 (0.3)	0.25 (0.06)	0.06 (0.7)	−0.15 (0.3)	−0.15 (0.3)	−0.13 (0.3)	−0.052 (0.7)	0.038 (0.8)	−0.018 (0.9)	0.1 (0.5)	−0.019 (0.9)	0.17 (0.2)	0.067 (0.6)	−0.23 (0.1)
0.052 (0.7)	−0.15 (0.3)	−0.23 (0.09)	−0.087 (0.5)	0.21 (0.1)	0.067 (0.6)	0.26 (0.06)	0.087 (0.5)	0.18 (0.2)	0.24 (0.08)	0.59 (2e−06)	0.019 (0.9)	−0.16 (0.2)	0.057 (0.7)	−0.044 (0.7)	0.13 (0.3)	−0.11 (0.4)	0.063 (0.6)	0.38 (0.004)	0.33 (0.01)	0.092 (0.5)	0.083 (0.5)	−0.069 (0.6)	0.059 (0.7)	0.013 (0.9)	0.12 (0.4)	−0.1 (0.4)	0.17 (0.2)	0.18 (0.2)

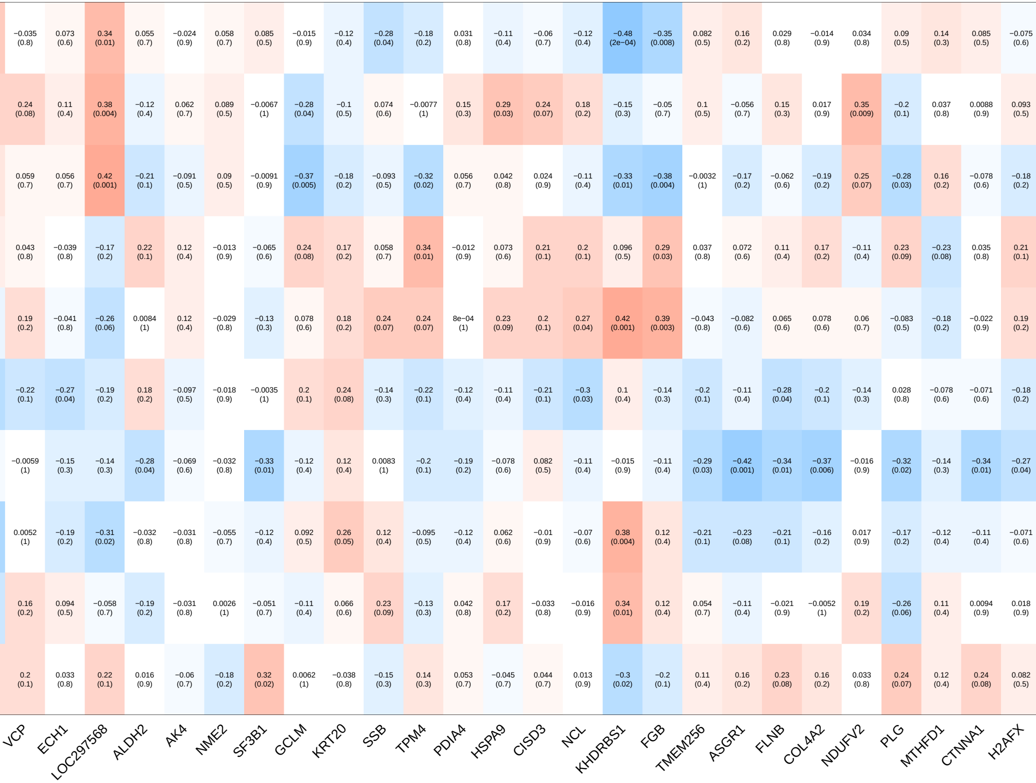

−0.035 (0.8) 0.073 (0.6) 0.34 (0.01) 0.055 (0.7) −0.024 (0.9) 0.058 (0.7) 0.085 (0.5) −0.015 (0.9) −0.12 (0.4) −0.28 (0.04) −0.18 (0.2) 0.031 (0.8) −0.11 (0.4) −0.06 (0.7) −0.12 (0.4) −0.48 (2e−04) −0.35 (0.008) 0.082 (0.5) 0.16 (0.2) 0.029 (0.8) −0.014 (0.9) 0.034 (0.8) 0.09 (0.5) 0.14 (0.3) 0.085 (0.5) −0.075 (0.6)
0.24 (0.08) 0.11 (0.4) 0.38 (0.004) −0.12 (0.4) 0.062 (0.7) 0.089 (0.5) −0.0067 (1) −0.28 (0.04) −0.1 (0.5) 0.074 (0.6) −0.0077 (1) 0.15 (0.3) 0.29 (0.03) 0.24 (0.07) 0.18 (0.2) −0.15 (0.3) −0.05 (0.7) 0.1 (0.5) −0.056 (0.7) 0.15 (0.3) 0.017 (0.9) 0.35 (0.009) −0.2 (0.1) 0.037 (0.8) 0.0088 (0.9) 0.093 (0.5)
0.059 (0.7) 0.056 (0.7) 0.42 (0.001) −0.21 (0.1) −0.091 (0.5) 0.09 (0.5) −0.0091 (0.9) −0.37 (0.005) −0.18 (0.2) −0.093 (0.5) −0.32 (0.02) 0.056 (0.7) 0.042 (0.8) 0.024 (0.9) −0.11 (0.4) −0.33 (0.01) −0.38 (0.004) −0.0032 (1) −0.17 (0.2) −0.062 (0.6) −0.19 (0.2) 0.25 (0.07) −0.28 (0.03) 0.16 (0.2) −0.078 (0.6) −0.18 (0.2)
0.043 (0.8) −0.039 (0.8) −0.17 (0.2) 0.22 (0.1) 0.12 (0.4) −0.013 (0.9) −0.065 (0.6) 0.24 (0.08) 0.17 (0.2) 0.058 (0.7) 0.34 (0.01) −0.012 (0.9) 0.073 (0.6) 0.21 (0.1) 0.2 (0.1) 0.096 (0.5) 0.29 (0.03) 0.037 (0.8) 0.072 (0.6) 0.11 (0.4) 0.17 (0.2) −0.11 (0.4) 0.23 (0.09) −0.23 (0.08) 0.035 (0.8) 0.21 (0.1)
0.19 (0.2) −0.041 (0.8) −0.26 (0.06) 0.0084 (1) 0.12 (0.4) −0.029 (0.8) −0.13 (0.3) 0.078 (0.6) 0.18 (0.2) 0.24 (0.07) 0.24 (0.07) 8e−04 (1) 0.23 (0.09) 0.2 (0.1) 0.27 (0.04) 0.42 (0.001) 0.39 (0.003) −0.043 (0.8) −0.082 (0.6) 0.065 (0.6) 0.078 (0.6) 0.06 (0.7) −0.083 (0.5) −0.18 (0.2) −0.022 (0.9) 0.19 (0.2)
−0.22 (0.1) −0.27 (0.04) −0.19 (0.2) 0.18 (0.2) −0.097 (0.5) −0.018 (0.9) −0.0035 (1) 0.2 (0.1) 0.24 (0.08) −0.14 (0.3) −0.22 (0.1) −0.12 (0.4) −0.11 (0.4) −0.21 (0.1) −0.3 (0.03) 0.1 (0.4) −0.14 (0.3) −0.2 (0.1) −0.11 (0.4) −0.28 (0.04) −0.2 (0.1) −0.14 (0.3) 0.028 (0.8) −0.078 (0.6) −0.071 (0.6) −0.18 (0.2)
−0.0059 (1) −0.15 (0.3) −0.14 (0.3) −0.28 (0.04) −0.069 (0.6) −0.032 (0.8) −0.33 (0.01) −0.12 (0.4) 0.12 (0.4) 0.0083 (1) −0.2 (0.1) −0.19 (0.2) −0.078 (0.6) 0.082 (0.5) −0.11 (0.4) −0.015 (0.9) −0.11 (0.4) −0.29 (0.03) −0.42 (0.001) −0.34 (0.01) −0.37 (0.006) −0.016 (0.9) −0.32 (0.02) −0.14 (0.3) −0.34 (0.01) −0.27 (0.04)
0.0052 (1) −0.19 (0.2) −0.31 (0.02) −0.032 (0.8) −0.031 (0.8) −0.055 (0.7) −0.12 (0.4) 0.092 (0.5) 0.26 (0.05) 0.12 (0.4) −0.095 (0.5) −0.12 (0.4) 0.062 (0.6) −0.01 (0.9) −0.07 (0.6) 0.38 (0.004) 0.12 (0.4) −0.21 (0.1) −0.23 (0.08) −0.21 (0.1) −0.16 (0.2) 0.017 (0.9) −0.17 (0.2) −0.12 (0.4) −0.11 (0.4) −0.071 (0.6)
0.16 (0.2) 0.094 (0.5) −0.058 (0.7) −0.19 (0.2) −0.031 (0.8) 0.0026 (1) −0.051 (0.7) −0.11 (0.4) 0.066 (0.6) 0.23 (0.09) −0.13 (0.3) 0.042 (0.8) 0.17 (0.2) −0.033 (0.8) −0.016 (0.9) 0.34 (0.01) 0.12 (0.4) 0.054 (0.7) −0.11 (0.4) −0.021 (0.9) −0.0052 (1) 0.19 (0.2) −0.26 (0.06) 0.11 (0.4) 0.0094 (0.9) 0.018 (0.9)
0.2 (0.1) 0.033 (0.8) 0.22 (0.1) 0.016 (0.9) −0.06 (0.7) −0.18 (0.2) 0.32 (0.02) 0.0062 (1) −0.038 (0.8) −0.15 (0.3) 0.14 (0.3) 0.053 (0.7) −0.045 (0.7) 0.044 (0.7) 0.013 (0.9) −0.3 (0.02) −0.2 (0.1) 0.11 (0.4) 0.16 (0.2) 0.23 (0.08) 0.16 (0.2) 0.033 (0.8) 0.24 (0.07) 0.12 (0.4) 0.24 (0.08) 0.082 (0.5)
VCP
ECH1
LOC297568
ALDH2
AK4
NME2
SF3B1
GCLM
KRT20
SSB
TPM4
PDIA4
HSPA9
CISD3
NCL
KHDRBS1
FGB
TMEM256
ASGR1
FLNB
COL4A2
NDUFV2
PLG
MTHFD1
CTNNA1
H2AFX

HBB	HIST1H1C	TPM2	F1LNG6	PRPH	FGA	ITGB1	HNRNPU	COL4A5	CDH2	SCP2	RPS5	VAT1	CALR4	HNRNPK	HBA−A2	CHCHD3	LOC100912599	RT1−M6−1	NDUFA6	HIST1H1D	HNRNPC	HNRNPAB	TPM1	RPS19L2	NDUFA7	A0A0G2JSW2	GLUL	NPM1	SRSF7	LOC100360647
0.00026 (1)	−0.01 (0.9)	−0.29 (0.03)	0.002 (1)	−0.44 (8e−04)	−0.43 (0.001)	−0.13 (0.4)	−0.15 (0.3)	0.019 (0.9)	0.048 (0.7)	0.15 (0.3)	0.056 (0.7)	−0.1 (0.5)	0.062 (0.6)	−0.33 (0.01)	−0.015 (0.9)	−0.033 (0.8)	−0.09 (0.5)	0.043 (0.8)	−0.038 (0.8)	−0.2 (0.1)	−0.16 (0.2)	−0.21 (0.1)	−0.033 (0.8)	−0.04 (0.8)	0.017 (0.9)	0.035 (0.8)	0.073 (0.6)	−0.067 (0.6)	−0.051 (0.7)	−0.0053 (1)
0.24 (0.07)	0.18 (0.2)	0.38 (0.004)	0.19 (0.2)	0.12 (0.4)	0.029 (0.8)	0.29 (0.03)	0.27 (0.05)	−0.31 (0.02)	−0.098 (0.5)	0.15 (0.3)	0.22 (0.1)	0.23 (0.09)	0.074 (0.6)	0.069 (0.6)	0.23 (0.08)	0.11 (0.4)	0.15 (0.3)	−0.023 (0.9)	0.14 (0.3)	0.13 (0.4)	0.12 (0.4)	0.17 (0.2)	0.13 (0.3)	0.23 (0.08)	0.18 (0.2)	0.15 (0.3)	0.26 (0.05)	0.17 (0.2)	0.099 (0.5)	0.051 (0.7)
−0.015 (0.9)	−0.099 (0.5)	−0.12 (0.4)	−0.14 (0.3)	−0.099 (0.5)	−0.37 (0.005)	−0.01 (0.9)	0.03 (0.8)	−0.22 (0.1)	−0.16 (0.2)	0.25 (0.06)	0.12 (0.4)	0.1 (0.5)	−0.044 (0.7)	−0.21 (0.1)	0.041 (0.8)	−0.039 (0.8)	−0.033 (0.8)	−0.12 (0.4)	0.009 (0.9)	−0.15 (0.3)	−0.14 (0.3)	−0.039 (0.8)	−0.22 (0.1)	0.039 (0.8)	0.097 (0.5)	0.039 (0.8)	0.044 (0.7)	−0.094 (0.5)	0.005 (1)	−0.085 (0.5)
0.14 (0.3)	0.2 (0.1)	0.32 (0.02)	0.27 (0.04)	0.023 (0.9)	0.31 (0.02)	0.18 (0.2)	0.034 (0.8)	−0.044 (0.7)	0.17 (0.2)	−0.24 (0.07)	0.0034 (1)	−0.094 (0.5)	0.11 (0.4)	0.18 (0.2)	0.013 (0.9)	0.036 (0.8)	0.05 (0.7)	0.04 (0.8)	−0.054 (0.7)	0.15 (0.3)	0.11 (0.4)	−0.022 (0.9)	0.3 (0.02)	0.039 (0.8)	−0.11 (0.4)	−0.019 (0.9)	0.058 (0.7)	0.15 (0.3)	−0.033 (0.8)	0.084 (0.5)
0.13 (0.3)	0.14 (0.3)	0.53 (2e−05)	0.19 (0.2)	0.43 (8e−04)	0.51 (7e−05)	0.28 (0.04)	0.26 (0.06)	−0.16 (0.3)	−0.057 (0.7)	−0.15 (0.3)	−0.017 (0.9)	0.14 (0.3)	−0.00054 (1)	0.36 (0.007)	0.1 (0.5)	0.1 (0.4)	0.15 (0.3)	−0.0083 (1)	0.084 (0.5)	0.26 (0.05)	0.25 (0.06)	0.28 (0.04)	0.24 (0.07)	0.13 (0.4)	0.039 (0.8)	0.018 (0.9)	0.093 (0.5)	0.2 (0.1)	0.031 (0.8)	0.038 (0.8)
−0.26 (0.05)	−0.27 (0.05)	−0.38 (0.004)	−0.31 (0.02)	−0.019 (0.9)	−0.1 (0.5)	−0.21 (0.1)	−0.34 (0.01)	0.073 (0.6)	0.008 (1)	−0.2 (0.1)	−0.033 (0.8)	−0.22 (0.1)	−0.14 (0.3)	−0.041 (0.8)	−0.15 (0.3)	−0.088 (0.5)	−0.2 (0.1)	−0.056 (0.7)	−0.25 (0.06)	−0.17 (0.2)	−0.2 (0.1)	−0.15 (0.3)	−0.058 (0.7)	−0.22 (0.1)	−0.13 (0.3)	−0.14 (0.3)	−0.19 (0.2)	−0.22 (0.1)	−0.081 (0.6)	−0.068 (0.6)
−0.23 (0.08)	−0.32 (0.02)	−0.16 (0.2)	−0.35 (0.008)	0.1 (0.4)	−0.11 (0.4)	−0.087 (0.5)	−0.071 (0.6)	−0.26 (0.05)	−0.051 (0.7)	0.058 (0.7)	−0.18 (0.2)	−0.055 (0.7)	−0.2 (0.1)	0.013 (0.9)	−0.2 (0.1)	−0.19 (0.2)	−0.16 (0.3)	−0.32 (0.02)	−0.19 (0.2)	−0.18 (0.2)	−0.14 (0.3)	−0.058 (0.7)	−0.4 (0.002)	−0.18 (0.2)	−0.15 (0.3)	−0.24 (0.08)	−0.33 (0.01)	−0.31 (0.02)	−0.12 (0.4)	−0.23 (0.09)
−0.14 (0.3)	−0.16 (0.2)	0.045 (0.7)	−0.19 (0.2)	0.38 (0.004)	0.22 (0.1)	0.024 (0.9)	−0.0085 (1)	−0.056 (0.7)	−0.088 (0.5)	−0.13 (0.3)	−0.079 (0.6)	−0.0072 (1)	−0.16 (0.2)	0.23 (0.09)	−0.05 (0.7)	−0.0083 (1)	−0.03 (0.8)	−0.1 (0.4)	−0.088 (0.5)	0.031 (0.8)	−0.0013 (1)	0.14 (0.3)	−0.068 (0.6)	−0.08 (0.6)	−0.044 (0.7)	−0.12 (0.4)	−0.16 (0.2)	−0.069 (0.6)	−0.03 (0.8)	−0.079 (0.6)
0.041 (0.8)	−0.051 (0.7)	0.15 (0.3)	−0.056 (0.7)	0.45 (5e−04)	0.18 (0.2)	0.093 (0.5)	0.27 (0.04)	−0.023 (0.9)	−0.17 (0.2)	0.099 (0.5)	0.027 (0.8)	0.28 (0.04)	−0.046 (0.7)	0.26 (0.06)	0.091 (0.5)	0.096 (0.5)	0.14 (0.3)	0.018 (0.9)	0.2 (0.1)	0.17 (0.2)	0.17 (0.2)	0.29 (0.03)	−0.085 (0.5)	0.14 (0.3)	0.14 (0.3)	0.021 (0.9)	−0.02 (0.9)	0.086 (0.5)	0.11 (0.4)	0.018 (0.9)
0.13 (0.3)	0.23 (0.08)	−0.077 (0.6)	0.16 (0.2)	−0.31 (0.02)	−0.26 (0.05)	0.0063 (1)	−0.089 (0.5)	0.12 (0.4)	0.16 (0.3)	0.22 (0.1)	0.11 (0.4)	−0.002 (1)	−0.0098 (0.9)	−0.19 (0.2)	0.091 (0.5)	0.041 (0.8)	−0.097 (0.5)	0.083 (0.5)	−0.026 (0.8)	−0.04 (0.8)	−0.11 (0.4)	−0.089 (0.5)	0.075 (0.6)	0.0016 (1)	0.052 (0.7)	0.089 (0.5)	0.18 (0.2)	0.02 (0.9)	−0.00066 (1)	−0.066 (0.6)

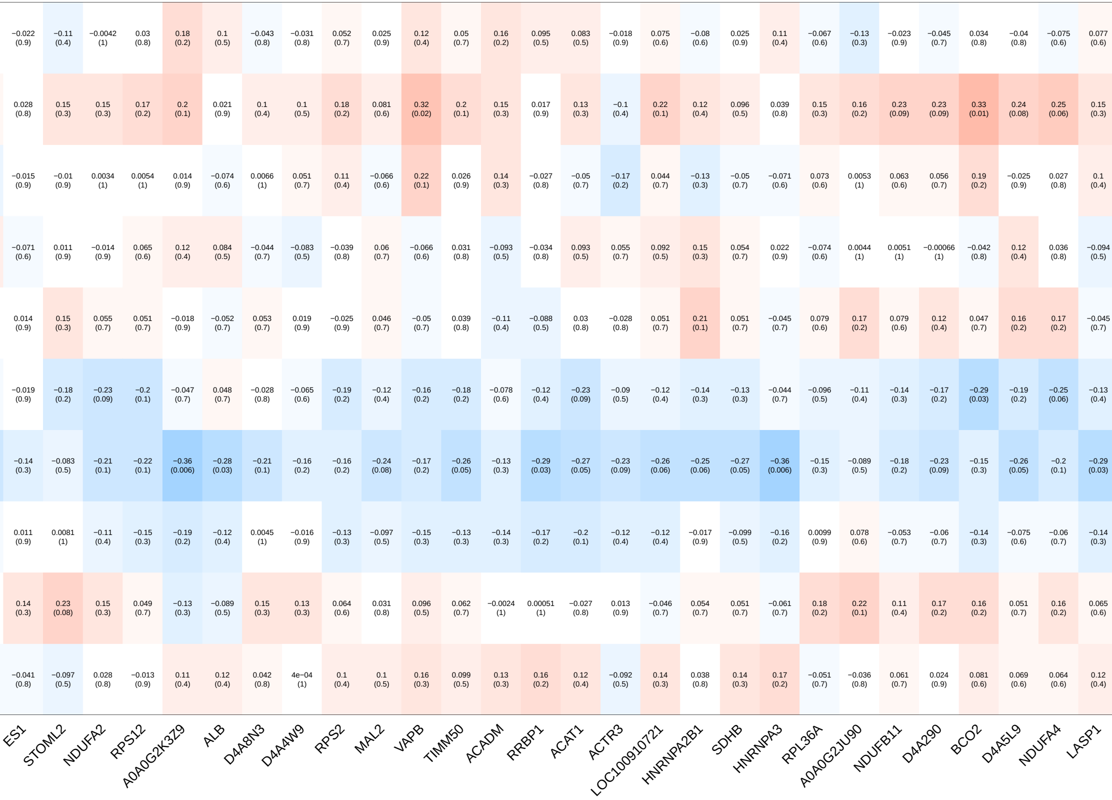

−0.022 (0.9) | −0.11 (0.4) | −0.0042 (1) | 0.03 (0.8) | 0.18 (0.2) | 0.1 (0.5) | −0.043 (0.8) | −0.031 (0.8) | 0.052 (0.7) | 0.025 (0.9) | 0.12 (0.4) | 0.05 (0.7) | 0.16 (0.2) | 0.095 (0.5) | 0.083 (0.5) | −0.018 (0.9) | 0.075 (0.6) | −0.08 (0.6) | 0.025 (0.9) | 0.11 (0.4) | −0.067 (0.6) | −0.13 (0.3) | −0.023 (0.9) | −0.045 (0.7) | 0.034 (0.8) | −0.04 (0.8) | −0.075 (0.6) | 0.077 (0.6)
0.028 (0.8) | 0.15 (0.3) | 0.15 (0.3) | 0.17 (0.2) | 0.2 (0.1) | 0.021 (0.9) | 0.1 (0.4) | 0.1 (0.5) | 0.18 (0.2) | 0.081 (0.6) | 0.32 (0.02) | 0.2 (0.1) | 0.15 (0.3) | 0.017 (0.9) | 0.13 (0.3) | −0.1 (0.4) | 0.22 (0.1) | 0.12 (0.4) | 0.096 (0.5) | 0.039 (0.8) | 0.15 (0.3) | 0.16 (0.2) | 0.23 (0.09) | 0.23 (0.09) | 0.33 (0.01) | 0.24 (0.08) | 0.25 (0.06) | 0.15 (0.3)
−0.015 (0.9) | −0.01 (0.9) | 0.0034 (1) | 0.0054 (1) | 0.014 (0.9) | −0.074 (0.6) | 0.0066 (1) | 0.051 (0.7) | 0.11 (0.4) | −0.066 (0.6) | 0.22 (0.1) | 0.026 (0.9) | 0.14 (0.3) | −0.027 (0.8) | −0.05 (0.7) | −0.17 (0.2) | 0.044 (0.7) | −0.13 (0.3) | −0.05 (0.7) | −0.071 (0.6) | 0.073 (0.6) | 0.0053 (1) | 0.063 (0.6) | 0.056 (0.7) | 0.19 (0.2) | −0.025 (0.9) | 0.027 (0.8) | 0.1 (0.4)
−0.071 (0.6) | 0.011 (0.9) | −0.014 (0.9) | 0.065 (0.6) | 0.12 (0.4) | 0.084 (0.5) | −0.044 (0.7) | −0.083 (0.5) | −0.039 (0.8) | 0.06 (0.7) | −0.066 (0.6) | 0.031 (0.8) | −0.093 (0.5) | −0.034 (0.8) | 0.093 (0.5) | 0.055 (0.7) | 0.092 (0.5) | 0.15 (0.3) | 0.054 (0.7) | 0.022 (0.9) | −0.074 (0.6) | 0.0044 (1) | 0.0051 (1) | −0.00066 (1) | −0.042 (0.8) | 0.12 (0.4) | 0.036 (0.8) | −0.094 (0.5)
0.014 (0.9) | 0.15 (0.3) | 0.055 (0.7) | 0.051 (0.7) | −0.018 (0.9) | −0.052 (0.7) | 0.053 (0.7) | 0.019 (0.9) | −0.025 (0.9) | 0.046 (0.7) | −0.05 (0.7) | 0.039 (0.8) | −0.11 (0.4) | −0.088 (0.5) | 0.03 (0.8) | −0.028 (0.8) | 0.051 (0.7) | 0.21 (0.1) | 0.051 (0.7) | −0.045 (0.7) | 0.079 (0.6) | 0.17 (0.2) | 0.079 (0.6) | 0.12 (0.4) | 0.047 (0.7) | 0.16 (0.2) | 0.17 (0.2) | −0.045 (0.7)
−0.019 (0.9) | −0.18 (0.2) | −0.23 (0.09) | −0.2 (0.1) | −0.047 (0.7) | 0.048 (0.7) | −0.028 (0.8) | −0.065 (0.6) | −0.19 (0.2) | −0.12 (0.4) | −0.16 (0.2) | −0.18 (0.2) | −0.078 (0.6) | −0.12 (0.4) | −0.23 (0.09) | −0.09 (0.5) | −0.12 (0.4) | −0.14 (0.3) | −0.13 (0.3) | −0.044 (0.7) | −0.096 (0.5) | −0.11 (0.4) | −0.14 (0.3) | −0.17 (0.2) | −0.29 (0.03) | −0.19 (0.2) | −0.25 (0.06) | −0.13 (0.4)
−0.14 (0.3) | −0.083 (0.5) | −0.21 (0.1) | −0.22 (0.1) | −0.36 (0.006) | −0.28 (0.03) | −0.21 (0.1) | −0.16 (0.2) | −0.16 (0.2) | −0.24 (0.08) | −0.17 (0.2) | −0.26 (0.05) | −0.13 (0.3) | −0.29 (0.03) | −0.27 (0.05) | −0.23 (0.09) | −0.26 (0.06) | −0.25 (0.06) | −0.27 (0.05) | −0.36 (0.006) | −0.15 (0.3) | −0.089 (0.5) | −0.18 (0.2) | −0.23 (0.09) | −0.15 (0.3) | −0.26 (0.05) | −0.2 (0.1) | −0.29 (0.03)
0.011 (0.9) | 0.0081 (1) | −0.11 (0.4) | −0.15 (0.3) | −0.19 (0.2) | −0.12 (0.4) | 0.0045 (1) | −0.016 (0.9) | −0.13 (0.3) | −0.097 (0.5) | −0.15 (0.3) | −0.13 (0.3) | −0.14 (0.3) | −0.17 (0.2) | −0.2 (0.1) | −0.12 (0.4) | −0.12 (0.4) | −0.017 (0.9) | −0.099 (0.5) | −0.16 (0.2) | 0.0099 (0.9) | 0.078 (0.6) | −0.053 (0.7) | −0.06 (0.7) | −0.14 (0.3) | −0.075 (0.6) | −0.06 (0.7) | −0.14 (0.3)
0.14 (0.3) | 0.23 (0.08) | 0.15 (0.3) | 0.049 (0.7) | −0.13 (0.3) | −0.089 (0.5) | 0.15 (0.3) | 0.13 (0.3) | 0.064 (0.6) | 0.031 (0.8) | 0.096 (0.5) | 0.062 (0.7) | −0.0024 (1) | 0.00051 (1) | −0.027 (0.8) | 0.013 (0.9) | −0.046 (0.7) | 0.054 (0.7) | 0.051 (0.7) | −0.061 (0.7) | 0.18 (0.2) | 0.22 (0.1) | 0.11 (0.4) | 0.17 (0.2) | 0.16 (0.2) | 0.051 (0.7) | 0.16 (0.2) | 0.065 (0.6)
−0.041 (0.8) | −0.097 (0.5) | 0.028 (0.8) | −0.013 (0.9) | 0.11 (0.4) | 0.12 (0.4) | 0.042 (0.8) | 4e−04 (1) | 0.1 (0.4) | 0.1 (0.5) | 0.16 (0.3) | 0.099 (0.5) | 0.13 (0.3) | 0.16 (0.2) | 0.12 (0.4) | −0.092 (0.5) | 0.14 (0.3) | 0.038 (0.8) | 0.14 (0.3) | 0.17 (0.2) | −0.051 (0.7) | −0.036 (0.8) | 0.061 (0.7) | 0.024 (0.9) | 0.081 (0.6) | 0.069 (0.6) | 0.064 (0.6) | 0.12 (0.4)
ES1 | STOML2 | NDUFA2 | RPS12 | A0A0G2K3Z9 | ALB | D4A8N3 | D4A4W9 | RPS2 | MAL2 | VAPB | TIMM50 | ACADM | RRBP1 | ACAT1 | ACTR3 | LOC100910721 | HNRNPA2B1 | SDHB | HNRNPA3 | RPL36A | A0A0G2JU90 | NDUFB11 | D4A290 | BCO2 | D4A5L9 | NDUFA4 | LASP1

Transcriptomic Modules − Molecular features relationships

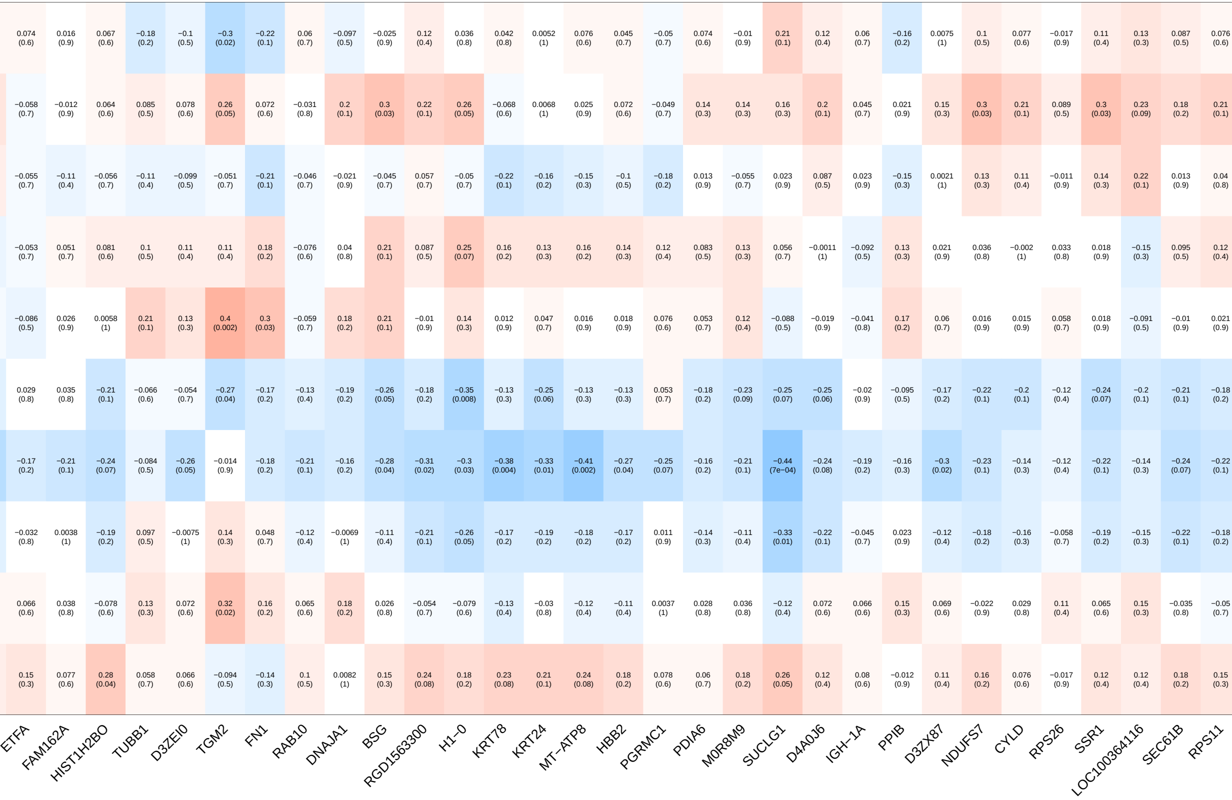

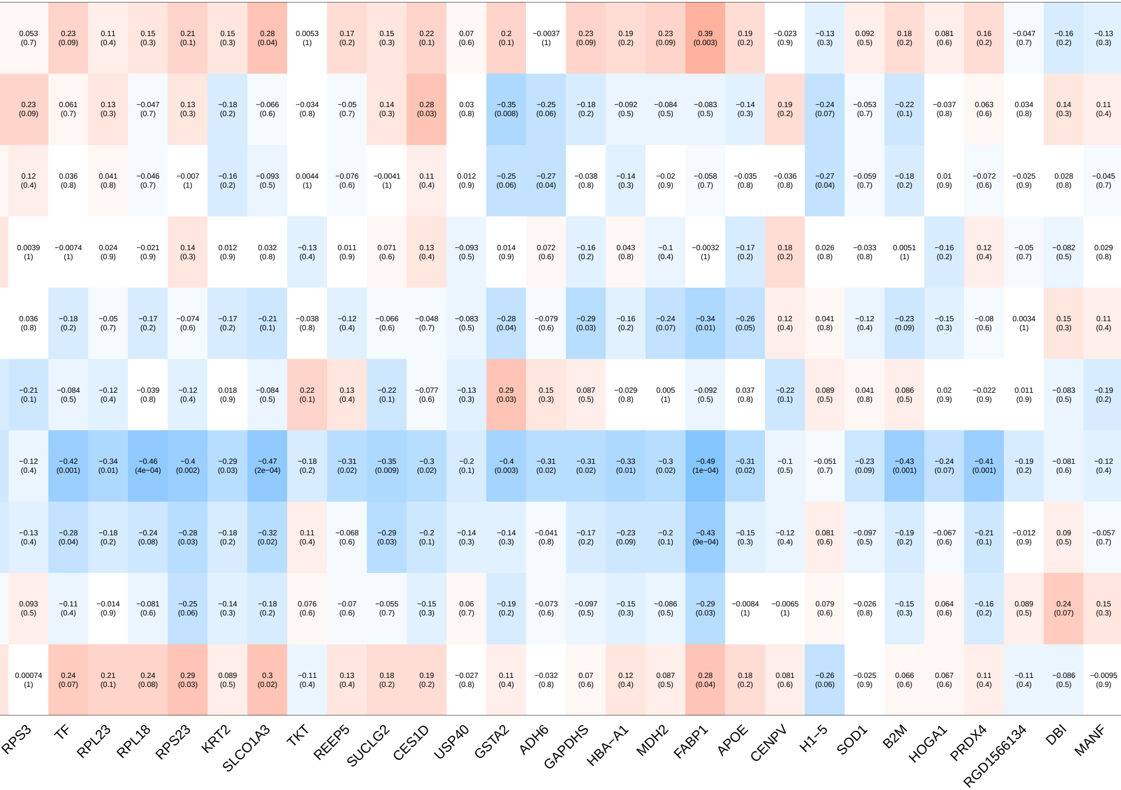
0.053 (0.7) 0.23 (0.09) 0.11 (0.4) 0.15 (0.3) 0.21 (0.1) 0.15 (0.3) 0.28 (0.04) 0.0053 (1) 0.17 (0.2) 0.15 (0.3) 0.22 (0.1) 0.07 (0.6) 0.2 (0.1) −0.0037 (1) 0.23 (0.09) 0.19 (0.2) 0.23 (0.09) 0.39 (0.003) 0.19 (0.2) −0.023 (0.9) −0.13 (0.3) 0.092 (0.5) 0.18 (0.2) 0.081 (0.6) 0.16 (0.2) −0.047 (0.7) −0.16 (0.2) −0.13 (0.3)
0.23 (0.09) 0.061 (0.7) 0.13 (0.3) −0.047 (0.7) 0.13 (0.3) −0.18 (0.2) −0.066 (0.6) −0.034 (0.8) −0.05 (0.7) 0.14 (0.3) 0.28 (0.03) 0.03 (0.8) −0.35 (0.008) −0.25 (0.06) −0.18 (0.2) −0.092 (0.5) −0.084 (0.5) −0.083 (0.5) −0.14 (0.3) 0.19 (0.2) −0.24 (0.07) −0.053 (0.7) −0.22 (0.1) −0.037 (0.8) 0.063 (0.6) 0.034 (0.8) 0.14 (0.3) 0.11 (0.4)
0.12 (0.4) 0.036 (0.8) 0.041 (0.8) −0.046 (0.7) −0.007 (1) −0.16 (0.2) −0.093 (0.5) 0.0044 (1) −0.076 (0.6) −0.0041 (1) 0.11 (0.4) 0.012 (0.9) −0.25 (0.06) −0.27 (0.04) −0.038 (0.8) −0.14 (0.3) −0.02 (0.9) −0.058 (0.7) −0.035 (0.8) −0.036 (0.8) −0.27 (0.04) −0.059 (0.7) −0.18 (0.2) 0.01 (0.9) −0.072 (0.6) −0.025 (0.9) 0.028 (0.8) −0.045 (0.7)
0.0039 (1) −0.0074 (1) 0.024 (0.9) −0.021 (0.9) 0.14 (0.3) 0.012 (0.9) 0.032 (0.8) −0.13 (0.4) 0.011 (0.9) 0.071 (0.6) 0.13 (0.4) −0.093 (0.5) 0.014 (0.9) 0.072 (0.6) −0.16 (0.2) 0.043 (0.8) −0.1 (0.4) −0.0032 (1) −0.17 (0.2) 0.18 (0.2) 0.026 (0.8) −0.033 (0.8) 0.0051 (1) −0.16 (0.2) 0.12 (0.4) −0.05 (0.7) −0.082 (0.5) 0.029 (0.8)
0.036 (0.8) −0.18 (0.2) −0.05 (0.7) −0.17 (0.2) −0.074 (0.6) −0.17 (0.2) −0.21 (0.1) −0.038 (0.8) −0.12 (0.4) −0.066 (0.6) −0.048 (0.7) −0.083 (0.5) −0.28 (0.04) −0.079 (0.6) −0.29 (0.03) −0.16 (0.2) −0.24 (0.07) −0.34 (0.01) −0.26 (0.05) 0.12 (0.4) 0.041 (0.8) −0.12 (0.4) −0.23 (0.09) −0.15 (0.3) −0.08 (0.6) 0.0034 (1) 0.15 (0.3) 0.11 (0.4)
−0.21 (0.1) −0.084 (0.5) −0.12 (0.4) −0.039 (0.8) −0.12 (0.4) 0.018 (0.9) −0.084 (0.5) 0.22 (0.1) 0.13 (0.4) −0.22 (0.1) −0.077 (0.6) −0.13 (0.3) 0.29 (0.03) 0.15 (0.3) 0.087 (0.5) −0.029 (0.8) 0.005 (1) −0.092 (0.5) 0.037 (0.8) −0.22 (0.1) 0.089 (0.5) 0.041 (0.8) 0.086 (0.5) 0.02 (0.9) −0.022 (0.9) 0.011 (0.9) −0.083 (0.5) −0.19 (0.2)
−0.12 (0.4) −0.42 (0.001) −0.34 (0.01) −0.46 (4e−04) −0.4 (0.002) −0.29 (0.03) −0.47 (2e−04) −0.18 (0.2) −0.31 (0.02) −0.35 (0.009) −0.3 (0.02) −0.2 (0.1) −0.4 (0.003) −0.31 (0.02) −0.31 (0.02) −0.33 (0.01) −0.3 (0.02) −0.49 (1e−04) −0.31 (0.02) −0.1 (0.5) −0.051 (0.7) −0.23 (0.09) −0.43 (0.001) −0.24 (0.07) −0.41 (0.001) −0.19 (0.2) −0.081 (0.6) −0.12 (0.4)
−0.13 (0.4) −0.28 (0.04) −0.18 (0.2) −0.24 (0.08) −0.28 (0.03) −0.18 (0.2) −0.32 (0.02) 0.11 (0.4) −0.068 (0.6) −0.29 (0.03) −0.2 (0.1) −0.14 (0.3) −0.14 (0.3) −0.041 (0.8) −0.17 (0.2) −0.23 (0.09) −0.2 (0.1) −0.43 (9e−04) −0.15 (0.3) −0.12 (0.4) 0.081 (0.6) −0.097 (0.5) −0.19 (0.2) −0.067 (0.6) −0.21 (0.1) −0.012 (0.9) 0.09 (0.5) −0.057 (0.7)
0.093 (0.5) −0.11 (0.4) −0.014 (0.9) −0.081 (0.6) −0.25 (0.06) −0.14 (0.3) −0.18 (0.2) 0.076 (0.6) −0.07 (0.6) −0.055 (0.7) −0.15 (0.3) 0.06 (0.7) −0.19 (0.2) −0.073 (0.6) −0.097 (0.5) −0.15 (0.3) −0.086 (0.5) −0.29 (0.03) −0.0084 (1) −0.0065 (1) 0.079 (0.6) −0.026 (0.8) −0.15 (0.3) 0.064 (0.6) −0.16 (0.2) 0.089 (0.5) 0.24 (0.07) 0.15 (0.3)
0.00074 (1) 0.24 (0.07) 0.21 (0.1) 0.24 (0.08) 0.29 (0.03) 0.089 (0.5) 0.3 (0.02) −0.11 (0.4) 0.13 (0.4) 0.18 (0.2) 0.19 (0.2) −0.027 (0.8) 0.11 (0.4) −0.032 (0.8) 0.07 (0.6) 0.12 (0.4) 0.087 (0.5) 0.28 (0.04) 0.18 (0.2) 0.081 (0.6) −0.26 (0.06) −0.025 (0.9) 0.066 (0.6) 0.067 (0.6) 0.11 (0.4) −0.11 (0.4) −0.086 (0.5) −0.0095 (0.9)
RPS3 TF RPL23 RPL18 RPS23 KRT2 SLCO1A3 TKT REEP5 SUCLG2 CES1D USP40 GSTA2 ADH6 GAPDHS HBA−A1 MDH2 FABP1 APOE CENPV H1−5 SOD1 B2M HOGA1 PRDX4 RGD1566134 DBI MANF

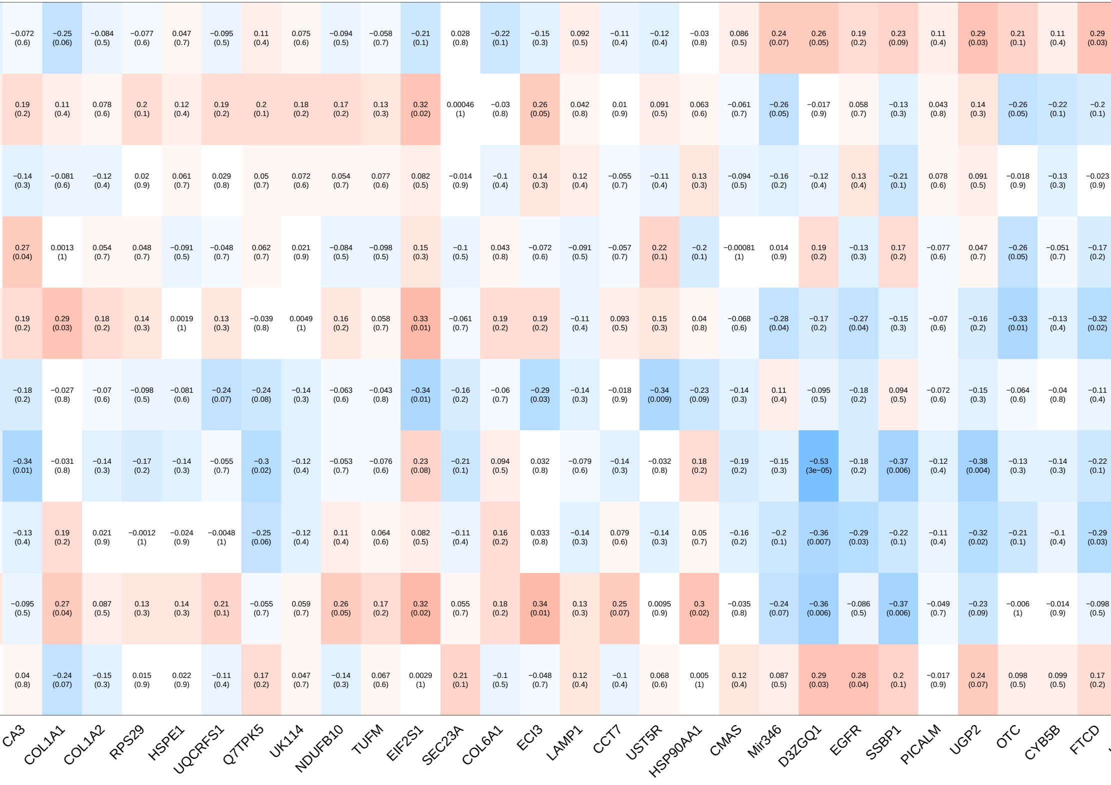

−0.072 (0.6) −0.25 (0.06) −0.084 (0.5) −0.077 (0.6) 0.047 (0.7) −0.095 (0.5) 0.11 (0.4) 0.075 (0.6) −0.094 (0.5) −0.058 (0.7) −0.21 (0.1) 0.028 (0.8) −0.22 (0.1) −0.15 (0.3) 0.092 (0.5) −0.11 (0.4) −0.12 (0.4) −0.03 (0.8) 0.086 (0.5) 0.24 (0.07) 0.26 (0.05) 0.19 (0.2) 0.23 (0.09) 0.11 (0.4) 0.29 (0.03) 0.21 (0.1) 0.11 (0.4) 0.29 (0.03)
0.19 (0.2) 0.11 (0.4) 0.078 (0.6) 0.2 (0.1) 0.12 (0.4) 0.19 (0.2) 0.2 (0.1) 0.18 (0.2) 0.17 (0.2) 0.13 (0.3) 0.32 (0.02) 0.00046 (1) −0.03 (0.8) 0.26 (0.05) 0.042 (0.8) 0.01 (0.9) 0.091 (0.5) 0.063 (0.6) −0.061 (0.7) −0.26 (0.05) −0.017 (0.9) 0.058 (0.7) −0.13 (0.3) 0.043 (0.8) 0.14 (0.3) −0.26 (0.05) −0.22 (0.1) −0.2 (0.1)
−0.14 (0.3) −0.081 (0.6) −0.12 (0.4) 0.02 (0.9) 0.061 (0.7) 0.029 (0.8) 0.05 (0.7) 0.072 (0.6) 0.054 (0.7) 0.077 (0.6) 0.082 (0.5) −0.014 (0.9) −0.1 (0.4) 0.14 (0.3) 0.12 (0.4) −0.055 (0.7) −0.11 (0.4) 0.13 (0.3) −0.094 (0.5) −0.16 (0.2) −0.12 (0.4) 0.13 (0.4) −0.21 (0.1) 0.078 (0.6) 0.091 (0.5) −0.018 (0.9) −0.13 (0.3) −0.023 (0.9)
0.27 (0.04) 0.0013 (1) 0.054 (0.7) 0.048 (0.7) −0.091 (0.5) −0.048 (0.7) 0.062 (0.7) 0.021 (0.9) −0.084 (0.5) −0.098 (0.5) 0.15 (0.3) −0.1 (0.5) 0.043 (0.8) −0.072 (0.6) −0.091 (0.5) −0.057 (0.7) 0.22 (0.1) −0.2 (0.1) −0.00081 (1) 0.014 (0.9) 0.19 (0.2) −0.13 (0.3) 0.17 (0.2) −0.077 (0.6) 0.047 (0.7) −0.26 (0.05) −0.051 (0.7) −0.17 (0.2)
0.19 (0.2) 0.29 (0.03) 0.18 (0.2) 0.14 (0.3) 0.0019 (1) 0.13 (0.3) −0.039 (0.8) 0.0049 (1) 0.16 (0.2) 0.058 (0.7) 0.33 (0.01) −0.061 (0.7) 0.19 (0.2) 0.19 (0.2) −0.11 (0.4) 0.093 (0.5) 0.15 (0.3) 0.04 (0.8) −0.068 (0.6) −0.28 (0.04) −0.17 (0.2) −0.27 (0.04) −0.15 (0.3) −0.07 (0.6) −0.16 (0.2) −0.33 (0.01) −0.13 (0.4) −0.32 (0.02)
−0.18 (0.2) −0.027 (0.8) −0.07 (0.6) −0.098 (0.5) −0.081 (0.6) −0.24 (0.07) −0.24 (0.08) −0.14 (0.3) −0.063 (0.6) −0.043 (0.8) −0.34 (0.01) −0.16 (0.2) −0.06 (0.7) −0.29 (0.03) −0.14 (0.3) −0.018 (0.9) −0.34 (0.009) −0.23 (0.09) −0.14 (0.3) 0.11 (0.4) −0.095 (0.5) −0.18 (0.2) 0.094 (0.5) −0.072 (0.6) −0.15 (0.3) −0.064 (0.6) −0.04 (0.8) −0.11 (0.4)
−0.34 (0.01) −0.031 (0.8) −0.14 (0.3) −0.17 (0.2) −0.14 (0.3) −0.055 (0.7) −0.3 (0.02) −0.12 (0.4) −0.053 (0.7) −0.076 (0.6) 0.23 (0.08) −0.21 (0.1) 0.094 (0.5) 0.032 (0.8) −0.079 (0.6) −0.14 (0.3) −0.032 (0.8) 0.18 (0.2) −0.19 (0.2) −0.15 (0.3) −0.53 (3e−05) −0.18 (0.2) −0.37 (0.006) −0.12 (0.4) −0.38 (0.004) −0.13 (0.3) −0.14 (0.3) −0.22 (0.1)
−0.13 (0.4) 0.19 (0.2) 0.021 (0.9) −0.0012 (1) −0.024 (0.9) −0.0048 (1) −0.25 (0.06) −0.12 (0.4) 0.11 (0.4) 0.064 (0.6) 0.082 (0.5) −0.11 (0.4) 0.16 (0.2) 0.033 (0.8) −0.14 (0.3) 0.079 (0.6) −0.14 (0.3) 0.05 (0.7) −0.16 (0.2) −0.2 (0.1) −0.36 (0.007) −0.29 (0.03) −0.22 (0.1) −0.11 (0.4) −0.32 (0.02) −0.21 (0.1) −0.1 (0.4) −0.29 (0.03)
−0.095 (0.5) 0.27 (0.04) 0.087 (0.5) 0.13 (0.3) 0.14 (0.3) 0.21 (0.1) −0.055 (0.7) 0.059 (0.7) 0.26 (0.05) 0.17 (0.2) 0.32 (0.02) 0.055 (0.7) 0.18 (0.2) 0.34 (0.01) 0.13 (0.3) 0.25 (0.07) 0.0095 (0.9) 0.3 (0.02) −0.035 (0.8) −0.24 (0.07) −0.36 (0.006) −0.086 (0.5) −0.37 (0.006) −0.049 (0.7) −0.23 (0.09) −0.006 (1) −0.014 (0.9) −0.098 (0.5)
0.04 (0.8) −0.24 (0.07) −0.15 (0.3) 0.015 (0.9) 0.022 (0.9) −0.11 (0.4) 0.17 (0.2) 0.047 (0.7) −0.14 (0.3) 0.067 (0.6) 0.0029 (1) 0.21 (0.1) −0.1 (0.5) −0.048 (0.7) 0.12 (0.4) −0.1 (0.4) 0.068 (0.6) 0.005 (1) 0.12 (0.4) 0.087 (0.5) 0.29 (0.03) 0.28 (0.04) 0.2 (0.1) −0.017 (0.9) 0.24 (0.07) 0.098 (0.5) 0.099 (0.5) 0.17 (0.2)
CA3
COL1A1
COL1A2
RPS29
HSPE1
UQCRFS1
Q7TPK5
UK114
NDUFB10
TUFM
EIF2S1
SEC23A
COL6A1
ECI3
LAMP1
CCT7
UST5R
HSP90AA1
CMAS
Mir346
D3ZGQ1
EGFR
SSBP1
PICALM
UGP2
OTC
CYB5B
FTCD

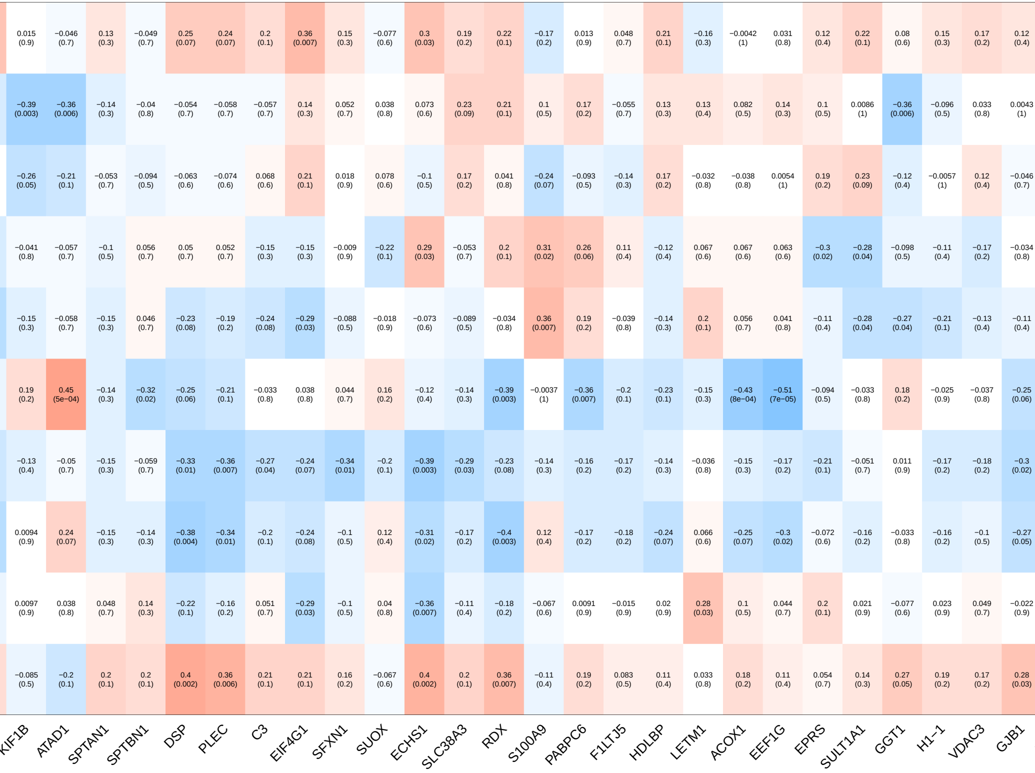

0.015 (0.9) −0.046 (0.7) 0.13 (0.3) −0.049 (0.7) 0.25 (0.07) 0.24 (0.07) 0.2 (0.1) 0.36 (0.007) 0.15 (0.3) −0.077 (0.6) 0.3 (0.03) 0.19 (0.2) 0.22 (0.1) −0.17 (0.2) 0.013 (0.9) 0.048 (0.7) 0.21 (0.1) −0.16 (0.3) −0.0042 (1) 0.031 (0.8) 0.12 (0.4) 0.22 (0.1) 0.08 (0.6) 0.15 (0.3) 0.17 (0.2) 0.12 (0.4)
−0.39 (0.003) −0.36 (0.006) −0.14 (0.3) −0.04 (0.8) −0.054 (0.7) −0.058 (0.7) −0.057 (0.7) 0.14 (0.3) 0.052 (0.7) 0.038 (0.8) 0.073 (0.6) 0.23 (0.09) 0.21 (0.1) 0.1 (0.5) 0.17 (0.2) −0.055 (0.7) 0.13 (0.3) 0.13 (0.4) 0.082 (0.5) 0.14 (0.3) 0.1 (0.5) 0.0086 (1) −0.36 (0.006) −0.096 (0.5) 0.033 (0.8) 0.0043 (1)
−0.26 (0.05) −0.21 (0.1) −0.053 (0.7) −0.094 (0.5) −0.063 (0.6) −0.074 (0.6) 0.068 (0.6) 0.21 (0.1) 0.018 (0.9) 0.078 (0.6) −0.1 (0.5) 0.17 (0.2) 0.041 (0.8) −0.24 (0.07) −0.093 (0.5) −0.14 (0.3) 0.17 (0.2) −0.032 (0.8) −0.038 (0.8) 0.0054 (1) 0.19 (0.2) 0.23 (0.09) −0.12 (0.4) −0.0057 (1) 0.12 (0.4) −0.046 (0.7)
−0.041 (0.8) −0.057 (0.7) −0.1 (0.5) 0.056 (0.7) 0.05 (0.7) 0.052 (0.7) −0.15 (0.3) −0.15 (0.3) −0.009 (0.9) −0.22 (0.1) 0.29 (0.03) −0.053 (0.7) 0.2 (0.1) 0.31 (0.02) 0.26 (0.06) 0.11 (0.4) −0.12 (0.4) 0.067 (0.6) 0.067 (0.6) 0.063 (0.6) −0.3 (0.02) −0.28 (0.04) −0.098 (0.5) −0.11 (0.4) −0.17 (0.2) −0.034 (0.8)
−0.15 (0.3) −0.058 (0.7) −0.15 (0.3) 0.046 (0.7) −0.23 (0.08) −0.19 (0.2) −0.24 (0.08) −0.29 (0.03) −0.088 (0.5) −0.018 (0.9) −0.073 (0.6) −0.089 (0.5) −0.034 (0.8) 0.36 (0.007) 0.19 (0.2) −0.039 (0.8) −0.14 (0.3) 0.2 (0.1) 0.056 (0.7) 0.041 (0.8) −0.11 (0.4) −0.28 (0.04) −0.27 (0.04) −0.21 (0.1) −0.13 (0.4) −0.11 (0.4)
0.19 (0.2) 0.45 (5e−04) −0.14 (0.3) −0.32 (0.02) −0.25 (0.06) −0.21 (0.1) −0.033 (0.8) 0.038 (0.8) 0.044 (0.7) 0.16 (0.2) −0.12 (0.4) −0.14 (0.3) −0.39 (0.003) −0.0037 (1) −0.36 (0.007) −0.2 (0.1) −0.23 (0.1) −0.15 (0.3) −0.43 (8e−04) −0.51 (7e−05) −0.094 (0.5) −0.033 (0.8) 0.18 (0.2) −0.025 (0.9) −0.037 (0.8) −0.25 (0.06)
−0.13 (0.4) −0.05 (0.7) −0.15 (0.3) −0.059 (0.7) −0.33 (0.01) −0.36 (0.007) −0.27 (0.04) −0.24 (0.07) −0.34 (0.01) −0.2 (0.1) −0.39 (0.003) −0.29 (0.03) −0.23 (0.08) −0.14 (0.3) −0.16 (0.2) −0.17 (0.2) −0.14 (0.3) −0.036 (0.8) −0.15 (0.3) −0.17 (0.2) −0.21 (0.1) −0.051 (0.7) 0.011 (0.9) −0.17 (0.2) −0.18 (0.2) −0.3 (0.02)
0.0094 (0.9) 0.24 (0.07) −0.15 (0.3) −0.14 (0.3) −0.38 (0.004) −0.34 (0.01) −0.2 (0.1) −0.24 (0.08) −0.1 (0.5) 0.12 (0.4) −0.31 (0.02) −0.17 (0.2) −0.4 (0.003) 0.12 (0.4) −0.17 (0.2) −0.18 (0.2) −0.24 (0.07) 0.066 (0.6) −0.25 (0.07) −0.3 (0.02) −0.072 (0.6) −0.16 (0.2) −0.033 (0.8) −0.16 (0.2) −0.1 (0.5) −0.27 (0.05)
0.0097 (0.9) 0.038 (0.8) 0.048 (0.7) 0.14 (0.3) −0.22 (0.1) −0.16 (0.2) 0.051 (0.7) −0.29 (0.03) −0.1 (0.5) 0.04 (0.8) −0.36 (0.007) −0.11 (0.4) −0.18 (0.2) −0.067 (0.6) 0.0091 (0.9) −0.015 (0.9) 0.02 (0.9) 0.28 (0.03) 0.1 (0.5) 0.044 (0.7) 0.2 (0.1) 0.021 (0.9) −0.077 (0.6) 0.023 (0.9) 0.049 (0.7) −0.022 (0.9)
−0.085 (0.5) −0.2 (0.1) 0.2 (0.1) 0.2 (0.1) 0.4 (0.002) 0.36 (0.006) 0.21 (0.1) 0.21 (0.1) 0.16 (0.2) −0.067 (0.6) 0.4 (0.002) 0.2 (0.1) 0.36 (0.007) −0.11 (0.4) 0.19 (0.2) 0.083 (0.5) 0.11 (0.4) 0.033 (0.8) 0.18 (0.2) 0.11 (0.4) 0.054 (0.7) 0.14 (0.3) 0.27 (0.05) 0.19 (0.2) 0.17 (0.2) 0.28 (0.03)
KIF1B ATAD1 SPTAN1 SPTBN1 DSP PLEC C3 EIF4G1 SFXN1 SUOX ECHS1 SLC38A3 RDX S100A9 PABPC6 F1LTJ5 HDLBP LETM1 ACOX1 EEF1G EPRS SULT1A1 GGT1 H1−1 VDAC3 GJB1

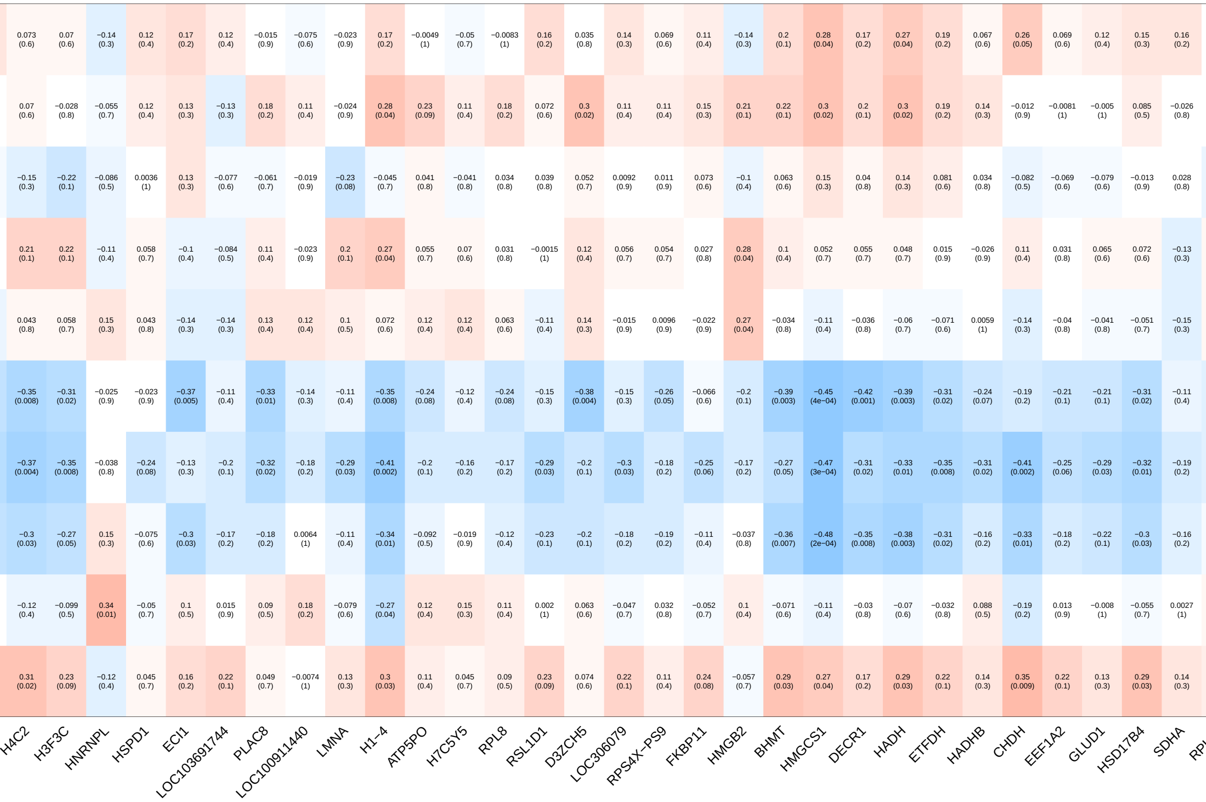

H4C2
H3F3C
HNRNPL
HSPD1
ECI1
LOC103691744
PLAC8
LOC100911440
LMNA
H1-4
ATP5PO
H7C5Y5
RPL8
RSL1D1
D3ZCH5
LOC306079
RPS4X-PS9
FKBP11
HMGB2
BHMT
HMGCS1
DECR1
HADH
ETFDH
HADHB
CHDH
EEF1A2
GLUD1
HSD17B4
SDHA
0.073 (0.6) 0.07 (0.6) −0.14 (0.3) 0.12 (0.4) 0.17 (0.2) 0.12 (0.4) −0.015 (0.9) −0.075 (0.6) −0.023 (0.9) 0.17 (0.2) −0.0049 (1) −0.05 (0.7) −0.0083 (1) 0.16 (0.2) 0.035 (0.8) 0.14 (0.3) 0.069 (0.6) 0.11 (0.4) −0.14 (0.3) 0.2 (0.1) 0.28 (0.04) 0.17 (0.2) 0.27 (0.04) 0.19 (0.2) 0.067 (0.6) 0.26 (0.05) 0.069 (0.6) 0.12 (0.4) 0.15 (0.3) 0.16 (0.2)
0.07 (0.6) −0.028 (0.8) −0.055 (0.7) 0.12 (0.4) 0.13 (0.3) −0.13 (0.3) 0.18 (0.2) 0.11 (0.4) −0.024 (0.9) 0.28 (0.04) 0.23 (0.09) 0.11 (0.4) 0.18 (0.2) 0.072 (0.6) 0.3 (0.02) 0.11 (0.4) 0.11 (0.4) 0.15 (0.3) 0.21 (0.1) 0.22 (0.1) 0.3 (0.02) 0.2 (0.1) 0.3 (0.02) 0.19 (0.2) 0.14 (0.3) −0.012 (0.9) −0.0081 (1) −0.005 (1) 0.085 (0.5) −0.026 (0.8)
−0.15 (0.3) −0.22 (0.1) −0.086 (0.5) 0.0036 (1) 0.13 (0.3) −0.077 (0.6) −0.061 (0.7) −0.019 (0.9) −0.23 (0.08) −0.045 (0.7) 0.041 (0.8) −0.041 (0.8) 0.034 (0.8) 0.039 (0.8) 0.052 (0.7) 0.0092 (0.9) 0.011 (0.9) 0.073 (0.6) −0.1 (0.4) 0.063 (0.6) 0.15 (0.3) 0.04 (0.8) 0.14 (0.3) 0.081 (0.6) 0.034 (0.8) −0.082 (0.5) −0.069 (0.6) −0.079 (0.6) −0.013 (0.9) 0.028 (0.8)
0.21 (0.1) 0.22 (0.1) −0.11 (0.4) 0.058 (0.7) −0.1 (0.4) −0.084 (0.5) 0.11 (0.4) −0.023 (0.9) 0.2 (0.1) 0.27 (0.04) 0.055 (0.7) 0.07 (0.6) 0.031 (0.8) −0.0015 (1) 0.12 (0.4) 0.056 (0.7) 0.054 (0.7) 0.027 (0.8) 0.28 (0.04) 0.1 (0.4) 0.052 (0.7) 0.055 (0.7) 0.048 (0.7) 0.015 (0.9) −0.026 (0.9) 0.11 (0.4) 0.031 (0.8) 0.065 (0.6) 0.072 (0.6) −0.13 (0.3)
0.043 (0.8) 0.058 (0.7) 0.15 (0.3) 0.043 (0.8) −0.14 (0.3) −0.14 (0.3) 0.13 (0.4) 0.12 (0.4) 0.1 (0.5) 0.072 (0.6) 0.12 (0.4) 0.12 (0.4) 0.063 (0.6) −0.11 (0.4) 0.14 (0.3) −0.015 (0.9) 0.0096 (0.9) −0.022 (0.9) 0.27 (0.04) −0.034 (0.8) −0.11 (0.4) −0.036 (0.8) −0.06 (0.7) −0.071 (0.6) 0.0059 (1) −0.14 (0.3) −0.04 (0.8) −0.041 (0.8) −0.051 (0.7) −0.15 (0.3)
−0.35 (0.008) −0.31 (0.02) −0.025 (0.9) −0.023 (0.9) −0.37 (0.005) −0.11 (0.4) −0.33 (0.01) −0.14 (0.3) −0.11 (0.4) −0.35 (0.008) −0.24 (0.08) −0.12 (0.4) −0.24 (0.08) −0.15 (0.3) −0.38 (0.004) −0.15 (0.3) −0.26 (0.05) −0.066 (0.6) −0.2 (0.1) −0.39 (0.003) −0.45 (4e−04) −0.42 (0.001) −0.39 (0.003) −0.31 (0.02) −0.24 (0.07) −0.19 (0.2) −0.21 (0.1) −0.21 (0.1) −0.31 (0.02) −0.11 (0.4)
−0.37 (0.004) −0.35 (0.008) −0.038 (0.8) −0.24 (0.08) −0.13 (0.3) −0.2 (0.1) −0.32 (0.02) −0.18 (0.2) −0.29 (0.03) −0.41 (0.002) −0.2 (0.1) −0.16 (0.2) −0.17 (0.2) −0.29 (0.03) −0.2 (0.1) −0.3 (0.03) −0.18 (0.2) −0.25 (0.06) −0.17 (0.2) −0.27 (0.05) −0.47 (3e−04) −0.31 (0.02) −0.33 (0.01) −0.35 (0.008) −0.31 (0.02) −0.41 (0.002) −0.25 (0.06) −0.29 (0.03) −0.32 (0.01) −0.19 (0.2)
−0.3 (0.03) −0.27 (0.05) 0.15 (0.3) −0.075 (0.6) −0.3 (0.03) −0.17 (0.2) −0.18 (0.2) 0.0064 (1) −0.11 (0.4) −0.34 (0.01) −0.092 (0.5) −0.019 (0.9) −0.12 (0.4) −0.23 (0.1) −0.2 (0.1) −0.18 (0.2) −0.19 (0.2) −0.11 (0.4) −0.037 (0.8) −0.36 (0.007) −0.48 (2e−04) −0.35 (0.008) −0.38 (0.003) −0.31 (0.02) −0.16 (0.2) −0.33 (0.01) −0.18 (0.2) −0.22 (0.1) −0.3 (0.03) −0.16 (0.2)
−0.12 (0.4) −0.099 (0.5) 0.34 (0.01) −0.05 (0.7) 0.1 (0.5) 0.015 (0.9) 0.09 (0.5) 0.18 (0.2) −0.079 (0.6) −0.27 (0.04) 0.12 (0.4) 0.15 (0.3) 0.11 (0.4) 0.002 (1) 0.063 (0.6) −0.047 (0.7) 0.032 (0.8) −0.052 (0.7) 0.1 (0.4) −0.071 (0.6) −0.11 (0.4) −0.03 (0.8) −0.07 (0.6) −0.032 (0.8) 0.088 (0.5) −0.19 (0.2) 0.013 (0.9) −0.008 (1) −0.055 (0.7) 0.0027 (1)
0.31 (0.02) 0.23 (0.09) −0.12 (0.4) 0.045 (0.7) 0.16 (0.2) 0.22 (0.1) 0.049 (0.7) −0.0074 (1) 0.13 (0.3) 0.3 (0.03) 0.11 (0.4) 0.045 (0.7) 0.09 (0.5) 0.23 (0.09) 0.074 (0.6) 0.22 (0.1) 0.11 (0.4) 0.24 (0.08) −0.057 (0.7) 0.29 (0.03) 0.27 (0.04) 0.17 (0.2) 0.29 (0.03) 0.22 (0.1) 0.14 (0.3) 0.35 (0.009) 0.22 (0.1) 0.13 (0.3) 0.29 (0.03) 0.14 (0.3)

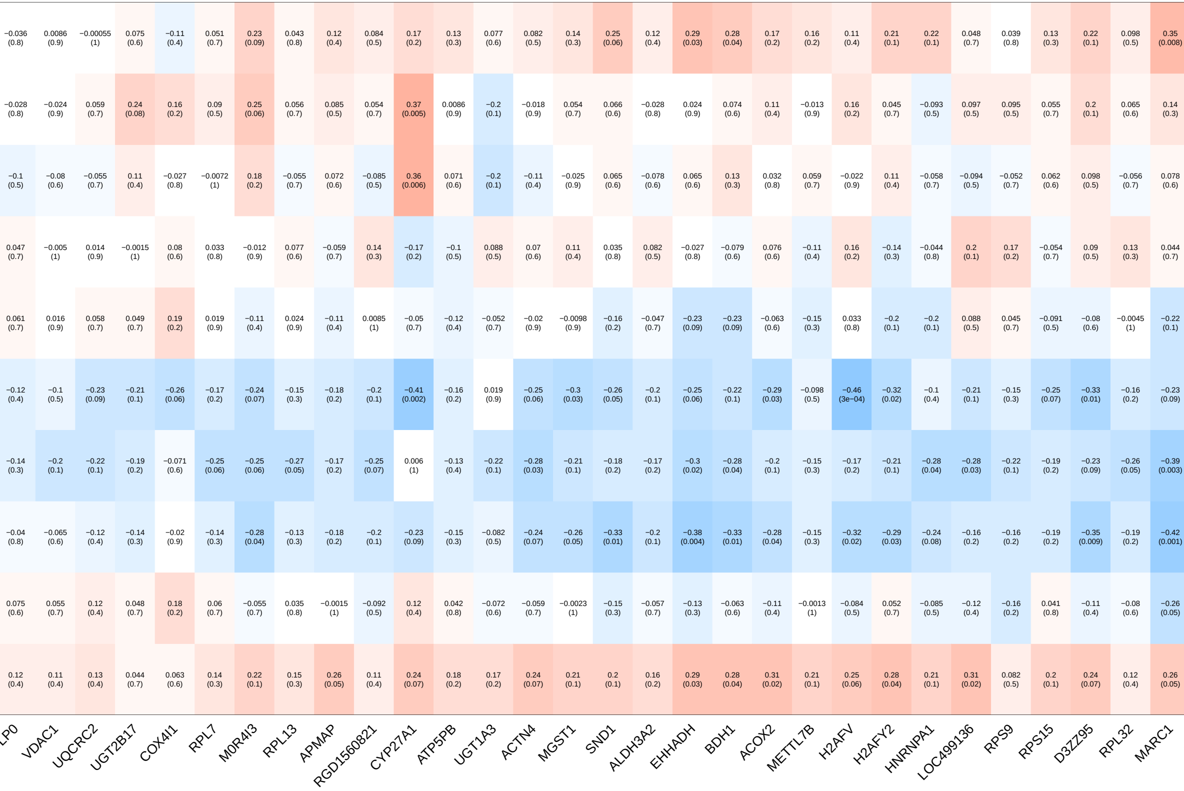

−0.036 (0.8) 0.0086 (0.9) −0.00055 (1) 0.075 (0.6) −0.11 (0.4) 0.051 (0.7) 0.23 (0.09) 0.043 (0.8) 0.12 (0.4) 0.084 (0.5) 0.17 (0.2) 0.13 (0.3) 0.077 (0.6) 0.082 (0.5) 0.14 (0.3) 0.25 (0.06) 0.12 (0.4) 0.29 (0.03) 0.28 (0.04) 0.17 (0.2) 0.16 (0.2) 0.11 (0.4) 0.21 (0.1) 0.22 (0.1) 0.048 (0.7) 0.039 (0.8) 0.13 (0.3) 0.22 (0.1) 0.098 (0.5) 0.35 (0.008)
−0.028 (0.8) −0.024 (0.9) 0.059 (0.7) 0.24 (0.08) 0.16 (0.2) 0.09 (0.5) 0.25 (0.06) 0.056 (0.7) 0.085 (0.5) 0.054 (0.7) 0.37 (0.005) 0.0086 (0.9) −0.2 (0.1) −0.018 (0.9) 0.054 (0.7) 0.066 (0.6) −0.028 (0.8) 0.024 (0.9) 0.074 (0.6) 0.11 (0.4) −0.013 (0.9) 0.16 (0.2) 0.045 (0.7) −0.093 (0.5) 0.097 (0.5) 0.095 (0.5) 0.055 (0.7) 0.2 (0.1) 0.065 (0.6) 0.14 (0.3)
−0.1 (0.5) −0.08 (0.6) −0.055 (0.7) 0.11 (0.4) −0.027 (0.8) −0.0072 (1) 0.18 (0.2) −0.055 (0.7) 0.072 (0.6) −0.085 (0.5) 0.36 (0.006) 0.071 (0.6) −0.2 (0.1) −0.11 (0.4) −0.025 (0.9) 0.065 (0.6) −0.078 (0.6) 0.065 (0.6) 0.13 (0.3) 0.032 (0.8) 0.059 (0.7) −0.022 (0.9) 0.11 (0.4) −0.058 (0.7) −0.094 (0.5) −0.052 (0.7) 0.062 (0.6) 0.098 (0.5) −0.056 (0.7) 0.078 (0.6)
0.047 (0.7) −0.005 (1) 0.014 (0.9) −0.0015 (1) 0.08 (0.6) 0.033 (0.8) −0.012 (0.9) 0.077 (0.6) −0.059 (0.7) 0.14 (0.3) −0.17 (0.2) −0.1 (0.5) 0.088 (0.5) 0.07 (0.6) 0.11 (0.4) 0.035 (0.8) 0.082 (0.5) −0.027 (0.8) −0.079 (0.6) 0.076 (0.6) −0.11 (0.4) 0.16 (0.2) −0.14 (0.3) −0.044 (0.8) 0.2 (0.1) 0.17 (0.2) −0.054 (0.7) 0.09 (0.5) 0.13 (0.3) 0.044 (0.7)
0.061 (0.7) 0.016 (0.9) 0.058 (0.7) 0.049 (0.7) 0.19 (0.2) 0.019 (0.9) −0.11 (0.4) 0.024 (0.9) −0.11 (0.4) 0.0085 (1) −0.05 (0.7) −0.12 (0.4) −0.052 (0.7) −0.02 (0.9) −0.0098 (0.9) −0.16 (0.2) −0.047 (0.7) −0.23 (0.09) −0.23 (0.09) −0.063 (0.6) −0.15 (0.3) 0.033 (0.8) −0.2 (0.1) −0.2 (0.1) 0.088 (0.5) 0.045 (0.7) −0.091 (0.5) −0.08 (0.6) −0.0045 (1) −0.22 (0.1)
−0.12 (0.4) −0.1 (0.5) −0.23 (0.09) −0.21 (0.1) −0.26 (0.06) −0.17 (0.2) −0.24 (0.07) −0.15 (0.3) −0.18 (0.2) −0.2 (0.1) −0.41 (0.002) −0.16 (0.2) 0.019 (0.9) −0.25 (0.06) −0.3 (0.03) −0.26 (0.05) −0.2 (0.1) −0.25 (0.06) −0.22 (0.1) −0.29 (0.03) −0.098 (0.5) −0.46 (3e−04) −0.32 (0.02) −0.1 (0.4) −0.21 (0.1) −0.15 (0.3) −0.25 (0.07) −0.33 (0.01) −0.16 (0.2) −0.23 (0.09)
−0.14 (0.3) −0.2 (0.1) −0.22 (0.1) −0.19 (0.2) −0.071 (0.6) −0.25 (0.06) −0.25 (0.06) −0.27 (0.05) −0.17 (0.2) −0.25 (0.07) 0.006 (1) −0.13 (0.4) −0.22 (0.1) −0.28 (0.03) −0.21 (0.1) −0.18 (0.2) −0.17 (0.2) −0.3 (0.02) −0.28 (0.04) −0.2 (0.1) −0.15 (0.3) −0.17 (0.2) −0.21 (0.1) −0.28 (0.04) −0.28 (0.03) −0.22 (0.1) −0.19 (0.2) −0.23 (0.09) −0.26 (0.05) −0.39 (0.003)
−0.04 (0.8) −0.065 (0.6) −0.12 (0.4) −0.14 (0.3) −0.02 (0.9) −0.14 (0.3) −0.28 (0.04) −0.13 (0.3) −0.18 (0.2) −0.2 (0.1) −0.23 (0.09) −0.15 (0.3) −0.082 (0.5) −0.24 (0.07) −0.26 (0.05) −0.33 (0.01) −0.2 (0.1) −0.38 (0.004) −0.33 (0.01) −0.28 (0.04) −0.15 (0.3) −0.32 (0.02) −0.29 (0.03) −0.24 (0.08) −0.16 (0.2) −0.16 (0.2) −0.19 (0.2) −0.35 (0.009) −0.19 (0.2) −0.42 (0.001)
0.075 (0.6) 0.055 (0.7) 0.12 (0.4) 0.048 (0.7) 0.18 (0.2) 0.06 (0.7) −0.055 (0.7) 0.035 (0.8) −0.0015 (1) −0.092 (0.5) 0.12 (0.4) 0.042 (0.8) −0.072 (0.6) −0.059 (0.7) −0.0023 (1) −0.15 (0.3) −0.057 (0.7) −0.13 (0.3) −0.063 (0.6) −0.11 (0.4) −0.0013 (1) −0.084 (0.5) 0.052 (0.7) −0.085 (0.5) −0.12 (0.4) −0.16 (0.2) 0.041 (0.8) −0.11 (0.4) −0.08 (0.6) −0.26 (0.05)
0.12 (0.4) 0.11 (0.4) 0.13 (0.4) 0.044 (0.7) 0.063 (0.6) 0.14 (0.3) 0.22 (0.1) 0.15 (0.3) 0.26 (0.05) 0.11 (0.4) 0.24 (0.07) 0.18 (0.2) 0.17 (0.2) 0.24 (0.07) 0.21 (0.1) 0.2 (0.1) 0.16 (0.2) 0.29 (0.03) 0.28 (0.04) 0.31 (0.02) 0.21 (0.1) 0.25 (0.06) 0.28 (0.04) 0.21 (0.1) 0.31 (0.02) 0.082 (0.5) 0.2 (0.1) 0.24 (0.07) 0.12 (0.4) 0.26 (0.05)
LP0
VDAC1
UQCRC2
UGT2B17
COX4I1
RPL7
M0R4I3
RPL13
APMAP
RGD1560821
CYP27A1
ATP5PB
UGT1A3
ACTN4
MGST1
SND1
ALDH3A2
EHHADH
BDH1
ACOX2
METTL7B
H2AFV
H2AFY2
HNRNPA1
LOC499136
RPS9
RPS15
D3ZZ95
RPL32
MARC1

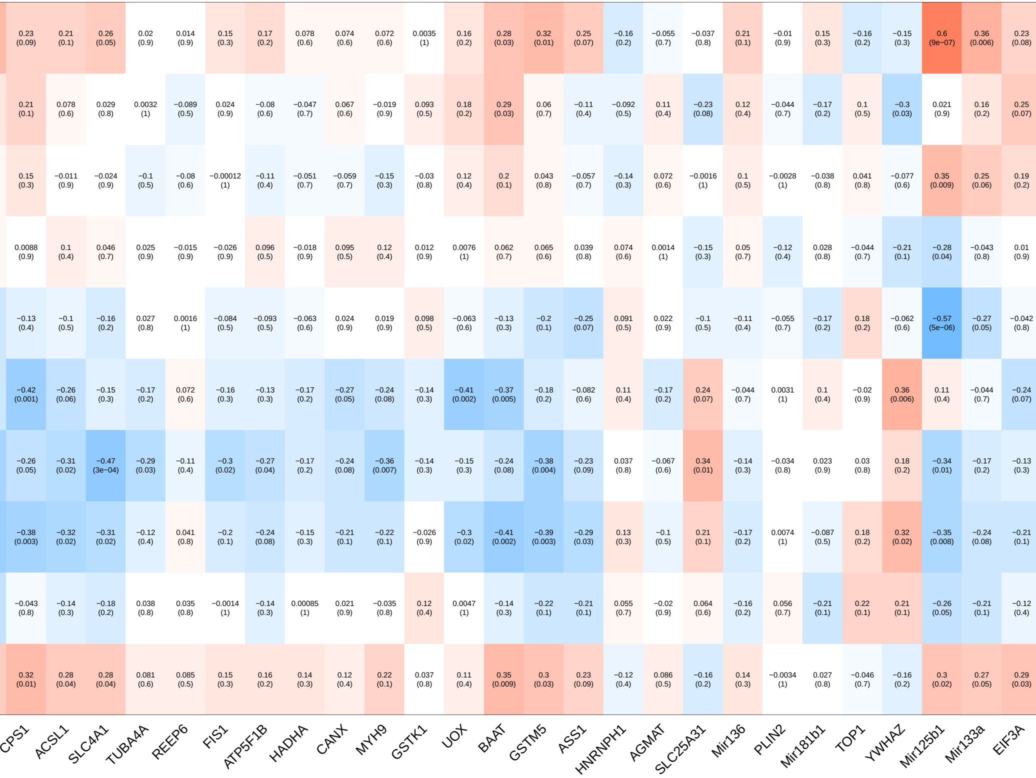
0.23 (0.09) 0.21 (0.1) 0.26 (0.05) 0.02 (0.9) 0.014 (0.9) 0.15 (0.3) 0.17 (0.2) 0.078 (0.6) 0.074 (0.6) 0.072 (0.6) 0.0035 (1) 0.16 (0.2) 0.28 (0.03) 0.32 (0.01) 0.25 (0.07) −0.16 (0.2) −0.055 (0.7) −0.037 (0.8) 0.21 (0.1) −0.01 (0.9) 0.15 (0.3) −0.16 (0.2) −0.15 (0.3) 0.6 (9e−07) 0.36 (0.006) 0.23 (0.08)
0.21 (0.1) 0.078 (0.6) 0.029 (0.8) 0.0032 (1) −0.089 (0.5) 0.024 (0.9) −0.08 (0.6) −0.047 (0.7) 0.067 (0.6) −0.019 (0.9) 0.093 (0.5) 0.18 (0.2) 0.29 (0.03) 0.06 (0.7) −0.11 (0.4) −0.092 (0.5) 0.11 (0.4) −0.23 (0.08) 0.12 (0.4) −0.044 (0.7) −0.17 (0.2) 0.1 (0.5) −0.3 (0.03) 0.021 (0.9) 0.16 (0.2) 0.25 (0.07)
0.15 (0.3) −0.011 (0.9) −0.024 (0.9) −0.1 (0.5) −0.08 (0.6) −0.00012 (1) −0.11 (0.4) −0.051 (0.7) −0.059 (0.7) −0.15 (0.3) −0.03 (0.8) 0.12 (0.4) 0.2 (0.1) 0.043 (0.8) −0.057 (0.7) −0.14 (0.3) 0.072 (0.6) −0.0016 (1) 0.1 (0.5) −0.0028 (1) −0.038 (0.8) 0.041 (0.8) −0.077 (0.6) 0.35 (0.009) 0.25 (0.06) 0.19 (0.2)
0.0088 (0.9) 0.1 (0.4) 0.046 (0.7) 0.025 (0.9) −0.015 (0.9) −0.026 (0.9) 0.096 (0.5) −0.018 (0.9) 0.095 (0.5) 0.12 (0.4) 0.012 (0.9) 0.0076 (1) 0.062 (0.7) 0.065 (0.6) 0.039 (0.8) 0.074 (0.6) 0.0014 (1) −0.15 (0.3) 0.05 (0.7) −0.12 (0.4) 0.028 (0.8) −0.044 (0.7) −0.21 (0.1) −0.28 (0.04) −0.043 (0.8) 0.01 (0.9)
−0.13 (0.4) −0.1 (0.5) −0.16 (0.2) 0.027 (0.8) 0.0016 (1) −0.084 (0.5) −0.093 (0.5) −0.063 (0.6) 0.024 (0.9) 0.019 (0.9) 0.098 (0.5) −0.063 (0.6) −0.13 (0.3) −0.2 (0.1) −0.25 (0.07) 0.091 (0.5) 0.022 (0.9) −0.1 (0.5) −0.11 (0.4) −0.055 (0.7) −0.17 (0.2) 0.18 (0.2) −0.062 (0.6) −0.57 (5e−06) −0.27 (0.05) −0.042 (0.8)
−0.42 (0.001) −0.26 (0.06) −0.15 (0.3) −0.17 (0.2) 0.072 (0.6) −0.16 (0.3) −0.13 (0.3) −0.17 (0.2) −0.27 (0.05) −0.24 (0.08) −0.14 (0.3) −0.41 (0.002) −0.37 (0.005) −0.18 (0.2) −0.082 (0.6) 0.11 (0.4) −0.17 (0.2) 0.24 (0.07) −0.044 (0.7) 0.0031 (1) 0.1 (0.4) −0.02 (0.9) 0.36 (0.006) 0.11 (0.4) −0.044 (0.7) −0.24 (0.07)
−0.26 (0.05) −0.31 (0.02) −0.47 (3e−04) −0.29 (0.03) −0.11 (0.4) −0.3 (0.02) −0.27 (0.04) −0.17 (0.2) −0.24 (0.08) −0.36 (0.007) −0.14 (0.3) −0.15 (0.3) −0.24 (0.08) −0.38 (0.004) −0.23 (0.09) 0.037 (0.8) −0.067 (0.6) 0.34 (0.01) −0.14 (0.3) −0.034 (0.8) 0.023 (0.9) 0.03 (0.8) 0.18 (0.2) −0.34 (0.01) −0.17 (0.2) −0.13 (0.3)
−0.38 (0.003) −0.32 (0.02) −0.31 (0.02) −0.12 (0.4) 0.041 (0.8) −0.2 (0.1) −0.24 (0.08) −0.15 (0.3) −0.21 (0.1) −0.22 (0.1) −0.026 (0.9) −0.3 (0.02) −0.41 (0.002) −0.39 (0.003) −0.29 (0.03) 0.13 (0.3) −0.1 (0.5) 0.21 (0.1) −0.17 (0.2) 0.0074 (1) −0.087 (0.5) 0.18 (0.2) 0.32 (0.02) −0.35 (0.008) −0.24 (0.08) −0.21 (0.1)
−0.043 (0.8) −0.14 (0.3) −0.18 (0.2) 0.038 (0.8) 0.035 (0.8) −0.0014 (1) −0.14 (0.3) 0.00085 (1) 0.021 (0.9) −0.035 (0.8) 0.12 (0.4) 0.0047 (1) −0.14 (0.3) −0.22 (0.1) −0.21 (0.1) 0.055 (0.7) −0.02 (0.9) 0.064 (0.6) −0.16 (0.2) 0.056 (0.7) −0.21 (0.1) 0.22 (0.1) 0.21 (0.1) −0.26 (0.05) −0.21 (0.1) −0.12 (0.4)
0.32 (0.01) 0.28 (0.04) 0.28 (0.04) 0.081 (0.6) 0.085 (0.5) 0.15 (0.3) 0.16 (0.2) 0.14 (0.3) 0.12 (0.4) 0.22 (0.1) 0.037 (0.8) 0.11 (0.4) 0.35 (0.009) 0.3 (0.03) 0.23 (0.09) −0.12 (0.4) 0.086 (0.5) −0.16 (0.2) 0.14 (0.3) −0.0034 (1) 0.027 (0.8) −0.046 (0.7) −0.16 (0.2) 0.3 (0.02) 0.27 (0.05) 0.29 (0.03)
CPS1
ACSL1
SLC4A1
TUBA4A
REEP6
FIS1
ATP5F1B
HADHA
CANX
MYH9
GSTK1
UOX
BAAT
GSTM5
ASS1
HNRNPH1
AGMAT
SLC25A31
Mir136
PLIN2
Mir181b1
TOP1
YWHAZ
Mir125b1
Mir133a
EIF3A

Citric	ANK2	RAB14	PGRMC2	ATP2A3	RGD1559459	ACLY	Mir200a	D4A2X2	ATL3	DPP4	IQGAP3	F213A	R9PY08	PHYH	PRODH1	GLYAT	CYP1A1	CYP2S1	ACSL5	CYP2E1	MARC2	EPHX1	ACAD11	SLC2A2	FMO9	SQOR	CYP2C11	SCCPDH	TECR
0.026 (0.8)	−0.14 (0.3)	−0.085 (0.5)	0.082 (0.5)	−0.00055 (1)	0.068 (0.6)	0.34 (0.01)	0.19 (0.2)	0.2 (0.1)	0.19 (0.2)	0.17 (0.2)	0.29 (0.03)	0.016 (0.9)	0.16 (0.2)	0.06 (0.7)	0.35 (0.008)	0.29 (0.03)	0.087 (0.5)	0.15 (0.3)	0.32 (0.01)	0.28 (0.03)	0.21 (0.1)	−0.072 (0.6)	0.17 (0.2)	0.41 (0.002)	0.085 (0.5)	0.048 (0.7)	0.41 (0.002)	0.31 (0.02)	0.31 (0.02)
0.11 (0.4)	−0.34 (0.009)	0.11 (0.4)	0.15 (0.3)	0.25 (0.07)	0.14 (0.3)	0.085 (0.5)	−0.019 (0.9)	0.14 (0.3)	0.063 (0.6)	−0.052 (0.7)	0.072 (0.6)	−0.018 (0.9)	0.078 (0.6)	0.0091 (0.9)	0.15 (0.3)	−0.03 (0.8)	−0.29 (0.03)	−0.13 (0.3)	−0.17 (0.2)	−0.069 (0.6)	−0.27 (0.05)	−0.3 (0.03)	−0.16 (0.2)	0.26 (0.05)	0.074 (0.6)	0.048 (0.7)	0.12 (0.4)	0.24 (0.07)	0.11 (0.4)
0.31 (0.02)	−0.22 (0.1)	0.062 (0.6)	0.11 (0.4)	0.019 (0.9)	0.2 (0.1)	0.24 (0.08)	0.13 (0.3)	0.16 (0.2)	0.093 (0.5)	−0.041 (0.8)	0.03 (0.8)	−0.047 (0.7)	−0.0051 (1)	−0.083 (0.5)	0.16 (0.2)	0.25 (0.07)	0.023 (0.9)	0.033 (0.8)	0.095 (0.5)	0.16 (0.2)	−0.12 (0.4)	−0.21 (0.1)	−0.016 (0.9)	0.24 (0.07)	0.06 (0.7)	0.032 (0.8)	0.19 (0.2)	0.24 (0.07)	0.13 (0.4)
−0.44 (8e−04)	−0.046 (0.7)	−0.022 (0.9)	−0.022 (0.9)	0.16 (0.2)	−0.13 (0.3)	−0.11 (0.4)	−0.12 (0.4)	−0.018 (0.9)	0.013 (0.9)	0.12 (0.4)	0.1 (0.5)	−0.0051 (1)	0.078 (0.6)	0.097 (0.5)	0.0029 (1)	−0.27 (0.05)	−0.3 (0.02)	−0.1 (0.4)	−0.22 (0.1)	−0.22 (0.1)	−0.064 (0.6)	−0.035 (0.8)	−0.12 (0.4)	−0.0032 (1)	0.061 (0.7)	0.042 (0.8)	−0.0092 (0.9)	−0.023 (0.9)	0.018 (0.9)
−0.11 (0.4)	−0.02 (0.9)	0.063 (0.6)	−0.052 (0.7)	0.2 (0.1)	−0.095 (0.5)	−0.3 (0.02)	−0.27 (0.05)	−0.13 (0.3)	−0.098 (0.5)	−0.17 (0.2)	−0.17 (0.2)	−0.018 (0.9)	−0.028 (0.8)	−0.024 (0.9)	−0.2 (0.1)	−0.35 (0.009)	−0.26 (0.05)	−0.18 (0.2)	−0.39 (0.003)	−0.31 (0.02)	−0.24 (0.07)	0.011 (0.9)	−0.22 (0.1)	−0.27 (0.04)	−0.051 (0.7)	−0.036 (0.8)	−0.27 (0.04)	−0.17 (0.2)	−0.21 (0.1)
0.11 (0.4)	0.18 (0.2)	−0.2 (0.1)	−0.12 (0.4)	−0.35 (0.008)	−0.36 (0.006)	−0.018 (0.9)	−0.071 (0.6)	−0.4 (0.002)	−0.052 (0.7)	−0.12 (0.4)	−0.38 (0.004)	−0.19 (0.2)	−0.35 (0.008)	−0.34 (0.01)	−0.16 (0.2)	−0.18 (0.2)	−0.002 (1)	−0.014 (0.9)	−0.031 (0.8)	−0.075 (0.6)	0.0026 (1)	0.12 (0.4)	−0.089 (0.5)	−0.33 (0.01)	−0.059 (0.7)	−0.2 (0.1)	−0.24 (0.07)	−0.28 (0.04)	−0.24 (0.07)
0.1 (0.5)	0.087 (0.5)	0.076 (0.6)	−0.1 (0.5)	−0.072 (0.6)	−0.0085 (1)	−5e−04 (1)	0.051 (0.7)	−0.011 (0.9)	−0.13 (0.3)	−0.23 (0.09)	−0.29 (0.03)	−0.22 (0.1)	−0.27 (0.05)	−0.18 (0.2)	−0.27 (0.04)	0.039 (0.8)	0.11 (0.4)	−0.016 (0.9)	−0.095 (0.5)	−0.044 (0.7)	−0.14 (0.3)	0.0084 (1)	−0.15 (0.3)	−0.36 (0.006)	0.0044 (1)	−0.048 (0.7)	−0.25 (0.07)	−0.14 (0.3)	−0.22 (0.1)
0.17 (0.2)	0.18 (0.2)	−0.023 (0.9)	−0.13 (0.3)	−0.12 (0.4)	−0.23 (0.09)	−0.22 (0.1)	−0.18 (0.2)	−0.3 (0.02)	−0.15 (0.3)	−0.31 (0.02)	−0.46 (4e−04)	−0.14 (0.3)	−0.3 (0.02)	−0.26 (0.06)	−0.34 (0.01)	−0.29 (0.03)	−0.034 (0.8)	−0.11 (0.4)	−0.26 (0.05)	−0.2 (0.1)	−0.19 (0.2)	0.11 (0.4)	−0.19 (0.2)	−0.48 (2e−04)	−0.094 (0.5)	−0.14 (0.3)	−0.42 (0.001)	−0.33 (0.01)	−0.36 (0.006)
0.36 (0.006)	0.18 (0.2)	0.18 (0.2)	−0.063 (0.6)	0.02 (0.9)	0.19 (0.2)	−0.22 (0.1)	0.009 (0.9)	−0.04 (0.8)	−0.16 (0.2)	−0.25 (0.06)	−0.23 (0.08)	0.032 (0.8)	−0.059 (0.7)	−0.098 (0.5)	−0.11 (0.4)	−0.13 (0.4)	0.11 (0.4)	−0.083 (0.5)	−0.17 (0.2)	−0.083 (0.5)	−0.12 (0.4)	0.08 (0.6)	−0.069 (0.6)	−0.25 (0.06)	−0.1 (0.5)	−0.057 (0.7)	−0.24 (0.07)	−0.15 (0.3)	−0.24 (0.07)
0.047 (0.7)	−0.22 (0.1)	0.074 (0.6)	0.11 (0.4)	0.18 (0.2)	0.1 (0.5)	0.29 (0.03)	0.38 (0.004)	0.18 (0.2)	0.2 (0.1)	0.2 (0.1)	0.37 (0.005)	0.13 (0.3)	0.063 (0.6)	0.16 (0.3)	0.3 (0.02)	0.2 (0.1)	0.097 (0.5)	0.057 (0.7)	0.17 (0.2)	0.14 (0.3)	0.055 (0.7)	−0.067 (0.6)	0.14 (0.3)	0.36 (0.006)	0.063 (0.6)	0.12 (0.4)	0.36 (0.007)	0.26 (0.05)	0.31 (0.02)

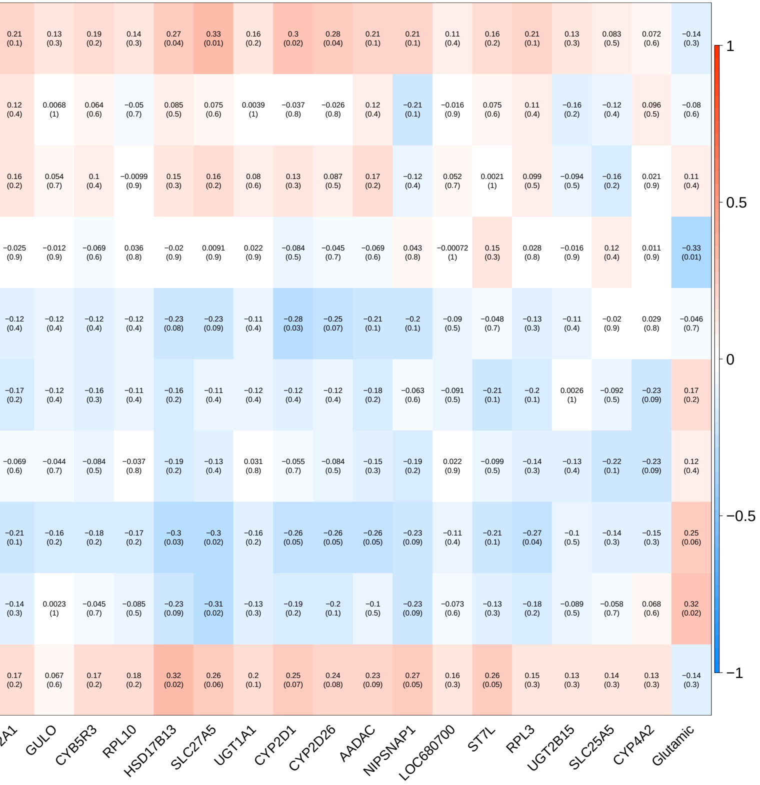
0.21 (0.1) 0.13 (0.3) 0.19 (0.2) 0.14 (0.3) 0.27 (0.04) 0.33 (0.01) 0.16 (0.2) 0.3 (0.02) 0.28 (0.04) 0.21 (0.1) 0.21 (0.1) 0.11 (0.4) 0.16 (0.2) 0.21 (0.1) 0.13 (0.3) 0.083 (0.5) 0.072 (0.6) −0.14 (0.3)
0.12 (0.4) 0.0068 (1) 0.064 (0.6) −0.05 (0.7) 0.085 (0.5) 0.075 (0.6) 0.0039 (1) −0.037 (0.8) −0.026 (0.8) 0.12 (0.4) −0.21 (0.1) −0.016 (0.9) 0.075 (0.6) 0.11 (0.4) −0.16 (0.2) −0.12 (0.4) 0.096 (0.5) −0.08 (0.6)
0.16 (0.2) 0.054 (0.7) 0.1 (0.4) −0.0099 (0.9) 0.15 (0.3) 0.16 (0.2) 0.08 (0.6) 0.13 (0.3) 0.087 (0.5) 0.17 (0.2) −0.12 (0.4) 0.052 (0.7) 0.0021 (1) 0.099 (0.5) −0.094 (0.5) −0.16 (0.2) 0.021 (0.9) 0.11 (0.4)
−0.025 (0.9) −0.012 (0.9) −0.069 (0.6) 0.036 (0.8) −0.02 (0.9) 0.0091 (0.9) 0.022 (0.9) −0.084 (0.5) −0.045 (0.7) −0.069 (0.6) 0.043 (0.8) −0.00072 (1) 0.15 (0.3) 0.028 (0.8) −0.016 (0.9) 0.12 (0.4) 0.011 (0.9) −0.33 (0.01)
−0.12 (0.4) −0.12 (0.4) −0.12 (0.4) −0.12 (0.4) −0.23 (0.08) −0.23 (0.09) −0.11 (0.4) −0.28 (0.03) −0.25 (0.07) −0.21 (0.1) −0.2 (0.1) −0.09 (0.5) −0.048 (0.7) −0.13 (0.3) −0.11 (0.4) −0.02 (0.9) 0.029 (0.8) −0.046 (0.7)
−0.17 (0.2) −0.12 (0.4) −0.16 (0.3) −0.11 (0.4) −0.16 (0.2) −0.11 (0.4) −0.12 (0.4) −0.12 (0.4) −0.12 (0.4) −0.18 (0.2) −0.063 (0.6) −0.091 (0.5) −0.21 (0.1) −0.2 (0.1) 0.0026 (1) −0.092 (0.5) −0.23 (0.09) 0.17 (0.2)
−0.069 (0.6) −0.044 (0.7) −0.084 (0.5) −0.037 (0.8) −0.19 (0.2) −0.13 (0.4) 0.031 (0.8) −0.055 (0.7) −0.084 (0.5) −0.15 (0.3) −0.19 (0.2) 0.022 (0.9) −0.099 (0.5) −0.14 (0.3) −0.13 (0.4) −0.22 (0.1) −0.23 (0.09) 0.12 (0.4)
−0.21 (0.1) −0.16 (0.2) −0.18 (0.2) −0.17 (0.2) −0.3 (0.03) −0.3 (0.02) −0.16 (0.2) −0.26 (0.05) −0.26 (0.05) −0.26 (0.05) −0.23 (0.09) −0.11 (0.4) −0.21 (0.1) −0.27 (0.04) −0.1 (0.5) −0.14 (0.3) −0.15 (0.3) 0.25 (0.06)
−0.14 (0.3) 0.0023 (1) −0.045 (0.7) −0.085 (0.5) −0.23 (0.09) −0.31 (0.02) −0.13 (0.3) −0.19 (0.2) −0.2 (0.1) −0.1 (0.5) −0.23 (0.09) −0.073 (0.6) −0.13 (0.3) −0.18 (0.2) −0.089 (0.5) −0.058 (0.7) 0.068 (0.6) 0.32 (0.02)
0.17 (0.2) 0.067 (0.6) 0.17 (0.2) 0.18 (0.2) 0.32 (0.02) 0.26 (0.06) 0.2 (0.1) 0.25 (0.07) 0.24 (0.08) 0.23 (0.09) 0.27 (0.05) 0.16 (0.3) 0.26 (0.05) 0.15 (0.3) 0.13 (0.3) 0.14 (0.3) 0.13 (0.3) −0.14 (0.3)
2A1
GULO
CYB5R3
RPL10
HSD17B13
SLC27A5
UGT1A1
CYP2D1
CYP2D26
AADAC
NIPSNAP1
LOC680700
ST7L
RPL3
UGT2B15
SLC25A5
CYP4A2
Glutamic
1
0.5
0
−0.5
−1
